# Haemodynamic control of zonated liver function via instructive vascular Wnt signalling

**DOI:** 10.1038/s41467-026-76966-7

**Published:** 2026-08-22

**Authors:** Ki Hong Lee, Moritz Jakab, Alexey Uvarovskii, Dimitris Papageorgiou, Donato Inverso, Jingjing Shi, Maria Riedel, Stephanie Gehrs, Shubhada Kulkarni, Stefanie Bobe, Adnan Ali, Suchira Gallage, Sophia Siegmund, Johannes Gahn, Leon Blankenhorn, Michael Buettner, Gernot Poschet, Karsten Richter, Oksana Voloshanenko, Roxana Ola, Thomas Korff, Friedemann Kiefer, Mathias Heikenwalder, Michael Boutros, Christof Niehrs, Carolin Mogler, Jeroen Krijgsveld, Simon Anders, Hellmut G. Augustin

**Affiliations:** 1 European Center for Angioscience (ECAS), Medical Faculty Mannheim, Heidelberg University, Mannheim, Germany; 2 Vascular Oncology and Metastasis, German Cancer Research Center (DKFZ), Heidelberg, Germany; 3 Center for Molecular Biology Heidelberg (ZMBH), Heidelberg University, Heidelberg, Germany; 4 Proteomics of Stem Cells and Cancer, DKFZ, Heidelberg, Germany; 5 Medical Faculty, Heidelberg University, Heidelberg, Germany; 6 European Institute for Molecular Imaging (EIMI), University of Münster, Münster, Germany; 7 Gerhard-Domagk-Institute of Pathology, University Hospital Münster, Münster, Germany; 8 Division of Chronic Inflammation and Cancer, DKFZ, Heidelberg, Germany; 9 Institute for Interdisciplinary Research on Cancer Metabolism and Chronic Inflammation, M3-Research Institute for Malignome, Metabolome and Microbiome, University of Tübingen, Faculty of Medicine, Tübingen, Germany; 10 Institute of Physiology and Pathophysiology, Department of Cardiovascular Physiology, Heidelberg University, Heidelberg, Germany; 11 Cardiovascular Pharmacology, ECAS, Medical Faculty Mannheim, Heidelberg University, Mannheim, Germany; 12 Metabolomics Core Technology Platform, Centre for Organismal Studies (COS), Heidelberg University, Heidelberg, Germany; 13 Electron Microscopy Core Facility, DKFZ, Heidelberg, Germany; 14 Signaling and Functional Genomics, DKFZ, Heidelberg, Germany; 15 Cell and Molecular Biology, Medical Faculty Mannheim, Heidelberg University, Mannheim, Germany; 16 German Centre for Cardiovascular Research (DZHK), Mannheim, Germany; 17 Molecular Embryology, DKFZ-ZMBH Alliance, DKFZ, Heidelberg, Germany; 18 Institute of Molecular Biology (IMB), Mainz, Germany; 19 Institute of Pathology, School of Medicine and Health, Technical University Munich, Munich, Germany; 20 BioQuant Center, Heidelberg University, Heidelberg, Germany

**Keywords:** Liver, Cardiovascular biology

## Abstract

The vascular endothelium forms a systemically disseminated organ that translates micromilieu factors into instructive cues, designated as angiocrine signalling. While vascular control of organ function mechanisms have been discovered in essentially all major organs, the mechanisms of translating milieu factors into angiocrine signalling mechanisms remain largely elusive. Here, focussing on the well-established angiocrine Wnt signalling-regulated liver metabolic zonation paradigm, we identified blood flow-induced haemodynamic stress as a biophysical sensor that governs the angiocrine expression profile of liver sinusoidal endothelial cells (LSEC). Combining single-cell RNA sequencing with spatial proteomics, we generated a high-resolution crosstalk map of LSEC and hepatocytes from LSEC Wnt-deficient mutant mice and littermate controls. Intriguingly, vascular Wnt receptors, in parallel with angiocrine Wnt ligands, were specifically enriched in pericentral LSEC, enforcing the spatially coordinated vascular Wnt functions. Consequently, vascular Wnt further modulated angiocrine gene signatures, LSEC morphology, and the expression of gap junctional molecules in an autocrine manner. Taken together, the data define LSEC as a dynamic cellular decoder that translates biophysical forces into instructive angiocrine signals via activating promiscuous vascular Wnt factors.

## Abstract (web summary)

The study identified blood flow as the key signal that programs liver blood vessels to instruct liver function through WNT signalling.

## Introduction

Hepatic function is precisely regulated by the cellular building blocks that form the innermost lining of the hepatic vasculature, the liver sinusoidal endothelial cells (LSEC)^1^. Being strategically positioned at the interface of the circulation and surrounding stroma, liver endothelial cells (EC) sense dynamic changes in fluctuating milieu factors and display a remarkable adaptive plasticity towards graded biophysical cues, including hypoxia, oxidative stress, and haemodynamic forces^2,3^. Thus, the hepatic endothelium has emerged as an active gatekeeper of homeostatic liver function by decoding exogenous stimuli into instructive EC-derived angiocrine factors, which ultimately regulate organ size and regeneration, host defence and the metabolic fate of hepatocytes^4–12^. Given the crucial roles of LSEC in shaping liver function, recent studies have focused on key organotypic transcription factors that modulate LSEC fate and have developed robust protocols to generate LSEC for therapeutic purposes^13–15^.

Recent advances in single cell genomics have permitted the dissection of cellular heterogeneity. Most of hepatic cell types, including hepatocytes and LSEC were identified to express gradual gene expression programmes alongside the liver sinusoids^16,17^. This zonation pattern coincides with biophysical gradients, which are established by the unique features of the hepatic vasculature that is defined by a dual blood supply. Oxygenated blood entering the liver lobule through the hepatic artery is mixed with nutrient-rich blood from the portal vein in the liver sinusoidal vessels. Hepatocytes lining the sinusoids consume oxygen and metabolites resulting in the drainage of hypoxic and nutrient-depleted blood by the central vein. This gradient of availability in oxygen and macromolecules establishes the spatial division of labour in the liver, which is essential for hepatic function and, hence, systemic health. The balance between periportal and pericentral hepatic function orchestrates the total energy supply and xenobiotic metabolism^18^. Losing this balance, thus losing the metabolic zonation of the liver, eventually results in impaired hepatic function and  is associated with a poor prognosis in hepatic diseases such as hepatocellular carcinoma or leads to metabolic dysfunction such as hyperammonaemia^19,20^. Understanding how liver zonation is established and maintained is therefore of high pathophysiological relevance.

Pericentral LSEC are major determinants of hepatic function and the spatial coordination of the hepatic architecture by secreting morphogenic Wnt factors Wnt2, Wnt9b, and the Wnt potentiator R-spondin3 (Rspo3)^4,9–12^. These vascular Wnt factors establish and sustain a highly metabolic pericentral hepatocyte niche and they have been shown to be regulated by the endothelial-specific receptor tyrosine kinase Tie1^21,22^. In fact, one-third of the endothelial transcriptome and roughly 25% of the endothelial proteome were found to be zonated alongside the portal-central venous axis^22^. While hepatic zonation and its consequences have been characterised in substantial detail, microenvironmental factors that establish zonated gene programmes remain elusive. Indirect evidence implies that mechanical stimuli may trigger the expression of vascular Wnt factors in the liver^23,24^. Yet, a systematic investigation of biophysical factors that enforce hepatic zonation has not been conducted.

## Results

### Blood flow-derived haemodynamic stimuli induce vascular Wnt expression

**Fig. 1:**
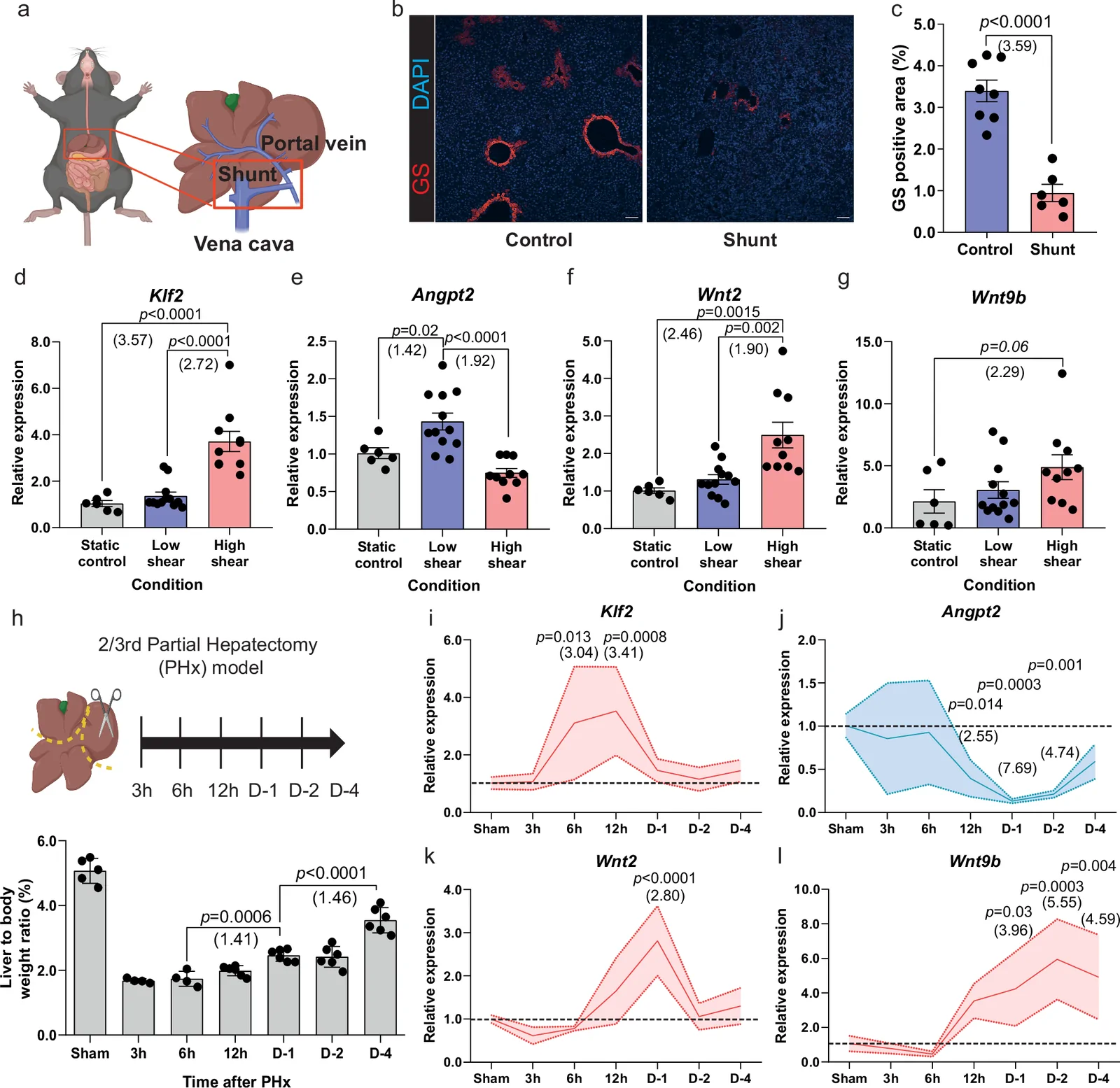
Blood flow-derived biomechanical stimuli modulate vascular Wnt signalling. **a** Illustration of a congenital portosystemic shunt. Aberrant vessel formation between the portal vein and vena cava hijacks blood flow routed to the liver. Created in BioRender. Lee, KH. (2026) https://BioRender.com/5olnoti. **b** Representative immunofluorescence image of glutamine synthetase (GS) in the liver of the shunt (*n* = 6) and control mouse (*n* = 8). Male mice were included in the study. Scale bars, 100 μm. **c** Percentage of GS positive area in the whole liver section (control, *n* = 8 mice; shunt, *n* = 6 mice). **d**–**g** Relative RT-qPCR gene expression analysis of *Klf2*, *Angpt2*, *Wnt2* and *Wnt9b* in wild-type primary LSEC freshly isolated from each mouse were cultured under different shear conditions (static control, *n* = 6; low shear condition, *n* = 12; high shear condition, *n* = 10). LSEC were isolated from both female and male mice. **h** Experimental scheme of 2/3rd partial hepatectomy (PHx) model. Created in BioRender. Lee, KH. (2026) https://BioRender.com/f7leoqi. Early kinetics of haemodynamic signatures were captured during the liver regeneration process (upper panel). Liver to body weight ratio of wild-type male mouse after PHx (sham: *n* = 5, 3 h: *n* = 6, 6 h: *n* = 4, 12 h: *n* = 6, D-1: *n* = 6, D-2: *n* = 6, D-4: *n* = 6 mice; lower panel). **i**–**l** Temporal kinetics of *Klf2*, *Angpt2*, *Wnt2* and *Wnt9b* expression during liver regeneration. Relative RT-qPCR gene expression analysis was performed on MACS-bead sorted LSEC post-PHx. () indicates fold difference. **c**–**g** Data are presented as mean ± s.e.m. **d**–**g** The gene expression was normalised to *Pecam1* and relative fold changes were calculated in comparison to the static control. **h**–**l** Data are presented as mean ± s.d (background area). The gene expression was normalised to *Pecam1* and relative fold changes were calculated in comparison to sham-operated controls (dotted line). **c** Two-tailed unpaired *t*-test was performed. **d**–**g**, **h**–**l** One-way ANOVA followed by Bonferroni test was performed.

To identify zonation-enforcing biophysical parameters in the liver, we surveyed the literature for conditions that affect hepatic metabolic zonation. Intriguingly, pathological conditions that alter hepatic blood flow, such as heart failure, cirrhosis, or portosystemic shunt, were found to result in loss of glutamine synthetase (GS), a strictly pericentrally zonated metabolic enzyme that is tightly regulated by angiocrine Wnt^25,26^. Together with the finding that pericentral EC are generally more stretched and exposed to blood cells of higher velocity^27,28^, we consequently hypothesised that blood flow may be a major determinant of the LSEC transcriptional programme and, hence, a defining biophysical parameter for establishing hepatic zonation. To test this hypothesis, liver samples of mice suffering from congenital hepatic portosystemic shunts^29^, which led to chronically reduced blood flow to the liver, were analysed (Fig. 1a and Supplementary Fig. 1a). Compared to healthy controls, the hepatic zonation pattern of mice with shunts was significantly perturbed, as evidenced by downregulation of GS (Fig. 1b, c)^26^. A similar loss of hepatocytic GS expression was also observed for Wnt-deficient mutant mice^4,9–11,21^, which hinted towards the mechanosensitive expression of vascular Wnt.

To determine whether flow directly affected the expression of vascular Wnt, primary LSEC were isolated from wild-type adult murine livers and exposed to different shear conditions in vitro (Supplementary Fig. 1b). As proof of principle, the expression of shear-responsive endothelial genes *Klf2* and *Angpt2* was assessed^30^. As expected, *Klf2* expression was significantly induced under shear conditions, whereas *Angpt2* expression was repressed under high shear conditions (Fig. 1d, e). In line with our hypothesis, the expression and secretion of bioactive vascular *Wnt2* and *Wnt9b* were induced in a flow-dependent manner under fluidic shear stress (Fig. 1f, g and Supplementary Fig. 1c). To further dissect the mechanism of flow-regulated Wnt regulation, a specialised in vitro fertilisation dish was used to discriminate the laminar shear and oscillatory shear response (Supplementary Fig. 1d). Predictably, oscillatory shear distinctly induced oscillatory shear-sensitive markers, *Icam1* and *Vcam1* (Supplementary Fig. 1e–h), whereas *Klf2* and *Klf4* expressions were more susceptible to laminar shear stress (Supplementary Fig. 1i–l). Both oscillatory shear stress as well as laminar shear stress induced the expression of *Klf2* and *Wnt2* under high shear conditions (Supplementary Fig. 1i–p). The expression of *Wnt9b*, however, remained unchanged under either condition. These results indicated that vascular *Wnt2* expression was directly regulated by oscillatory or laminar shear stress (Supplementary Fig. 1e–p). In parallel, the findings were further corroborated by parallel flow assays (Supplementary Fig. 1q). Upon activation of high laminar flow and high oscillatory flow, a distinct shear response was detected. As expected, expression of *Klf2* and *Klf4* was upregulated at high laminar flow, whereas expression of *Icam1* and *Vcam1* was induced at high oscillatory flow (Supplementary Fig. 1r, s). In line with our previous result, *Wnt2* expression was induced upon high laminar flow and more profoundly at high oscillatory flow conditions (Supplementary Fig. 1t). Hence, *Wnt2* expression was more sensitive towards oscillating shear, confirming that vascular Wnt expression is sensitive towards oscillating shear stress^31,32^. Overall, these results corroborate that the expression of vascular Wnt factors was induced by the haemodynamic response in vitro.

To confirm these findings in vivo, we surgically pursued a model of increased hepatic blood perfusion by employing 2/3rd partial hepatectomy (PHx). This procedure results in the overloading of the regenerating lobe with excessive blood, thereby inducing high shear conditions (Fig. 1h). Consistent with previous reports^33–35^, a transient upregulation of the shear-response genes *Klf2*, *Klf4, Nos3, Gja1* and *Gja4* (Fig. 1i and Supplementary Fig. 2a–d) and a downregulation of growth inhibitory angiocrine factor *Angpt2* (Fig. 1j) was observed during the early inductive phase of liver regeneration^7^. Notably, *Wnt2* and *Wnt9b* expression was transiently increased upon the induction of shear-responsive genes (Fig. 1k, l). In line with these findings, shear-sensitive *Klf2* was upregulated immediately after the shear induction, whereas *Wnt2* expression was induced only 12 h after the high laminar shear in vitro (Supplementary Fig. 2e, f). Concurrently, the transient activation of mechanosensitive genes coincides with a temporal increase in blood perfusion, vessel area, and vessel volume during early stages of PHx^36^. To exclude surgery-related effects, hepatic blood flow was also induced in vivo by treating mice pharmacologically with a vasodilating agent, Riociguat (Supplementary Fig. 2g)^37^. In line with the surgical approach, vasodilation stimulated the haemodynamic response in liver EC and consequently induced vascular Wnt expression (Supplementary Fig. 2h–k) along with a dilated vasculature and upregulated mechanosensitive ITGB1 and VEGFR3 phosphorylation (Supplementary Fig. 2l–q). To further validate these findings, we employed an ex vivo perfusion model that allowed for the direct modulation of hepatic flow (Supplementary Fig. 2r)^23,38^. In line with the previous results, *Klf2*, *Klf4,* and *Wnt2* showed marked upregulation under accelerated flow (Supplementary Fig. 2s–v). Taken together, these data establish that biomechanical activation of hepatic EC triggers vascular Wnt expression.

### Hypoxia and oxidative stress do not induce vascular Wnt expression

Besides blood flow, other biophysical parameters show similar zonation gradients in the liver. For instance, oxygen availability is strictly zonated alongside the porto-central liver axis, with the pericentral region being a highly hypoxic microenvironment^39^. To investigate the effect of hypoxia on LSEC zonation, freshly isolated murine primary LSEC were incubated under hypoxic and atmospheric oxygen conditions in vitro. As expected, *Slc2a1* expression was upregulated in response to the hypoxic challenge (Supplementary Fig. 3a). Hypoxia did not induce pericentrally zonated Wnt factors, but rather seemed to repress *Wnt2* expression, whereas no significant effect was observed for *Wnt9b* (Supplementary Fig. 3b, c). Chemical stabilisation of hypoxia-inducible factor (HIF) using cobalt chloride (CoCl_2_) further validated this finding as *Slc2a1* expression was induced, validating the hypoxic response, whereas *Wnt2* and *Wnt9b* showed markedly reduced expression upon CoCl_2_ treatment (Supplementary Fig. 3d–f). Similarly, exposing mice to seven days of normobaric hypoxic challenge^40^, as shown by marked induction of *Hif1a*, *Epas1,* and *Slc2a1* expression, had no effect on hepatic vascular Wnt expression or hepatic metabolic zonation (Supplementary Fig. 3g–m). Concordantly, induction of acute hypoxic response by short-term inhibition of HIF prolyl-hydroxylase in vivo did not induce vascular Wnt expression nor alterations in pericentral hepatocyte identity (Supplementary Fig. 3n–q). Extending the treatment period to one week did not alter vascular Wnt expression (Supplementary Fig. 3r–u). Taken together, these data excluded a prominent role of hypoxia as a modulator of vascular Wnt and hepatic zonation.

As the cytochrome enzymatic activity of hepatocytes is restricted to the pericentral region^16,39^, we tested whether the resulting reactive oxygen species (ROS) may contribute to the zonated vascular Wnt expression profile. For this purpose, mice were treated with the antioxidant agent, N-acetylcysteine (NAC), to reduce hepatic oxidative stress. Similar to hypoxia, relieving oxidative stress did not affect vascular Wnt expression or metabolic hepatocyte zonation (Supplementary Fig. 3v–y). Collectively, these data indicate that blood flow-mediated haemodynamic factors and not hypoxia or oxidative stress modulate vascular Wnt expression.

### Vascular Wnt factors sustain liver homeostasis and regeneration

**Fig. 2:**
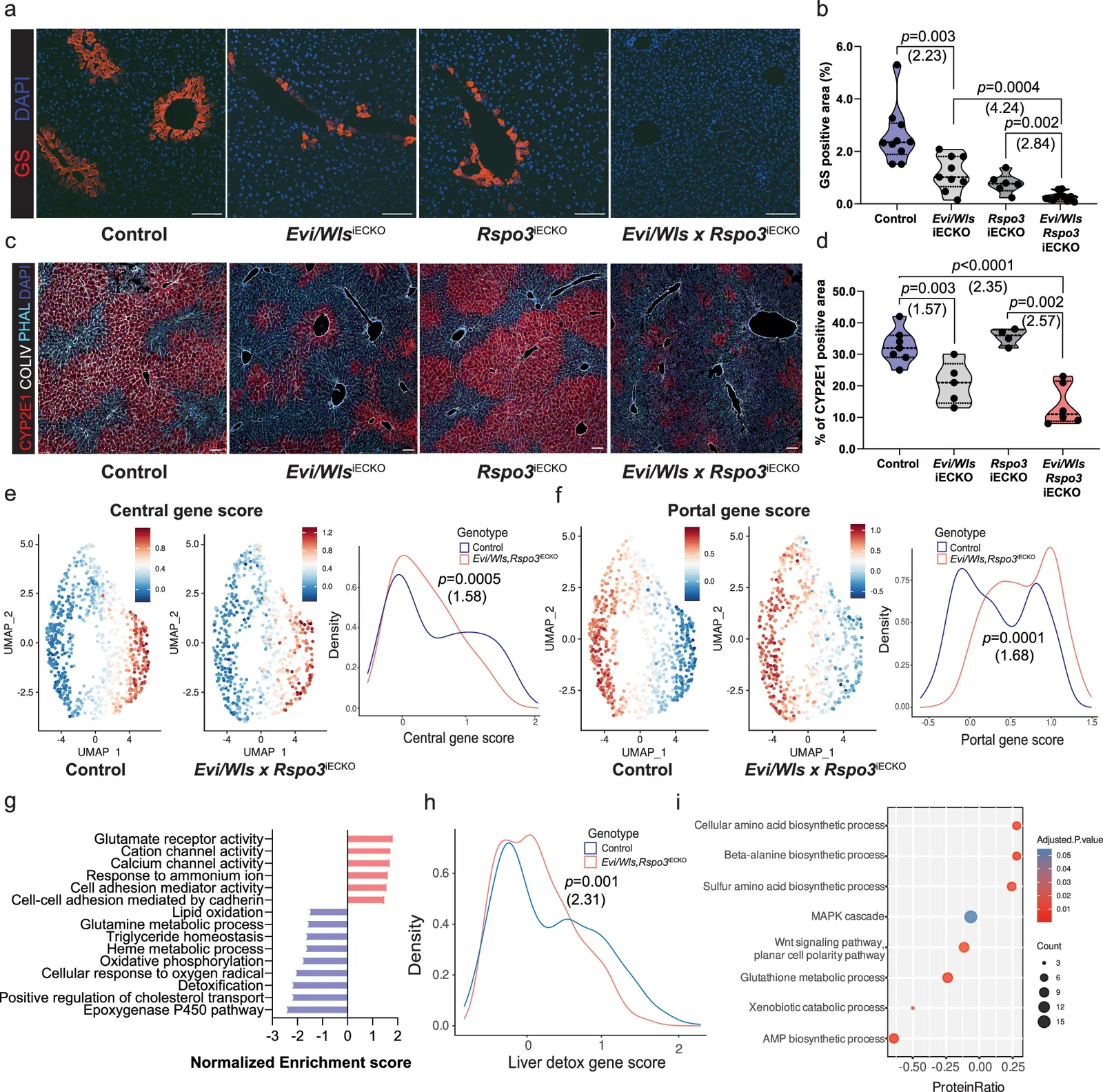
Endothelial-derived Wnt ligands and Rspo3 sustain the spatial division of labour in the liver. **a** Representative immunofluorescence image of GS (red) in the liver. The genotypes are indicated below. Scale bars, 100 μm. **b** Percentage of GS positive area in the whole liver section (control, *n* = 10; Evi/Wls iECKO, *n* = 9; Rspo3 iECKO, *n* = 6; Double-iECKO, *n* = 11 mice). **c** Representative immunofluorescence image of CYP2E1 (red) in the liver. The genotypes are indicated below. Vessels are highlighted by COLIV (grey) staining, cell boundaries are marked by Phalloidin staining (cyan), and cell nuclei are visualised with DAPI (blue). Scale bars, 100 μm. **d** Percentage of CYP2E1 positive area in the whole liver section (control, *n* = 7; Evi/Wls iECKO, *n* = 5; Rspo3 iECKO, *n* = 4; Double-iECKO, *n* = 6 mice). **e** Uniform Manifold Approximation Projection (UMAP) of the total hepatocyte population from control (left panel, *n* = 3) and Double-iECKO mice (middle panel, *n* = 4). Cells are coloured according to central hepatocyte signature gene scores. Log-normalised density plot of the average central hepatocyte gene score calculated for control hepatocytes (blue line) and Double-iECKO hepatocytes (red line; right panel). **f** UMAP of the same dataset, with cells coloured according to their portal hepatocyte gene score (left and middle panel). Log-normalised density plot of the average portal hepatocyte gene score calculated for control (blue line) and Double-iECKO hepatocytes (red line; right panel). **g** Gene set enrichment analysis (GSEA) of the total hepatocyte population comparing Double-iECKO and littermate controls. Differential genes were identified using DESeq2 comparing pseudobulks reflecting the biological replicates across genotypes. A two-tailed Wald test and Benjamini–Hochberg correction were used. Threshold for significance was set to *p* < 0.05 and *q* < 0.1. Normalised enrichment score is shown for each GO term. The red bar shows upregulated, and the blue bar represents downregulated GO terms. **h** Log-normalised density plot of average gene score calculated for the detoxification pathway. **i** Limma-normalised GSEA of proteomics data followed by two-tailed Student’s *t*-test and Benjamini–Hochberg correction comparing central hepatocytes of Double-iECKO (*n* = 3) and control (*n* = 3). Colour refers to the adjusted *p*-value, and dot size represents the number of proteins. +/− sign represents downregulated or upregulated GO, respectively. () indicates fold difference. **a**–**i** Both female and male mice were considered in the study. **b**, **d** Data are presented as mean ± s.e.m. **b**, **d**–**f**, **h** Average gene scores from individual mice were acquired and a two-tailed unpaired *t*-test comparing control and Double-iECKO was performed.

To unravel the physiological consequences of vascular Wnt signalling in sustaining the spatial division of hepatocytes and LSEC, we established an EC-specific inducible knockout mouse model of *Evi/Wls*, a cargo receptor for Wnt ligands, and the Wnt potentiator *Rspo3*, as well as the *Evi/Wls* and *Rspo3* double knockout model (Double-iECKO). Comparing the different mouse strains revealed synergistic effects of Wnt and Rspo3 signalling in maintaining metabolic liver zonation, as Double-iECKO mice exhibited an aggravated loss of GS compared to the single knockouts (Fig. 2a, b). In contrast, the loss of vascular Wnt and Rspo3 showed divergent effects on CYP2E1 expression. Whereas *Evi/Wls* and Double-iECKO mice showed a marked reduction of CYP2E1, *Rspo3*-iECKO mice did not display loss of CYP2E1 (Fig. 2c, d). This may be explained by the overall abundance of vascular Wnt factors, with Wnt2 in particular being more widely expressed throughout the liver compared to angiocrine Rspo3. As Wnt ligands and Rspo3 are short-range acting molecules, the difference in abundance could compensate for the loss of Rspo3 in Rspo3-iECKO mice^41^ and by other cellular sources such as hepatic stellate cells via pericrine Rspo3 signalling^42^.

To assess the functional consequences of the hepatic zonal defect, semi-targeted metabolic screening of whole liver lysates and plasma samples from Double-iECKO and littermate controls was performed. Notably, accumulation of ammonium, potassium, and sugar molecules, including fructose and hexose caused by loss of GS activity and perturbed pericentral glycolysis, respectively, was observed in the liver (Supplementary Fig. 4a–d). Systemically, proline, alanine, oxalic acid, and β-alanine were found to be accumulated, hinting towards periportal dominance with an increased portal hepatocyte-modulated amino acid catabolism (Supplementary Fig. 4e–h). In parallel, Wnt gain of function experiments showed a reversed phenotype with reduced amino acid abundance confirming Wnt-dependent metabolic functions^43^.

Long-term tracing of Double-iECKO mice revealed that loss of Wnt signalling was not compensated for, as evidenced by the persistent loss of GS that was additionally accompanied by an overall reduction in liver mass (Supplementary Fig. 4i–k). It was recently reported that hepatocyte progenitor cells exist across the porto-central liver axis and are marked by distinct gene expression profiles depending on their spatial location^44–50^. To elucidate which progenitor pool was affected and consequently led to the reduced liver mass in Wnt-deficient mice, the gene expression of hepatic progenitor markers was assessed. Interestingly, gene expression of the periportal progenitor marker genes *Sox9* and *Mfsd2a* (Supplementary Fig. 4l, m)^44,45^, and the midzonal progenitor marker genes *Igfbp2* and *Hamp2*^46^ were not altered between Double-iECKO and control mice (Supplementary Fig. 4n, o), whereas pericentral progenitor marker genes *Axin2* and *Lrg5*^21,51^ showed a downregulation upon vascular Wnt loss (Supplementary Fig. 4p, q). This suggested that vascular Wnt is crucial in maintaining the pericentral progenitor niche and that pericentral progenitor hepatocytes are essential for the maintenance of liver mass.

As angiocrine Wnt was established to exert crucial maintenance functions under homeostatic conditions, we performed 2/3rd PHx on Double-iECKO and littermate control mice to test the importance of angiocrine Wnt during liver regeneration. In line with the previous findings^9,11^, Double-iECKO mice showed impeded liver regeneration characterised by a reduced liver-to-body weight ratio and fewer proliferating hepatocytes during the inductive phase of liver regeneration (Supplementary Fig. 5a–d). Moreover, a similar reduction of liver mass was observed during the angiogenic phase of liver regeneration (Supplementary Fig. 5e), indicating that hepatocyte proliferation was not simply attenuated but reduced in general (Supplementary Fig. 5f–h). This reduced liver regeneration capacity was mainly caused by the loss of pericentral hepatic progenitors (Supplementary Fig. 5i–n). To further corroborate this result, the number of proliferating hepatocytes was quantified in a zone-specific manner using GS expression and bile ducts as anchors for each zone (Supplementary Fig. 5o). In line with a previous report^42^, midzonal hepatocytes dictated hepatic regeneration. Consequently, fewer proliferating hepatocytes were observed in midzonal and central zones upon the loss of angiocrine Wnt factors, whereas the portal zone remained unaffected, suggesting Wnt-dependency of midzonal and central hepatocytes during liver regeneration (Supplementary Fig. 5p–r). Overall, these data denote that vascular Wnt factors serve as the guardians of liver zonation, size, and function.

### Vascular Wnt factors orchestrate the spatial division of labour in the liver

To molecularly define the mechanisms by which vascular Wnt signalling is maintaining liver homeostasis, we performed scRNA-seq using the CEL-seq2 protocol^52^ on FACS-sorted hepatocytes and LSEC of Double-iECKO and littermate control mice (Supplementary Fig. 6a, b). The samples with successful gene deletion, as evidenced by immunofluorescence staining of GS, were further processed (Supplementary Fig. 6c). To validate the genetic recombination, pseudobulk differential gene expression (DEG) analyses were performed on hepatocyte and LSEC datasets. In line with the loss of GS, *Glul* expression was downregulated in hepatocytes (Supplementary Data 1 and Supplementary Fig. 6d). Additionally, targeted genes, including *Rspo3* and *Wls* expression, were downregulated in LSEC dataset, confirming the robust gene deletion (Supplementary Data 1 and Supplementary Fig. 6e). Consequently, the scRNA-seq data were further processed and the data were visualised in a Uniform Manifold Approximation and Projection (UMAP). The hepatocyte and LSEC data clustered into three subgroups, which based on previously established marker gene sets, could be assigned to the portal, midzonal, or central area of the liver lobule (Supplementary Fig. 6f–i).

Next, the scRNA-seq data of LSEC and hepatocytes from Double-iECKO and control mice were spatially resolved by aligning the transcriptomes alongside a pseudo porto-central axis^8,17,22,53^ and the expression of previously published zonation marker genes was assessed^16,17,22^. Loss of vascular Wnt did not affect the zonal expression pattern of the pericentral hepatocyte marker *Nt5e* or the periportal hepatocyte marker *Cdh1*^53^. Moreover, the previously reported gradual expression of *Kit*^8,17,22^ was maintained in LSEC of Double-iECKO mice (Supplementary Fig. 6j–l). To orthogonally validate the sequencing data, hepatocytes and LSEC from Double-iECKO and control mice were spatially sorted as bulk samples and proteomic analysis was performed. To this end, hepatocytes were discriminated on the basis of CD73 and CD324 expression in FACS and four equally sized fractions were sorted, reflecting the portal, periportal, pericentral, and central hepatocytes (Supplementary Fig. 6m). Similarly, four equally sized fractions of the LSEC samples were sorted according to c-Kit expression, with c-Kit high cells being located close to the central vein and c-Kit low cells near the portal vein (Supplementary Fig. 6n).

In line with previous studies^9,12,4,10,21^, the data corroborated that the metabolic fate of hepatocytes was strongly dependent on vascular Wnt factors. In general, vascular Wnt factors were dispensable for midzonal hepatocyte marker genes (Supplementary Fig. 6o). In parallel, hepatocytes isolated from Double-iECKO mice showed a loss of the pericentral-like hepatocyte gene signature (Fig. 2e, Supplementary Fig. 7a, b and Supplementary Data 1 and 2), while gaining periportal-like hepatocyte identity (Fig. 2f and Supplementary Fig. 7c, d), which was also observed at the proteome level (Supplementary Fig. 7b, d and Supplementary Data 3). Gene set enrichment analysis (GSEA) on DEG of Double-iECKO and littermate control hepatocyte pseudobulks reflecting the biological replicates highlighted perturbed metabolic functions, such as detoxification and lipid metabolism, in Wnt-deficient mice (Fig. 2g, h and Supplementary Fig. 7e, f). To further examine the functional role of vascular Wnt in a spatial context, the total hepatocyte population was split into three different subsets (Supplementary Fig. 6f, h). Subsequent GSEA on DEG comparing the Double-iECKO and control spatial pseudobulks further substantiated the loss of pericentral and a shift towards periportal hepatocyte identity in Wnt-deficient mice.

In the periportal zone, GO terms related to pericentral hepatocyte functions including lipogenesis and lipid phosphorylation were further downregulated in Double-iECKO pseudobulks, whereas portal hepatocyte functions including gluconeogenesis were found to be enriched (Supplementary Fig. 7g). This shift was also observed for midzonal hepatocytes, which displayed increased periportal-specific amino acid catabolism and reduced pericentral xenobiotic functions (Supplementary Fig. 7h), as well as for pericentral hepatocytes, which showed strong periportal hepatocyte-like features, while losing their pericentral identity (Supplementary Fig. 7i). Validating the transcriptomic approach, GSEA on differentially expressed proteins comparing portal and central hepatocytes from control mice similarly revealed the central bias of xenobiotic catabolic processes and the portal bias of urea cycle (Supplementary Fig. 7j).

Next, central hepatocytes of Double-iECKO and control mice were compared. In line with the transcriptomic data, loss of vascular Wnt was found to induce a metabolic shift as indicated by the reduction in xenobiotic functions and the acquisition of amino acid catabolic functions in mutant central hepatocytes (Fig. 2i). This is in line with previous work that highlighted a vascular Wnt-regulated hepatic xenobiotic core in toxication studies using carbon tetrachloride and acetaminophen^10,11^. Collectively, these data indicate that angiocrine Wnt signalling does not only shape the pericentral hepatocyte niche but is crucial in maintaining overall hepatocyte function and, hence, hepatic performance.

### Vascular Wnt factors shape LSEC function in an autocrine manner

**Fig. 3:**
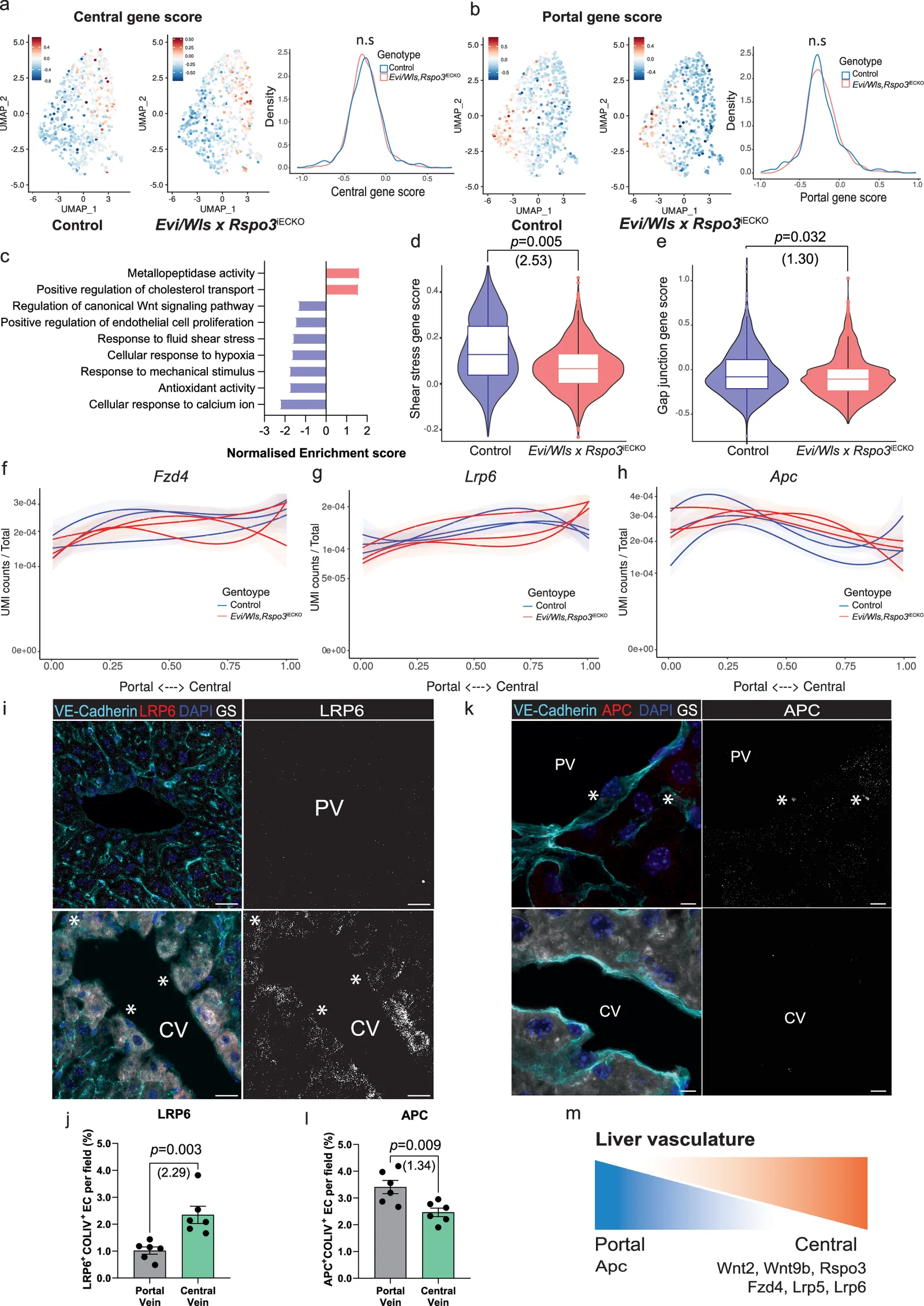
Molecular atlas of LSEC reveals spatial division of Wnt responsiveness and consequent autocrine Wnt function. **a** UMAP of total LSEC population from control (left panel, *n* = 3) and Double-iECKO mice (middle panel, *n* = 3). Cells are coloured by central LSEC signature gene scores. Log-normalised density plot of average gene scores calculated for central LSEC signatures (right panel). **b** UMAP of the same data with cells coloured according to portal LSEC gene score. Log-normalised density plot of average gene score calculated for portal LSEC (right panel). **c** GSEA of central vein EC population comparing Double-iECKO and littermate control. Differential genes were identified using DESeq2, comparing pseudobulks reflecting the biological replicates of the different genotypes. Wald test and Benjamini–Hochberg were used. Threshold for significance was set to *p* < 0.05 and *q* < 0.4. Normalised enrichment score is shown for each GO term. The red bar shows upregulated, and the blue bar represents downregulated GO. **d** Violin plot of average gene score calculated for shear stress associated genes, and **e** for gap junction molecules comparing control (*n* = 3) from Double-iECKO mice (*n* = 3). For violin plots, the horizontal line represents the median value, the lower and upper quartiles mark the 25th and 75th percentiles, and the whiskers indicate the minimum and maximum values. **f**–**h** Spatial expression of Wnt receptors, *Fzd4* and *Lrp6*, and Wnt suppressor *Apc*. Zonation score was calculated based on the previously identified reference genes. Each line represents one biological replicate. Colour indicates the genotype. Shadowed area indicates s.e.m. **i** Representative immunofluorescent image of LRP6 (red) co-stained with the hepatic zonal marker GS (grey) and VE-Cadherin (cyan) for vasculature (left panel). Scale bar shows 20 µm. The asterisk indicates the VE-Cadherin and LRP6 double-positive area. LRP6 expression was further highlighted in grey (right panel). **j** Percentage of COLIV and LRP6 double-positive area in the liver lobule (*n* = 6). **k** Representative immunofluorescent image of APC (red) co-stained with hepatic zonal marker GS (grey) and VE-Cadherin (cyan) for vasculature (left panel). Scale bar shows 10 µm. The asterisk indicates the VE-Cadherin and APC double-positive area. APC expression was further highlighted in grey (right panel). **l** Percentage of COLIV and APC double positive area in the liver lobule (*n* = 6). **m** Model of vascular Wnt zonation. CV central vein, PV portal vein. () indicates fold difference. a–**l** Both female and male mice were incorporated in the study. **j**, **l** Data are presented as mean ± s.e.m. **a**, **b**, **d**, **e** Average gene scores from individual mice were computed and subjected to statistical analysis. **a**, **b**, **d**, **e**, **j**, **l** Two-tailed unpaired *t*-test was performed.

Analyses of the LSEC dataset revealed that the overall LSEC zonation pattern^4,17^ was maintained in Double-iECKO mice (Fig. 3a, b and Supplementary Fig. 8a–d); yet, loss of vascular Wnt led to functional perturbations. Double-iECKO LSEC showed a reduction in canonical Wnt signalling, metabolic programmes, such as nitric oxide (NO) and ROS metabolism, as well as vasodilation (Supplementary Fig. 8e). Indeed, liver tissues from Double-iECKO mice showed less eNOS expression compared to their control (Supplementary Fig. 8f, g). Similar to the hepatocyte dataset, LSEC were split into three subgroups based on the expression of zonal identifier genes reflecting portal, midzonal and central LSEC (Supplementary Fig. 6g, i). Zonal pseudobulks reflecting the biological replicates were then compared between Double-iECKO and control samples and GSEA was performed. Interestingly, loss of Wnt led to the disturbed regulation of blood pressure, as well as NO metabolism in portal venous and midzonal LSEC (Supplementary Fig. 8h, i), whereas pericentral LSEC displayed loss of canonical Wnt signalling and a mitigated response to various milieu factors including shear stress, hypoxia, antioxidation, mechanotransduction and calcium ions (Fig. 3c).

We further validated the data by calculating the average gene scores for genes which are associated with shear stress and gap junctions, and found all genesets to be downregulated in LSEC upon loss of Wnt (Fig. 3d, e). To orthogonally validate the data, GSEA was performed on differentially expressed proteins from the central vein LSEC subpopulation comparing Double-iECKO and littermate control mice. In line with the transcriptomic approach, the analysis revealed a perturbed calcium response in mutant LSEC (Supplementary Fig. 8j). Collectively, the data revealed the existence of an autocrine vascular Wnt function in LSEC, supporting the view of pericentral hepatocytes not being the main vascular Wnt-addicted hepatic cell type.

To further investigate the autocrine vascular Wnt function, the zonal expression pattern of Wnt receptors and the Wnt-processing machinery in the hepatic vasculature were assessed. The expression of Wnt signalling receptors *Lrp6* and *Fzd4* was biased towards the central vein, whereas *Apc*^54^, a Wnt suppressor, was enriched at the portal vein (Fig. 3f–h). Immunostaining of LRP6, FZD4 and APC confirmed that LRP6 protein expression was zonated towards the central vein, whereas FZD4 was evenly expressed across the liver axis (Fig. 3i, j and Supplementary Fig. 8k, l). APC was preferably localised around the portal vein (Fig. 3k, l), suggesting that pericentral LSEC were poised for canonical Wnt signalling, whereas periportal LSEC were sealed through the lack of receptor expression and the default suppression of the pathway.

To further validate our findings, we performed comprehensive analyses on publicly available data and confirmed the vascular Wnt zonation^55^ (Supplementary Fig. 8m). As *Lrp6* and *Fzd4* showed a similar zonation pattern to vascular Wnt factors, we tested whether their expression was also regulated by flow-induced shear stress. Applying shear stress to freshly isolated primary LSEC markedly induced the expression of *Lrp6* and *Fzd4*, pointing towards a similar gene expression regulation of Wnt ligands and Wnt receptors in LSEC in vitro (Supplementary Fig. 8n, o). Taken together, these data suggest an autocrine Wnt signature within the hepatic endothelium whose activity is biased towards the central vein (Fig. 3m).

### Vascular Wnt factors fine-tune angiocrine gene signatures and LSEC morphology

**Fig. 4:**
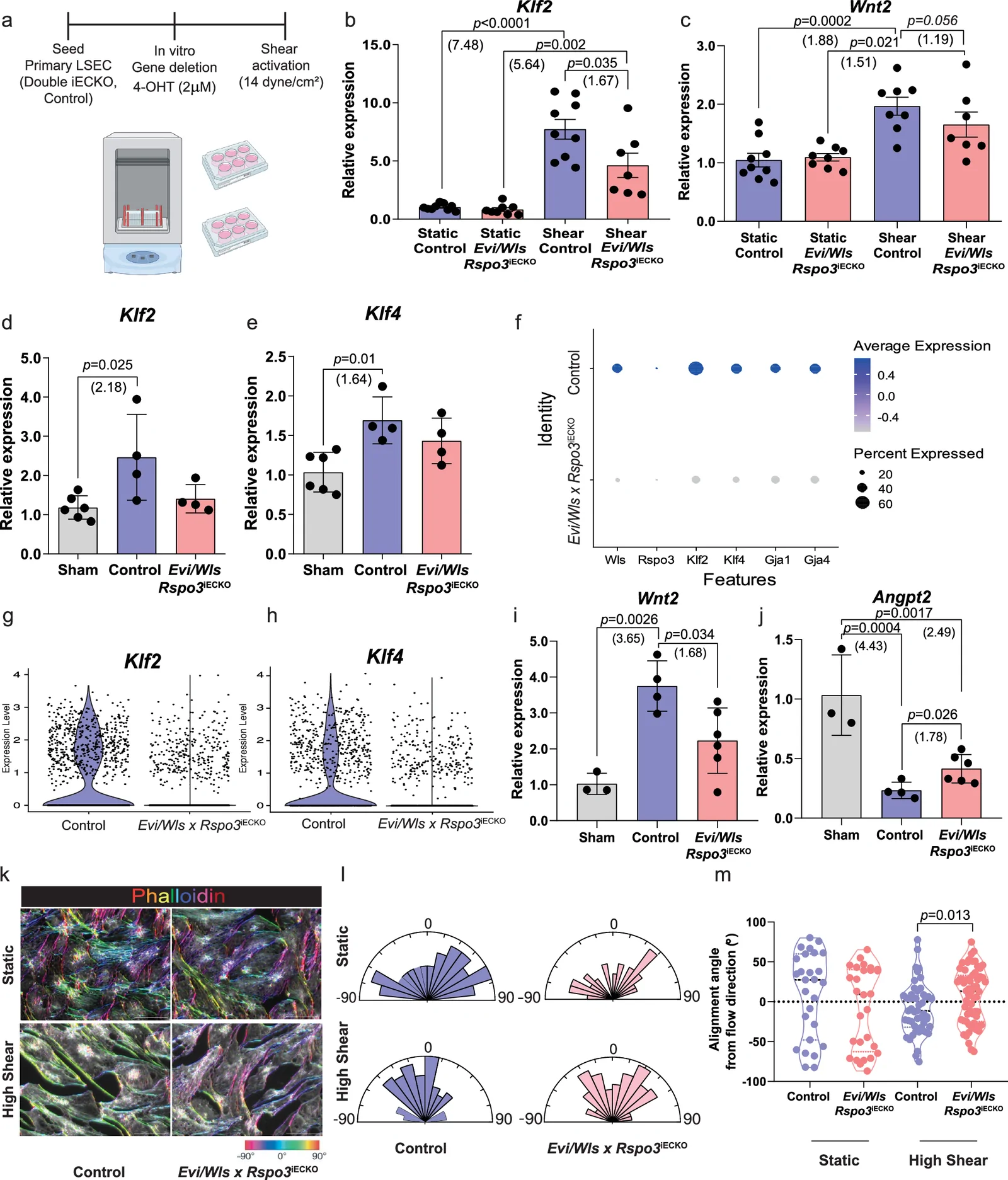
Vascular Wnt factors coordinate LSEC orientation and angiocrine profiles. **a** Experimental scheme of in vitro shear stress model. Primary LSEC were isolated via liver perfusion and MACS- bead sorting. After seeding in 6-well plates, genetic recombination was induced by administering 2 μM of hydroxy-tamoxifen. LSEC were then applied to either static (0) or high shear conditions (14 dynes/cm^2^). Created in BioRender. Lee, KH. (2026) https://BioRender.com/dtxlnfw. **b** Relative gene expression of shear sensing *Klf2* and **c** *Wnt2* in control and Double-iECKO LSEC cultured under different shear conditions (static control, *n* = 9 mice; shear control, *n* = 8 mice; static Double-iECKO, *n* = 8 mice, shear Double-iECKO, *n* = 7 mice). **d** Relative gene expression of shear sensing *Klf2* and **e** *Klf4* 6 h post 1/3rd partial hepatectomy (PHx) (sham, *n* = 6 mice; control, *n* = 4 mice; Double-iECKO, *n* = 4 mice) from MACS-bead sorted LSEC. **f** Dot plot of the total LSEC dataset comparing the control to the mutant samples. Gene expression of *Wls, Rspo3, Klf2, Klf4, Gja1* and *Gja4* were visualized. Average gene expression, in z-score, is shown in the colour code and the dot size illustrates the percentage of EC that express the target genes. Violin plot from the total LSEC scRNA-seq dataset comparing the control to the mutant samples; **g** *Klf2*, **h** *Klf4* mRNA expression. **i** Relative gene expression of angiocrine factors *Wnt2* and **j** *Angpt2* 24 h post 2/3rd PHx (sham, *n* = 3 mice; control, *n* = 4 mice; Double-iECKO, *n* = 6 mice) from MACS-bead sorted LSEC. **k** Phalloidin staining with subsequent in silico segmentation was performed to visualise shear stress fibres comparing static and high shear conditions of primary Double-iECKO and control LSEC. Colour code represents fibre orientation relative to the shear axis (0°). Scale bars, 5 µm (static control, *n* = 9 mice; shear control, *n* = 8 mice; static Double-iECKO, *n* = 8 mice, shear Double-iECKO, *n* = 7 mice). **l** Rose plot represents cell alignment angle normalised to the direction of shear (0°). **m** Quantification of cell alignment angle relative to the direction of the shear (0°). Each dot represents a single image. () indicates fold difference. **b**–**m** Both female and male mice were incorporated in the study. **b**–**e**, **i**, **j** Gene expression was normalised to *Pecam1*. **d**, **e**, **i**, **j**, the relative fold change was calculated with respect to sham-operated mice. **b**, **c** Data are presented as mean ± s.e.m. **d**, **e**, **i**, **j** Data are presented as mean ± s.d. **d**, **e**, **i**, **j** One-way ANOVA followed by Bonferroni test was performed. **b**, **c**, **j**, **m** Two-tailed unpaired *t*-test was performed.

To examine the functional consequences of the autocrine Wnt factors, we applied shear stress to primary LSEC isolated from Double-iECKO and littermate control mice (Fig. 4a). For this purpose, freshly isolated LSEC were cultured and genetic recombination was induced in vitro (Supplementary Fig. 9a, b). Upon shear activation, Double-iECKO LSEC showed less induction of the shear response genes *Klf2* and *Klf4* (Fig. 4b and Supplementary Fig. 9c). Additionally, *Wnt2* induction was similarly impeded (Fig. 4c). To validate this finding in vivo, 1/3rd PHx was performed on Double-iECKO and control mice and the early haemodynamic response of LSEC was traced (Supplementary Fig. 9d). In line with the in vitro data, Double-iECKO LSEC showed less induction of shear response genes compared to sham-operated mice (Fig. 4d, e). Similarly, pseudobulk DGE analysis of total LSEC showed reduced gene expression of *Klf2* and *Klf4* (Supplementary Data 1 and Fig. 4f–h).

Next, Double-iECKO and control mice were subjected to 2/3rd PHx to assess the instructive angiocrine expression profile, which reaches its peak 24 h after surgery (Supplementary Fig. 9e). *Wnt2* induction was impaired in mutant LSEC (Fig. 4i). Furthermore, *Angpt2* expression was dysregulated during the induction phase of liver regeneration (Fig. 4j). In line with this, *Angpt2* expression was retained in isolated Double-iECKO primary LSEC upon high shear activation in vitro (Supplementary Fig. 9f).

We next sought to decipher the macroscopic impact of the disturbed molecular shear sensors in Double-iECKO LSEC and analysed the orientation of F-actin filaments and traced the morphological changes upon shear stimulation (Fig. 4k). Typically, under laminar flow, EC, including LSEC align in direction to the flow by rearranging their cytoskeleton. While control LSEC were able to align in response to flow, Double-iECKO LSEC displayed poor alignment (Fig. 4l, m), which was further confirmed by a pump-based flow experiment (Supplementary Fig. 9g, h). To assess whether Wnt-deficient LSEC in vivo showed similar morphological abnormalities, high-resolution electron microscopy was used to examine the morphological features of hepatic EC in liver sections of Double-iECKO and littermate control mice. Strikingly, mutant central venous EC showed structural defects as evidenced by thickening and detachment of the basement membrane, while overall mimicking pseudo-capillarisation through the loss of fenestration (Supplementary Fig. 9i). Intriguingly, central vein EC from Double-iECKO were found to be significantly smaller compared to their control and the lumen size of the hepatic vasculature was reduced in Double-iECKO mice, which implicates autocrine Wnt functions in shaping LSEC size (Supplementary Fig. 9j–r). In summary, Wnt signalling was found to be crucial for LSEC function by regulating cell morphology and fine-tuning the expression dynamics of angiocrine gene signatures.

### Endothelial autocrine Wnt controls gap junction expression

**Fig. 5:**
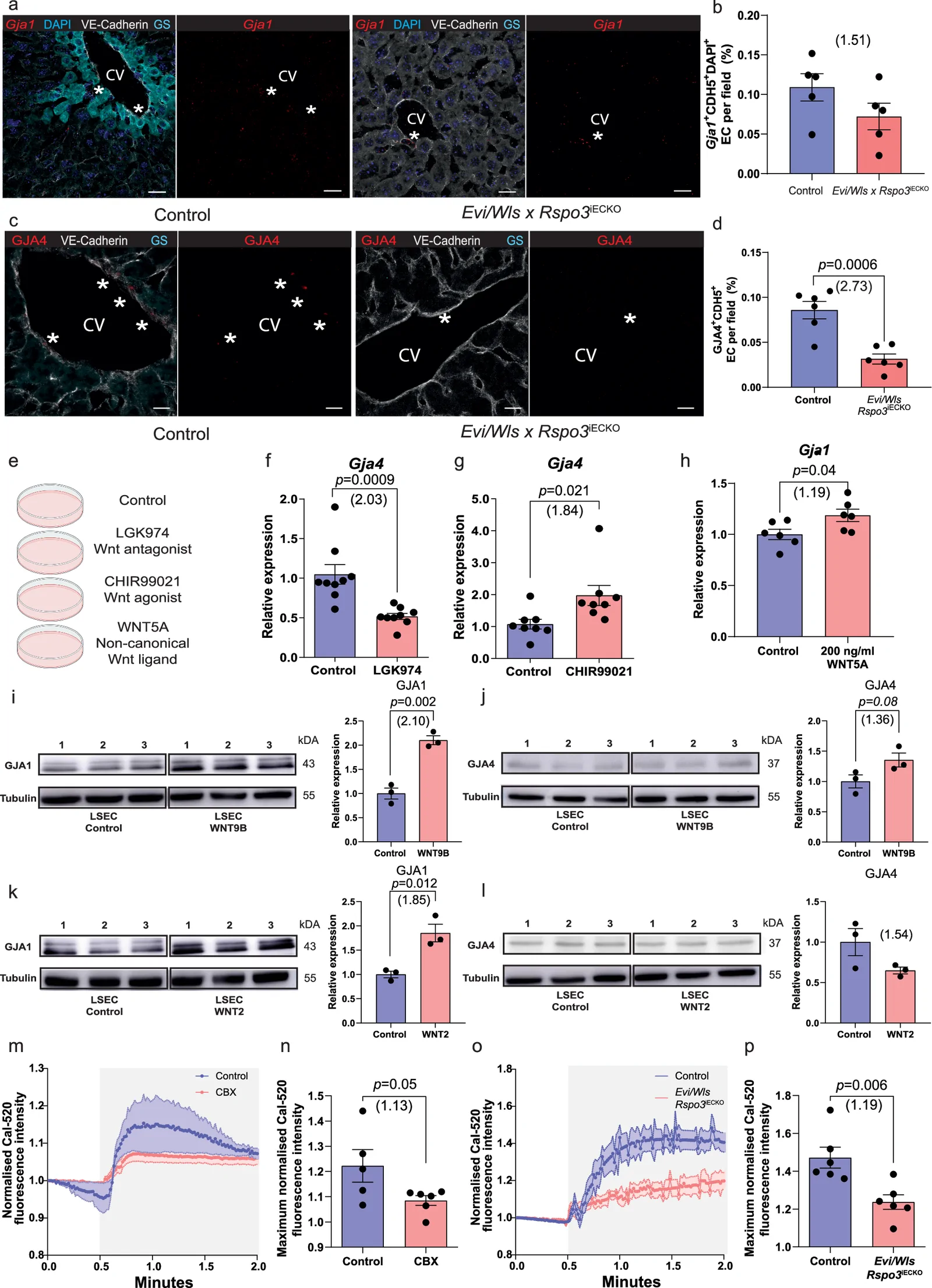
Vascular Wnt factors fine-tune gap junction expression. **a** Representative in situ hybridisation image of *Gja1* (red) on DAPI (blue) co-stained with hepatic zonal marker GS (cyan) and VE-Cadherin (white) for vasculature at the central vein. *Gja1* expression was further highlighted in red (right panel). *Gja1* expression between the mutant (*n* = 5) and littermate control (*n* = 5) was compared. Asterisks indicate the *Gja1*, VE-Cadherin and DAPI triple-positive area. **b** Corresponding quantification of *Gja1*, VE-Cadherin and DAPI triple-positive area on liver sections of control mice and mutant mice. **c** Representative immunofluorescence image of GJA4 (red) co-stained with hepatic zonal marker GS (cyan) and VE-Cadherin (white) for vasculature at the central vein. GJA4 expression was further highlighted in red (right panel). GJA4 expression between the mutant (*n* = 6) and littermate control (*n* = 6) was compared. Asterisks indicate the GJA4 and VE-Cadherin double-positive area. **d** Corresponding quantification of GJA4 and VE-Cadherin double-positive area on liver sections of control mice and mutant mice. **e** Experimental scheme for pharmacological manipulation of Wnt signalling pathway. Created in BioRender. Lee, KH. (2026) https://BioRender.com/pahnmbj. **f** Relative gene expression of gap junctional molecule *Gja4* in wild-type primary LSEC after LGK974 (*n* = 9) or **g** CHIR99021 treatment (*n* = 8). **h** Relative gene expression of gap junctional molecule *Gja1* in wild-type primary LSEC after stimulation with different WNT5A concentrations (*n* = 6). **i** Immunoblotting of GJA1 and Tubulin (left) and quantification (right) and **j** immunoblotting of GJA4 and Tubulin (left) and quantification (right) comparing WNT9B treated primary LSEC (*n* = 3) and its control (*n* = 3). **k** Immunoblotting of GJA1 and Tubulin (left) and quantification (right) and **l** immunoblotting of GJA4 and Tubulin (left) and quantification (right) of comparing WNT2 treated (*n* = 3) and its control (*n* = 3). **m** Live cell calcium imaging of wild-type primary LSEC treated with either water (*n* = 5) or gap junction channel blocker Carbenoxolone (CBX, *n* = 6), positive for calcium indicator, Cal-520, after calcium administration (grey background). The fluorescence intensity was normalised to the resting state. **n** Maximum normalised fluorescence intensity ratio of (**m**). **o** Live cell calcium imaging of Double-iECKO (*n* = 6) and control LSEC (*n* = 6) positive for calcium indicator, Cal-520, after calcium administration (grey background). The fluorescence intensity was normalised to the resting state. **p** Maximum normalised fluorescence intensity ratio of (**o**). () indicates fold difference. **a**, **c** Scale bar shows 10 µm. CV indicates the central vein area. **a**–**d**, **f**–**p** Both female and male mice were incorporated in the study. **h**–**l** Data are presented as mean ± s.d. **b**, **d**, **f**, **g**, **m**–**p** Data are presented as mean ± s.e.m. **b**, **d**, **f**, **g**, **i**–**l**, **n**, **p** Two-tailed unpaired *t*-test was performed. **h** One-way ANOVA followed by the Bonferroni test was performed.

During the process of mechanotransduction, flow stimulates mechanoreceptors and induces mechanosensing ion channel activity, thereby mediating the calcium-directed downstream pathway^56^. The single-cell data revealed connexin 37 (Cx37, *Gja4*) and connexin 43 (Cx43, *Gja1*) as highly Wnt-dependent effector genes that modulate ion current and calcium influx^57^. Additionally, several genes, including *Arhgap29, Il1a, Nfkbiz, Cacng2, Psmd2 and Slco3a1*, were identified as Wnt-regulated genes (Supplementary Data 2). Loss of vascular Wnt led to a significant reduction of Cx37 and Cx43 expression in vivo (Supplementary Data 1, Fig. 4f and Supplementary Fig. 10a–f) and in vitro (Supplementary Fig. 10g, h). To further dissect the zone-dependent function of vascular Wnt signalling, spatial coordination of gap junctional molecules was examined. In situ hybridisation of *Gja1* revealed a reduction of mRNA expression at the central vein upon vascular Wnt loss, whereas *Gja1* expression did not differ in the portal vein (Fig. 5a, b and Supplementary Fig. 10i, j). Similarly, GJA4 protein expression was markedly downregulated in the central vein of the vascular Wnt-deficient liver, whereas GJA4 abundance was unaffected in the portal vein (Fig. 5c, d and Supplementary Fig. 10k, l). Remarkably, similar to vascular Wnt factors, the expression of both gap junction molecules showed regulation by flow-induced shear stress and a spatial expression pattern with central bias (Supplementary Fig. 2c, d and Supplementary Fig. 10c, d). Overall, these results show that vascular Wnt is short-ranged and promiscuous, modulating spatially distinct gap junctional molecule expression in an autocrine manner.

To demonstrate the vascular Wnt control of gap junction expression, freshly isolated primary LSEC were either treated with Wnt antagonist (LGK974) or Wnt agonist (CHIR99021) in vitro. A reduction of *Axin2* expression under antagonistic and induction of *Axin2* under agonistic conditions validated the experimental setup (Fig. 5e and Supplementary Fig. 11a, b). As hypothesised, *Gja4* expression was significantly decreased upon LGK974 treatment (Fig. 5f), whereas agonistic treatment with CHIR99021 induced *Gja4* expression (Fig. 5g). In contrast, *Gja1* was not regulated by canonical Wnt signalling, which led us to explore the role of non-canonical Wnt ligands in regulating gap junction expression (Supplementary Fig. 11c, d). Strikingly, WNT5A, a prototypic non-canonical Wnt ligand, triggered *Gja1* and *Gja4* expression (Fig. 5h and Supplementary Fig. 11e). Likewise, recombinant WNT2 and WNT9B, mimicking the pericentral Wnt-milieu of LSECs, induced protein levels of GJA1, whereas GJA4 levels did not change significantly (Fig. 5i–l). Furthermore, consistent with the transcriptomic data, LGK974-directed Wnt inhibition led to downregulation of GJA4 (Supplementary Fig. 11f, g). Concurrently, CHIR99021-derived Wnt activation induced GJA4 expression, whereas GJA1 expression was only regulated upon Wnt activation (Supplementary Fig. 11h–k). This indicated the fine tuning of gap junctional expression via both, the canonical and non-canonical Wnt signalling pathways in the liver and further provided evidence for an autocrine vascular Wnt function in LSEC that is regulating the gap junction expression.

We next examined the functional relevance of junctional molecules by performing live cell calcium imaging on primary LSEC from Double-iECKO mice and littermate controls in vitro. Treating primary LSEC with the gap junction blocker carbenoxolone inhibited channel activity, as evidenced by reduced calcium flux, thereby confirming the regulatory properties of gap junctional molecules in LSEC (Fig. 5m, n). Calcium tracing in mutant LSEC revealed perturbed channel function and calcium signalling in both steady and shear conditions (Fig. 5o, p and Supplementary Fig. 11l, m), which could be mimicked by Wnt antagonistic treatment of wild-type LSEC with LGK974 (Supplementary Fig. 11n, o). Overall, these data demonstrated perturbed gap junction-mediated calcium propagation upon vascular Wnt loss in vitro, highlighting vascular Wnt control of gap junctional molecules.

## Discussion

Our understanding of the spatial division of labour in the liver has remained descriptive. Here, we sought to elucidate the driving force behind hepatic angiodiversity that directs much of the spatial gene expression pattern observed in the liver. At the molecular level, the transcription factors *Gata4* and *c-Maf* have emerged as crucial players in establishing liver sinusoidal EC identity^13,14^, whereas Notch and TGF-β signalling were identified as modulators of hepatic angiodiversity^58,59^. Hepatic EC specification is an active process involving mechanosignalling. Recently, Heg1 and Alk1, critical components of vascular mechanotransduction, have been identified as key regulators of hepatic vascular patterning and hepatic zonation via vascular Wnt factors^59,60^. Yet, these factors do not resolve the full extent of liver endothelial zonation and the longstanding debate over the major determinant of vascular zonation. In this regard, oxygen availability is considered a major driver of the spatial division of labour in the liver, as the central venous region is hypoxic^39^. However, inducing hypoxia in vitro and in vivo did not impact vascular Wnt expression and hepatic zonation, suggesting that oxygen availability is not the crucial biophysical cue that orchestrates liver zonation. In line with this, we were able to corroborate the vascular mechanotransduction concept of liver function in unique patient samples that we were able to retrieve from the pathology archive. The first case report was a patient with liver ischemia. Immunostaining analyses of liver samples from this patient did not reveal any perturbation in the hepatic GS zonation pattern. In contrast, a second case report, a patient with an anatomical heart defect and, hence, presumably altered systemic blood flow, displayed loss of pericentral GS expression in the liver. Intriguingly, a third case report, a patient with hepatic hemangiomatosis, showed loss of GS in tissue areas adjacent to the tumour where poor perfusion is expected, whereas the zonated GS expression was retained in the periphery (Supplementary Fig. 12a–c). Remarkably, the pericentral hepatic region has been shown to be a hotspot of spatial disorganisation in desmoplastic liver^61^. Similarly, a significant reduction in GS expressing pericentral hepatocytes was observed in cirrhotic patient samples, which was associated with sinusoidal dysregulation and a regressed central vein network^62^. In conclusion, albeit being observational case reports, these analytical studies of patient samples very much strengthen the conclusion that vascular Wnt expression is biomechanically activated and thereby controlling zonated liver functions.

EC are highly responsive to mechanical stretching and blood flow-mediated shear stress, resulting in morphological changes and the expression of angiocrine factors^23,58,63,64^. Pericentral LSEC are exposed to bidirectional blood cells of higher velocity and display a more stretched morphology, leading to the establishment of a pericentral niche that is characterised by high shear conditions^27,28^. At the molecular level, the zonated shear response in the hepatic vasculature may be inferred by the finding that the phosphorylation-dependent signalling activity of ITGB1 and VEGFR3, two shear responsive EC surface receptors, was biased towards the central vein, whereas their expression was not regulated spatially^22^. This finding was further validated by the proximity ligation assays, highlighting increased phosphorylation of ITGB1 and VEGFR3 towards the central zone (Supplementary Fig. 13a–d). Flow-dependent mechanical stimulation of hepatic EC induced vascular Wnt factors. In line with this notion, hepatic Wnt signalling was recently shown to be regulated rhythmically and culminated when hepatic blood flow is at its peak^65,66^. Similarly, mechano-dependent Wnt expression was also observed in ECs stimulated by oscillatory shear and for EC in the valves of the heart, pointing towards a more general mechanism^24^. In parallel, a recent spatiotemporal atlas of murine liver regeneration further supported the transient activation of haemodynamic response genes preceding the Wnt secretion^67^. Likewise, the phenotypic switch of liver EC was achieved through a flow-perturbing partial ligation of the inferior vena cava^68^.

The functional experiments in this study revealed that shear-induced angiocrine Wnt-expression was indispensable for maintaining a Wnt-addicted pericentral and midzonal hepatocyte progenitor niche^42^, which was crucial for sustaining liver mass and hepatocyte proliferation during regeneration. This finding was corroborated by previous work, showing that hepatocyte-specific deletion of Wnt receptors and modulators similarly impaired hepatic function^54,69,70^. Besides maintaining the pericentral hepatocyte niche, these Wnt factors are crucial for transdifferentiation of cholangiocytes into hepatocytes and, thus, are indispensable for developing, maintaining and restoring liver mass^71^.

Interestingly, hepatocytic Wnt- and R-spondin receptor expression is biased towards the central vein, whereas the expression of APC, a Wnt suppressor, is enriched at the portal vein^54^, thereby reinforcing zonated Wnt signalling. These zonated Wnt receptors and suppressors further determine the Wnt-dependency. Given that Wnt morphogens are short-lived molecules with limited diffusion distance, hepatocytes that are directly adjacent to the Wnt reservoir show more robust effects^41^. Prototypic Wnt downstream targets, including *Glul* and *Aqp9* showed a marked reduction upon the loss of vascular Wnt factors, whereas CYPs were downregulated to a lesser extent. This may be due to the fact that CYPs are co-regulated by different signalling pathways, including HIFs or via pericrine Rspo3^42,72,73^. Yet, it could also mean that thresholds to activate Wnt-regulated CYPs are much lower than for other Wnt downstream molecules. Surprisingly, the data further revealed a similar spatially distinct Wnt-machinery in LSEC with high expression of Wnt receptor complexes in the pericentral zone and the periportal bias of Wnt suppressor expression, suggesting a zonated Wnt signalling activity in the hepatic vasculature. In line with the data, a similar Wnt zonation within LSEC was observed in a recent human liver atlas. The study further highlighted that the human pericentral zonation bias is independent of lobule size^74^. Future work may dissect the number of liver lobes, their corresponding hepatic vascular architecture, and haemodynamics, and correlate these to liver zonation.

LSEC have dual functions in the liver, serving both as vascular and lymphatic EC, which is also reflected in their molecular makeup^22,75^. In lymphatic EC, oscillating shear stress was identified to trigger an autocrine canonical Wnt signalling axis that is crucial for lymphatic valve development^76^. Moreover, flow was shown to control the expression of the mechanosensitive gap junctional channels Cx37 and Cx43 that mediate calcium influx^77,78^. In the liver, endothelial Cx37 and Cx43 were expressed in the central vein and precisely modulated by both flow and the Wnt signalling pathway, highlighting the hybrid phenotype of LSEC by mimicking lymphatic valve EC. Lymphatic gene deletion of Cx37, Cx43 or both results in lymphedema and premature lymphatic valve development with disorganised lymphatic EC morphology and integrity^79^, which correlates with macroscopic defects in Wnt-deficient LSEC. Functionally, Cx37 regulates the vascular tone by mediating calcium-dependent intercellular communication between EC and vascular smooth muscle cells^80^. In the brain vasculature, gap junctional molecules are crucial for the propagation of vasodilation, thereby regulating blood flow throughout the arteriovenous axis^81^. In line with this, Wnt-deficient LSEC showed reduced Cx37 and Cx43 expression and consequently impaired calcium mobilisation, which reduced the vascular tone in vitro. Collectively, the data reveal that biomechanical activation, mediated by blood perfusion, serves as the zonation-enforcing parameter in the liver. EC act as a cellular decoder of biophysical parameters, hence, orchestrating overall liver function. Molecularly, biomechanical milieu activates a specialised endothelial gene programme that sustains vascular Wnt, which induces expression of gap junctional molecules, thus orchestrating instructive EC cell communication and thereby enforcing spatially distinct liver function (Supplementary Fig. 14).

## Methods

Wildtype C57BL/6J mice were purchased from Janvier Labs. The transgenic lines B6 *Evi/Wls*^fl/fl^ × Cdh5-Cre^ERT2^ and B6 *Rspo3*^fl/fl^ × Cdh5-Cre^ERT2^ were described previously^82,83^. The double mutant line was generated by crossing *Evi/Wls*^fl/fl^ and *Rspo3*^fl/fl^ with B6 Cdh5-Cre^ERT2^ line. Cre-positive mice used in this study were homozygous for the floxed alleles and heterozygous for Cre. Cre-negative littermates that were homozygous for the floxed alleles served as controls. All animals were housed and maintained in pathogen-free animal facilities of the German Cancer Research Centre. In brief, all mice were housed on a temperature controlled 12 h light/12 h dark cycle and had free access to water and food *ad libitum*. In vivo experiments were performed under the guidelines of the institutional and governmental regulation approved by the regional council Karlsruhe (DKFZ305, DKFZ370, DKFZ434, G-255/18, G-107/18, G-251/20 and G-36/22).

For the samples of portosystemic shunt, liver sections were shared and collected from Heikenwalder lab^29^ and Kiefer lab. For the murine liver samples directly exposed to normobaric hypoxia (10% O_2_), the samples were obtained from Korff lab^40^. In brief, mice were exposed to normobaric hypoxia for 3 or 7 days in a hypoxia chamber (Biospherix) with free access to water and food *ad libitum* with 10% oxygen and 90% nitrogen.

### Patient specimen

Patient samples were obtained under licence 2022-140_1_S_KH approved by the local ethics committee of TUM/MRI under the legal guidelines of the Government of Upper Bavaria (BayKrG Art. 27 Abs. 4), with a waiver of informed consent due to retrospective and anonymised secondary use of clinical specimens. The samples were subjected to immunostaining for GS.

### Tamoxifen treatment

To induce genetic recombination, 6–10 weeks old male and female mice were intraperitoneally (i.p.) injected with 2 mg/kg tamoxifen dissolved in 50 μl of corn oil for 5 consecutive days followed by a two-week washout period. For long-term tracing experiments, mice were sacrificed 3, 6 and 12 months after the initial genetic recombination. To induce genetic recombination in vitro, hydroxytamoxifen (4-OHT; Sigma) was dissolved in DMSO (Applichem) at a stock concentration of 20 mM and primary LSEC were treated with a final concentration of 2 μM of 4-OHT for two days with daily medium changes. Both female and male mice were included in our in vitro study.

### Partial hepatectomy

Two-third PHx was performed as described previously^22^. In brief, mice were anaesthetised with a mixture of ketamine (Bela-Pharm, 100 mg/kg body weight) and xylazine (Bayer, 10 mg/kg body weight) by i.p. injection. Next, mice were put on a temperature-controlled pad and eyes were protected from drying by using an eye cream (Bayer). Mice were laid on their back and skin and peritoneum were incised to allow full access to the liver. Subsequently, the left lateral lobe and the median lobe of the liver were ligated using 4-0 silk sutures (Ethicon) and resected. For one-third PHx, the left lateral lobe of the liver was resected. Metamizole (Serumwerk Bernburg) was used as a post-surgical analgesic treatment (200 mg/kg body weight for subcutaneous injection and 800 mg/kg body weight for *ad libitum*) during the first 48 h post-surgery. Mice were euthanised at the indicated time points using cervical dislocation. Both male and female mice were incorporated in the study.

### Ex vivo perfusion

Mice were first sacrificed by cervical dislocation and livers were exposed for ex vivo perfusion. The portal vein was cannulated with 27 G winged infusion system (B.Braun) and connected to a peristaltic pump (Ismatec). Subsequently, livers were perfused using 37 °C preheated Krebs–Henseleit saline solution (HiMedia) supplemented with sodium bicarbonate (Sigma) for 30 min. To mimic the physiological flow, the flow rate of the pump was set to 4 ml/min. In parallel, 8 ml/min of flow rate was applied to mimic the accelerated flow^23,38^. Both male and female mice were included in the study.

### Pharmacological in vivo treatments

To induce vasodilation, female and male mice were treated with Riociguat (AdooQ Bioscience). The drug was dissolved in DMA (Sigma) in a concentration of 100 mg/ml and stored at −20 °C. Before the experiment, Riociguat/DMA solution was further diluted in 20% 2-Hydroxypropyl-β-cyclodextrin/H_2_O solution (Sigma) to reach a final concentration of 2.5 mg/ml^37^. Mice were treated with Riociguat (10 mg/kg body weight) or vehicle control twice a day via oral gavage for two days.

To induce hypoxia in vivo, female and male mice were treated with Roxadustat (Selleckchem), a HIF prolyl hydroxylase Inhibitor. Roxadustat was dissolved in DMSO in a concentration of 50 mg/ml and stored at −20 °C. The working solution was freshly prepared by further diluting the solution with a sterile PBS (Sigma) in a concentration of 1 mg/ml^84^. For the pulse treatment, wild-type mice were injected i.p. with a dose of 10 mg/kg and sacrificed 4 h after the injection. For the long-term treatment, wild-type mice received daily i.p. injections for five consecutive days at a dose 10 mg/kg body weight.

To reduce hepatic oxidative stress, NAC (Sigma) was added to the drinking water of wild-type animals at a concentration of 1 g/L for two weeks^85^. Both male and female mice were incorporated in the study.

### Isolation of hepatocytes and liver sinusoidal endothelial cells

The optimised protocol of LSEC isolation with a minimum shear activation was adapted from the previous studies^22^. In brief, both female and male mice were first sacrificed by cervical dislocation and livers were exposed for in situ perfusion. For this, the vena cava was fixed using a 27 G winged infusion set connected to the tubing of a peristaltic pump. The liver was perfused with 37 °C pre-warmed liver perfusion medium (Gibco) at 4 ml/min for 2 min, followed by 37 °C pre-warmed liver digestion medium (Gibco) supplemented with 20 μg/ml Liberase TM (Roche) at 3 ml/min for 5 min. Perfused livers were explanted and placed into a Petri dish with a pre-warmed RPMI medium (Gibco). After removing the liver capsule membrane, the tissue was dissociated by gentle shaking in a final volume of 40 ml of RPMI.

For hepatocyte collection, the single cell suspension was filtered through a 100μm cell strainer (BD Bioscience) and centrifuged once at 50 × *g* and 4 °C for 5 min. The resulting cell pellet contained hepatocytes, which were used for further experiments.

For the isolation of non-parenchymal hepatic cells (NPC), the supernatant was collected and centrifuged for an additional 5 min at 50 × *g* and 4 °C to remove excessive parenchymal cells. The supernatant was collected, and the final NPC population was obtained by centrifugation at 300 × *g* and 4 °C for 10 min. Red blood cells were lysed by incubating the isolated cells in ACK buffer for 5 min. To purify LSEC, total NPC were resuspended in 200 μl of FACS buffer containing 5% FCS and incubated with 20 μl of mouse CD146 MicroBeads (Miltenyi Biotec) for 20 min on ice. The cells were washed once, resuspended in 1 ml of FACS buffer and loaded onto a LS column (Miltenyi Biotec) and cells were purified according to the manufacturer’s instructions. The isolated LSEC were then processed for flow cytometry, frozen for further molecular analyses or taken into cell culture as freshly isolated primary LSEC.

### Flow cytometry and spatial cell sorting

For hepatocyte cell sorting, enriched isolated hepatocytes (see detailed protocol above) were resuspended in FACS buffer and sorted using BD FACS Aria I and II (BD Bioscience). Hepatocytes were gated based on (1) forward scatter area (FSC-A) and side scatter area (SSC-A) to exclude NPC and cell debris, (2) forward scatter width and forward scatter height (FSC-H), as well as side scatter width and side scatter height to exclude cell doublets, and (3) FxCycle Violet staining at a dilution of 1:1000 (Invitrogen) to exclude dead or damaged cells. For spatial sorting, hepatocytes were stained with CD73-FITC (BioLegend) and CD324-PE (BioLegend) at a dilution of 1:200 on ice for 20 min. Cells were washed twice and resuspended in 1 ml FACS buffer. To obtain hepatocytes from different zones, the previously described FACS gating strategy was applied (Supplementary Fig. 6a, m)^53^.

For FACS sorting of LSEC, the NPC cell population was stained with CD45-FITC (BD Bioscience), CD31-APC or PE (BD Bioscience), and CD146-PE-Cy7 (Biolegend) at a dilution of 1:200 for 20 min. Stained cells were washed and resuspended in 1 ml FACS buffer, and cell populations were sorted using a BD FACSAria cell sorter platform. LSEC were isolated based on (1) FSC-A and SSC-A to exclude cell debris and cell clumps, (2) FSC-A and FSC-H to exclude cell doublets, (3) FxCycle Violet stain at a dilution of 1:1000 to exclude dead or damaged cells, and (4) CD45 negative, and CD31 and CD146 double positive staining to exclude contaminating cell types. To perform spatial sorting of LSEC, a previously described strategy was adapted^22^. In brief, the NPC cell population was additionally stained with CD117 (c-Kit)-APC (BioLegend) at a dilution of 1:200, and LSEC from the distinct zones were sorted based on their c-Kit expression profile (Supplementary Fig. 6b, n).

For FACS-based cell size analysis, spatial sorting of LSEC was performed to distinguish portal, midzonal, and central LSEC. FSC-A was further recorded according to the previously described FACS gating strategy (Supplementary Fig. 9j). The geometric mean of FSC-A was calculated, and the abundance of LSEC according to its FSC-A value was plotted on FlowJo (BD Bioscience, v.10).

### Cell culture and in vitro studies

Freshly isolated primary LSEC (see above for detailed protocol) were seeded into 6-well plates at a density of 1.0 × 10^6^ cells per well. The cells were cultured in a complete Endothelial cell medium (Cell biologics) with full supplementation according to the manufacturer’s instructions and maintained at 37 °C under 5% CO_2_.

### Manipulation of hypoxia in vitro

Freshly isolated primary LSEC obtained from wild-type mice were cultured in a 1% oxygen cell culture chamber at 37 °C for 24 h and cells were collected for further analysis. To pharmacologically mimic hypoxic conditions, cells were treated with 100 and 200 μM of cobalt-(II)-hexahydrate (CoCl_2_; Sigma) for 24 h. Cells were then washed and processed for further analysis.

### Manipulation of shear stress in vitro

To induce endothelial shear response in vitro^86^, freshly isolated wild-type primary LSEC were placed on a temperature-controlled orbital shaker (Edmund Buehler) set to 37 °C and were shaken at 90 rpm mimicking low shear conditions with a shear of 7 dynes/cm^2^ or at 200 rpm simulating high shear conditions with a shear of 14 dynes/cm^2^. These cells were shaken for 12 h and samples were collected for secondary analysis.

To discriminate between laminar shear and oscillatory shear, shear stress was induced using the in vitro fertilization (IVF) dish (Falcon) under an orbital shaker. Shear force at the periphery area of IVF dish mimics laminar shear. Whereas the shear force at the centre area of the IVF dish mimics oscillatory shear. Eight wild-type mice were pooled to isolate 40 million primary LSEC. Next, two million LSEC were seeded either on the central area or the peripheral area of the IVF dish overnight. For the dose-effect experiment, a different magnitude of shear stress was applied using an orbital shaker (0, 7, 14 dynes/cm^2^) for 12 h. To capture the temporal kinetics of the LSEC transcriptome, two million LSEC (pooled from eight mice) were seeded on the central area of the IVF dish overnight and oscillating shear stress was applied using an orbital shaker at 14 dynes/cm^2^ for different time points (30 min, 1, 6 and 12 h).

To further evaluate the shear response of primary LSEC, a conventional IBIDI microfluidic pump system (IBIDI) was used. In brief, 1.0 × 10^6^ primary LSEC were seeded on 0.1% Gelatin-coated IBIDI Luer 0.4 mm slide (IBIDI) overnight. The slides were then subjected to either static control, laminar shear, or oscillatory shear. To induce laminar shear, a single IBIDI pump unit was acquired and set to provide a shear of approximately 14 dynes/cm² overnight. Conversely, two IBIDI pump units were used to induce oscillatory shear stress with a magnitude of approximately 14 dynes/cm² overnight. After the experiments, cell morphology was confirmed on a microscope and cells were directly lysed for mRNA extraction.

### Pharmacological manipulation of Wnt signalling pathway in vitro

To activate canonical Wnt signalling, freshly isolated primary LSEC were treated with 3 μM CHIR99021 (Selleckchem), a GSK3β inhibitor, for 24 h. Conversely, the canonical Wnt signalling pathway was inhibited using a porcupine inhibitor LGK974 (Selleckchem) at a concentration of 10 nM for 24 h. To induce the non-canonical Wnt signalling pathway, the cells were treated with recombinant WNT5A (R&D systems) at 10, 100 or 200 ng/ml for 24 h.

To mimic the central venous milieu, freshly isolated primary LSEC were treated for 24 h with 200 ng/ml of recombinant WNT2 (Cusabio) or 100 ng/ml of recombinant WNT9B (R&D).

### TCF reporter assay

To test for the secretion of bioactive Wnt, wild-type primary LSEC were cultured in serum free EC medium and subjected to 12 h of shear activation. The conditioned medium (CM) was collected. 1.0 × 10^5^ HEK293T cells transfected with the TCF4/Wnt-mCherry construct were seeded in a six-well dish and cultured with complete DMEM (i.e., supplemented with 10% FCS and 1% penicillin/streptomycin). After one day, the complete DMEM medium was replaced with a 1:1 mixture of complete DMEM medium and primary LSEC CM from the different shear conditions. This mixture was further supplemented with either 100 ng/ml of canonical WNT3A (PeProtech) or 1 μM of CHIR99021 (Selleckchem) to serve as positive controls. HEK293T reporter cells were incubated for 12 h and the TCF4/Wnt activity was recorded using flow cytometry. To quantitatively assess the Wnt-activation, the geometric mean intensity of TCF4-induced mCherry was calculated using FlowJo (BD Bioscience, v.10).

### Live cell calcium imaging

Primary LSEC were isolated from the double mutant and control mice. 1.0 × 10^6^ cells were plated in a 6-well plate and genetic recombination was induced as mentioned above. For flow-dependent study, primary LSEC were seeded on the periphery area of the IVF and laminar shear was applied using an orbital shaker. Genetically modified primary LSEC and control LSEC were incubated with 3 μM of Cal-520 (AAT Bioquest) in EC medium in a humidified incubator at 37 °C for 30 min. For a control experiment, cells were co-incubated with 100 μM of Carbenoxolone (CBX, Sigma-Aldrich) for 30 min. The cells were washed using Hanks Buffer (Gibco) and submerged in calcium-depleted Hanks Buffer (Gibco). Next, the plate was placed on Zeiss Widefield Cell Observer. Internal calcium storage was depleted using 1 μM of Thapsigargin (Sigma) and the image was acquired for the baseline control. Subsequently, 1 mM of CaCl_2_ was added to the cells submerged with the calcium-depleted Hanks Buffer and the image was acquired every second for 5 min. Next, 1 μM of ionomycin (Sigma-Aldrich) was added to the solution as a positive control.

### RNA extraction, cDNA synthesis, and real-time quantitative RT-PCR

For RNA extraction of whole liver lysates, liver pieces were homogenised using TissueLyzer (Retsch) in TRIzol (Sigma) and RNA was purified using chloroform (Carl Roth) and isopropanol (Sigma). RNA extraction from cell pellets (sorted or cultured cells) was performed using the GeneElute mammalian Total RNA Isolation Kit (Sigma) according to the manufacturer’s instructions. 500 to 1000 ng of total mRNA was reverse transcribed into cDNA using the QuantiTect Reverse transcription kit (Qiagen) following the manufacturer’s instructions. Gene expression analysis was performed by quantitative PCR using TaqMan reactions (Thermo Fisher Scientific) and StepOnePlus (Applied Biosystems). Gene expression levels were assessed using the Ct-method and normalised to the expression of the respective control gene, resulting in ΔCt-values. Relative gene expression was assessed by normalising ΔCt-values of individual samples to the average control ΔCt-value, resulting in ΔΔCt-values. Relative fold changes to control were then calculated as 2^−ΔΔCt^. The following taqman probes (Thermo Fisher Scientific) were used: *Klf2*, Mm01244979_g1; *Angpt2*, Mm00545822_m1; *Wnt2*, Mm00470018_m1; *Wnt9b*, Mm00457102_m1; *Gja1*, Mm00439105_m1; *Gja4*, Mm00433610_s1; *Klf4*, Mm00516104_m1; *Nos3*, Mm00435217_m1; Pecam1, Mm01242584_m1; Actb, Mm00607939_s1; Sox9, Mm00448840_m1; *Mfsd2a*, Mm01192208_m1; *Igfbp2*, Mm00492632_m1; *Hamp2*, Mm00842044_g1; *Axin2*, Mm00443610_m1; *Lgr5*, Mm00438890_m1; *Fzd4*, Mm00433382_m1; *Lrp6*, Mm00999795_m1; *Rspo3*, Mm01188251_m1; *Wls*, Mm00509695_m1; *Hif1a*, Mm00468869_m1; *Epas1*, Mm01236112_m1; *Slc2a1*, Mm00441480_m1.

### Protein extraction and immunoblotting

Proteins were isolated using RIPA buffer (Thermo Scientific) for cells and Trizol for liver tissues. Proteins were quantified using BCA (Thermo Scientific) and adjusted to the same concentration. Next, the samples were heated at 95 °C for 5 min, and subjected to 10% SDS-polyacrylamide gel electrophoresis. Proteins were transferred to PVDF membranes and were further blocked with 3% BSA (Gerbu) in TBST buffer. Following primary antibodies were used for immunoblotting: rabbit anti-mouse GJA1 (1:2000, Abcam), rabbit anti-mouse GJA4 (1:1000, Invitrogen), rat anti-mouse activated-CD29 (1:1000, BD), goat anti-mouse VEGFR3 (1:1000, R&D), rabbit anti-mouse Phospho-VEGFR3 Tyr1230 (1:1000, Affinity Biosciences), rabbit anti-mouse KLF2 (1:1000, Milipore), goat anti-mouse KLF4 (1:1000, R&D), mouse anti-mouse HIF1A (1:1000, Novus Biologicals), and rabbit anti-mouse Tubulin (1:1000, Cell Signaling). Consequently, membranes were washed with TBST, incubated with HRP-conjugated secondary antibodies: goat anti-rabbit HRP (1:5000, DAKO) and rabbit anti-goat HRP (1:5000, DAKO), developed with Western Blotting Lumino Reagent (Santa Cruz), and images acquired by Amersham Imager 600 (GE Healthcare). For reblotting, membranes were stripped using Re-blot strong solution (Millipore). Quantification of immunoblotting was performed using FIJI software (ImageJ, 1.54 g).

### Sample preparation of metabolomic screening

Blood plasma and liver pieces (lateral lobe) from 3-month-old mutant and control female and male mice were collected. The frozen liver pieces were snap frozen in liquid nitrogen and pulverised using a cooled ball mill (components were pre-cooled in liquid nitrogen). For the semi-targeted metabolite screening, 50 mg frozen ground liver tissue or 50 µL plasma were processed in 360 µL of 100% MeOH for 15 min at 70 °C with vigorous shaking. As an internal standard, 20 µL of Ribitol (Sigma) at a concentration of 0.2 mg/ml was added to each sample. The samples were then incubated in 200 µL of chloroform and shaken for 5 min at 37 °C. To separate polar and organic phases, 400 µL water was added and samples were centrifuged for 10 min at 11,000 × *g*. For the derivatisation, 700 µL of the polar (upper) phase was transferred to a fresh tube and dried in a speed-vac (vacuum concentrator) without heating.

### Derivatisation (methoximation and silylation)

Pellets of the aqueous phase after extraction were redissolved in 20 µL of methoximation reagent containing 20 mg/ml methoxyamine hydrochloride (Sigma) in pyridine (Sigma) and incubated for 2 h at 37 °C with shaking. For silylation, 32.2 µL of N-Methyl-N-(trimethylsilyl)trifluoroacetamide (Sigma) and 2.8 µL of Alkane Standard Mixture (50 mg/ml C_10_–C_40_; Fluka) were added to each sample. After 30 min of incubation at 37 °C, the samples were transferred to glass vials for GC/MS analysis.

### Gas Chromatography/Mass Spectrometry (GC/MS) analysis

GC/MS was performed using GC/MS-QP2010 Plus (Shimadzu) fitted with a Zebron ZB 5MS column (Phenomenex; 30 m × 0.25 mm × 0.25 µm). The GC was operated with an injection temperature of 230 °C and 1 µL sample was injected with split mode (1:5). The GC temperature program started with a 1 min hold at 40 °C followed by a 6 °C/min ramp to 210 °C, a 20 °C/min ramp to 330 °C and a bake-out for 5 min at 330 °C using Helium as the carrier gas with constant linear velocity. The MS was operated with ion source and interface temperatures of 250 °C, a solvent cut time of 7 min and a scan range (m/z) of 40–700 with an event time of 0.2 s. The GC-MS solution software (Shimadzu) was used for data processing.

For the measurement of cations, 30 µl extract (aqueous extract prepared for GC/MS analysis) was diluted with 270 µl ultra-pure water. Cations were separated isocratically with 30 mM methansulfonic acid for 27 min on an IonPac CS16 column (2 mm, Thermo Scientific) connected to an ICS-1000 system (Dionex) at a flow rate of 0.36 ml/min and quantified by conductivity detection after anion suppression (CERS-500 2 mm, suppressor current 43 mA) using ultra-pure standards (Sigma). Column temperature was set to 43 °C. Metabolomic data can be accessed on Supplementary Data 4.

### Sample preparation for proteomic analysis

Cell pellets were dissolved in an appropriate volume of freshly prepared 0.1% Rapigest (100 mM ammonium bicarbonate). The samples were sonicated at 10% amplitude on 50% Duty cycle with 20 cycles for 80 s. The cell lysates were centrifuged at 15,000 × *g* at 4 °C for 30 min and the protein concentration of the supernatants was measured using BCA protein assay kit (Pierce). A sufficient protein amount from each sample was reduced by incubating with 10 mM DTT at 56 °C for 30 min. The samples were then alkylated using chloroacetamide to a final concentration of 50 mM and incubated for 30 min at RT. The alkylation was quenched by adding DTT to a final concentration of 10 mM. Proteins were digested using trypsin–LysC at a 1:50 ratio and incubated overnight at 4 °C. The samples were acidified by adding an appropriate amount of 10% TFA and the acidity (pH <2) of the solution was checked. The samples were then incubated at 37 °C for 30 min and centrifuged for 30 min at maximum speed. The supernatants were transferred to fresh tubes and stored at −20 °C until the time of the analysis. Both female and male mice were incorporated in the proteomic screening.

### Liquid chromatography with tandem mass spectrometry (LC MS/MS) analysis

Peptides were separated using an EASY nanoLC 1200 System (Thermo Scientific), equipped with Acclaim PepMap 100, C18 trapping column (Thermo Scientific, 100 μm × 2 cm, 5 μm, 100 Å) and Acclaim PepMap RSCL 2 mM C18 analytical column (Thermo Scientific, 75 mm × 50 cm). The analytical column was heated to 45 °C in a MonoSLEEEVE column oven (Analytical Sales and Services). Sample loading on the trap column was performed under a constant pressure of 800 bar for a total volume of 22 μl of solvent A (0.1% Formic acid). Peptides were eluted from the trapping column with the following gradient at a flow rate of 300 nl/min: 0 to 4 min linear gradient from 3 to 8% solvent B (80% Acetonitrile and 0.1% Formic acid), 4 to 6 min linear gradient 8 to 10% B, 6 to 74 min linear gradient from 10 to 32% B, 74–86 min linear gradient from 32 to 50% B, 86 to 87 min gradient increase from 50 to 100% B, 87 to 94 min the gradient was kept to 100% B, 94 to 95 min linear gradient from 100 to 3% B, 95 to 105 min gradient was kept at 3% B.

The analytical column was coupled to a Silica PicoTip Emitter (Scientific Instrument Services) via a zero dead volume connection. Peptides were ionised using a Nanospray Flex nano spray source (Thermo Scientific) at a 2 kV voltage in the QE–HF mass spectrometer (Thermo Scientific). The ion transfer tube temperature was set to 275 °C. The following settings were used for data acquisition: Full scan MS1 acquisition was acquired within 350–1500 m/z scan range and 120,000 resolution. The maximum injection time (IT) was set to 32 ms and automatic gain control (AGC) to 3 × 10^6^ ions. Spectrum data type was set to profile. The top 20 most abundant precursor ions were selected for fragmentation. Ions with unassigned charges and charges of 1 or >5 were excluded. Dynamic exclusion was set to 40 s with a mass tolerance of ±10 ppm. For the MS2 scans, quadrupole was used with an isolation window of 2.0 m/z. For peptide fragmentation, higher-energy collisional dissociation was used at 26%. MS2 scans were acquired with an AGC target of 1 × 10^5^ or maximum IT of 50 ms and scan range window between 200 and 2000 m/z. MS2 spectra were acquired in centroid mode.

### Analysis of raw MS data

The raw data were searched using Maxquant (v 1.6.10.43) against the mouse canonical and reviewed database (*Mus musculus*, April, 2019). Enzyme digestion was set to trypsin allowing for a maximum of up to 2 missed-cleavages. Oxidation (M), Acetyl (Protein N-term), and deamidation (NQ) were set as variable modifications and carbamidomethyl (C) as fixed. Minimum unique peptides for protein identification were set to 1, while peptide and protein hits were filtered at a false discovery rate (FDR) of 1% with a minimal peptide length of 7 amino acids. The reversed sequences of the mouse canonical database were used as a decoy database for FDR calculation. Second peptide search option was enabled. Match between runs option was enabled with a matching time window of 0.4 min and the alignment time window set to 20 min. Dependent peptides option was deactivated. iBAQ calculation was also enabled. Label free quantification option was also enabled and LFQ min.ratio.count option was changed to 1. All other Maxquant options were left to their default settings. LFQ-intensity of hepatocytes and LSEC are available on Supplementary Data 4.

Further gene ontology enrichment and pathway analysis on MS data were performed as previously described using LFQ-Anaylst (v 1.2.4)^87^. In brief, the pathway analysis was computed based on the LFQ counts. Differentially expressed proteins were calculated using *limma* from *R* for the pair-wise comparison (adjusted *p* < 0.2 with Benjamini–Hochberg method & log2 fold change > 1.0), consequently, pathway analysis was performed.

### Immunofluorescence imaging

Liver lobes were fixed in 4% paraformaldehyde solution overnight and dehydrated in 30% sucrose prior to embedding in the OCT compound (Sakura Finetek). Cryosections (10 μm) were cut using a CM3050S cryostat (Leica) and attached to Superfrost Plus slides (VWR). Antigen retrieval was performed for APC, FZD4, and LRP6 staining by incubating the tissue section with Proteinase K (20 µg/ml, Gerbu Biotechnik) for 15 min at 37 °C. The sections were then submerged in a blocking buffer composed of PBS with 0.3% TritonX-100 (Sigma-Aldrich) and 10% FCS (Gibco) for 1 h in RT. Subsequently, the primary antibodies were diluted in the blocking solution and incubated overnight at 4 °C. Following primary antibodies were used for staining: rabbit anti-mouse GS (1:1000, Abcam); rabbit anti-mouse Cytochrome P450 2E1 (CYP2E1, 1:500, Abcam); goat anti-mouse VE-Cadherin (1:200, R&D); goat anti-mouse ICAM1 (1:200, R&D); mouse anti-mouse eNOS (1:200, Cell Signaling); goat anti-mouse Collagen IV (COLIV, 1:200, R&D Systems); rabbit anti-mouse GJA4 (1:200, Invitrogen); rabbit anti-mouse APC (1:100, Novus); goat anti-mouse FZD4 (1:100, Novus); goat anti-mouse LRP6 (1:100, Novus); rat anti-mouse KI67 (1:200, Invitrogen); rabbit anti-mouse HNF4A (1:200, Abcam); rabbit anti-mouse GJA4 (1:1000, Invitrogen). The sections were then washed three times using a wash buffer composed of PBS with 0.2% TritonX-100 and 5% FCS. Subsequently, sections were incubated with secondary antibody solution diluted in washing buffer at a dilution of 1:400 for 2 h at RT. The following secondary antibodies were used for staining: Alexa Flour 546 Phalloidin, Alexa Fluor 647 donkey anti-rabbit IgG (H + L); Alexa Fluor 568 donkey anti-goat IgG (H + L) (Thermo Fisher Scientific); Alexa Fluor 488 donkey anti-rat IgG (H + L). Stained sections were counterstained with 1:2000 Hoechst 33342 (Sigma-Aldrich) and mounted with Fluorescence Mounting Medium (Agilent Dako). Images were acquired with either Leica TCS SP5II (Leica) or Leica TCS SP8 (Leica) confocal microscope and image analysis was performed using Fiji software (ImageJ, 1.53t). For high resolution imaging, images were acquired using LSM 980 Airyscan (Zeiss) with Airyscan 2 modes focussing on resolution and sensitivity. The images were processed using ZEN blue (Zeiss).

For staining of hypoxic areas, pimonidazole staining (Hypoxyprobe) was applied according to the manufacturer’s protocol. In brief, mice were intravenously injected with pimonidazole (60 mg/kg) and organs were harvested after 1 h of exposure. Tissue sections were prepared as described above and immunostaining was carried out according to the manufacturer’s instructions.

Image analysis was performed using Fiji software (ImageJ, 1.53t). After region-of-interest (ROI) selection, GS, COLIV, LRP6, APC, FZD4, KI67, HNF4A, DAPI channels were masked using thresholding. For liver zonation, the percentage of GS^+^ area within the ROI was calculated. For vascular Wnt zonation, overlapping of FZD4^+^ and COLIV^+^, or LRP6^+^ and COLIV^+^, or APC^+^ and COLIV^+^ LSEC within the ROI were quantified. To quantify regenerating hepatocytes, KI67^+^/HNF4A^+^/DAPI^+^ triple-positive hepatocytes were considered proliferating hepatocytes. For analysing zone-specific hepatocyte proliferation, ROIs reflecting portal, midzonal, and central regions were defined based on the spatial coordination of GS^+^ hepatocytes and bile ducts. Subsequently, KI67^+^/HNF4A^+^/DAPI^+^ triple-positive hepatocytes in each of the three zones were quantified.

### Proximity ligation assay

The proximity ligation assays (PLA) were performed using the Duolink Proximity Ligation Kit (Sigma) and the manufacturer’s protocol was followed with minor modifications. Cryosections were prepared (7 μm) from the PFA-fixed liver samples of both female and male mice and blocked using the Duolink Blocking Solution at 37 °C for 60 min in a humid environment. Primary antibodies were diluted in Duolink Antibody Diluent in a 1:100 ratio and incubated at 37 °C overnight in a humid environment. The following antibodies were used for PLA: Rat anti-mouse ITGB1 (1:100, BD), Goat anti-mouse VEGFR3 (1:100, R&D), Rabbit anti-mouse Phosphotyrosine (1:100, Abnova), and Rabbit anti-mouse Phosphoserine (1:100, Abnova). The antibodies were further labelled with Duolink anti-rabbit MINUS probe (Sigma) and Duolink anti-goat PLUS probe (Sigma), while ITGB1 antibody was conjugated with Duolink in situ Probemaker PLUS (Sigma). PLA probe incubation and ligation were performed according to the manufacturer’s protocol. For signal amplification, Duolink In Situ Detection Reagents Orange (Sigma) was applied. For counterstaining, goat anti-mouse VE-Cadherin (1:200, R&D) or rat anti-mouse ITGB1 (1:200, BD) was used. Furthermore, rabbit anti-Glutamine synthetase (1:1000, Abcam) was pre-labelled with Alexa Fluor 647 using Zenon IgG labelling reagent (Thermo Scientific) and stained to distinguish liver zonation.

### In situ hybridisation assay

The in situ hybridisation assays were performed using the ViewRNA ISH Tissue Assay Core Kit (Thermo Fisher Scientific). The manufacturer’s protocol was employed with minor modifications. In brief, cryosections (7 μm) were fixed with PFA overnight at 4 °C followed by dehydration in ethanol, baked for 1 h at 60 °C, boiled for 20 min in pre-treatment solution, and further digested for 20 min in protease solution at 42 °C. Subsequently, the hybridisation was performed with *Gja1* (VB6-17027-VT) probe (Thermo Fisher Scientific) for 2 h at 42 °C. The signals were then pre-amplified and amplified according to the protocol at 42 °C. Next, *Gja1*-labelled sections were conjugated to the fast red substrate (Thermo Fisher Scientific) for visualisation. Consequently, goat anti-mouse VE-Cadherin (1:200, R&D) and rabbit anti-Glutamine synthetase (1:1000, Abcam) were treated for counterstaining.

### Shear stress–induced cell alignment

Phalloidin staining was performed on either static or shear activated primary LSEC isolated from mutant and control mice. First, 1.0 × 10^6^ of primary LSEC were seeded and were grown until they formed a monolayer. Next, in vitro gene deletion was induced using 4-OHT as described above. Cells were then placed in an orbital shaker for shear activation at 14 dynes/cm^2^ for 12 h. For peristaltic pump-induced shear stress, primary LSEC were seeded on 0.1% Gelatin-coated IBIDI Luer 0.4 mm slide (IBIDI) overnight. Subsequently, in vitro gene deletion was performed for 48 h. To avoid cell detachment, a transient pulse of low shear stress (3 dynes/cm²) was applied for 30 minutes, and ultimately, the LSEC were exposed to high laminar shear stress (11 dynes/cm²) for 24 h. Next, cells were fixed in 4% PFA for 30 min and blocked using a blocking solution composed of 0.3% TritonX-100 and 10% FCS mixed in PBS. Subsequently, phalloidin (Invitrogen) with cell nucleus staining was performed for 1 h in RT and cells were washed and submerged in PBS. Imaging was performed on Zeiss Widefield Axio CellObserver (Zeiss). Six random peripheral areas of each well were selected and imaged. The images were then quantified and analysed using Fiji (ImageJ, 1.53t). In brief, the stress fibres marked by F-actin were segmented using OrientationJ (ImageJ, 1.53t). Then the direction and angle of segmented filaments were calculated through Directionality (ImageJ, 1.53t). Alignment angle in respect to the direction of shear was calculated and visualised using Roseplot from R.

### Electron microscopy

Liver lobes were perfused and incubated in the fixative solution (2% formaldehyde, 2% glutaraldehyde, 1 mM CaCl2, 1 mM MgCl2, 100 mM Na-phosphate, pH of 7.2) for 2 h at 4 °C. Vibratome sections of 180 μm nominal thickness were post-fixed in 1% osmium tetroxide (buffered in 40 mM Na-Phosphate), dehydrated through graded steps of ethanol and embedded in epoxide (SERVA). Ultrathin sections of 50 nm nominal thickness were post-stained with uranyl and lead for imaging with a Zeiss EM 910 (Zeiss).

### Single cell RNA-sequencing

Single cell RNA-sequencing of FACS sorted hepatocyte and LSEC from Double-iECKO and control mice was performed using the CEL-seq2 protocol^52^. In brief, cells were sorted into 96-well plates containing 2.3 μl of lysis buffer composed of dNTPs (Invitrogen), 1% TritonX-100, ultra-pure water and barcoded primers, centrifuged at maximum speed and snap frozen on dry ice. For hepatocytes, 14 plates containing 1344 cells from four different Double-iECKO mice and other 14 plates consisting of 1344 cells from three different littermate control mice were prepared. For LSEC, 1344 cells from three different mutant mice and 1344 cells from three different control mice were prepared. Cells were lysed and released RNA was annealed to barcoded primers for 3 min at 65 °C. First strand synthesis was initiated using a reaction buffer consisting of 0.4 μl of First strand buffer (Invitrogen), 0.2 μl of DTT (Invitrogen), 0.1 μl of RNase inhibitor (Invitrogen), and 0.1 μl of Superscript IV (Invitrogen) and samples were incubated for 1 h at 50 °C followed by 10 min of heat inactivation at 80 °C. Subsequently, second-strand synthesis was performed according to the original CEL-Seq2 protocol. Barcoded cDNA from each well was pooled per plate and purified using AMPure XP Beads (Beckman Coulter). For linear amplification, the cDNA was subjected to in vitro transcription using MEGAscript T7 (Invitrogen) for 13 h at 37 °C. Excessive primers were removed using EXO-SAP (Thermo Fisher Scientific) and amplified RNA was fragmented using fragmentation reagents (Invitrogen). Amplified RNA fragments were purified using RNAClean XP Beads (Beckman Coulter) and sequencing libraries were prepared according to the original protocol. To account for the low RNA content of LSEC, LSEC libraries were amplified cDNA using 18 cycles for the final PCR, whereas hepatocyte libraries were amplified using 14 cycles, respectively. Both female and male mice were included in the experiment.

Sequencing library was purified from excess primers using AMPure XP Beads and quality controlled using 2100 Bioanalyzer (Agilent) and Qubit fluorometer (Invitrogen). Multiplexed libraries containing 14 pooled libraries were sequenced on a NextSeq550 (Illumina) using Paired-End 75 bp High Output kit to a depth of approximately 100,000 reads per cell. For sequencing, Read1 was set to a length of 13 bases, whereas Read2 was set to 50 bases. 5% PhiX (Illumina) was added to increase complexity and for error rate control.

### Single-cell RNA-seq analysis

Raw FASTQ files were demultiplexed by merging R1 reads containing the cell barcode and UMI with their corresponding R2 reads. *Subread* (v1.6.5)^88^ was employed to align the reads to the mm10 mouse reference genome. Next, the data was trimmed using *Samtools* (v1.0.9)^89^ and count matrices were generated by counting the transcripts for each gene based on the number of UMIs.

Quality control and data analysis were performed using the *Seurat* V3 package^90^. In brief, cells were filtered based on the number of detected genes (hepatocytes > 1000 genes and <7000 genes; LSEC > 500 genes and <4000 Genes) and log-normalised. Next, to account for batch effects, data integration was performed. For this, variable genes were detected in the dataset using the *FindVariableFeatures* function with default settings. A set of anchor genes for correction between batches was computed using the *FindIntegrationAnchors* function, and biological replicates were integrated using the *IntegrateData* function. The integrated dataset was scaled using *ScaleData* followed by principle component analysis using *RunPCA*. Dimension reduction was performed with the *RunUMAP* function using 10 PCA dimensions. Clustering of the data was performed using the *FindNeighbors* with 10 PCs and the *FindClusters* function with the resolution parameter set to 0.15. Differentially expressed genes (DEG) between the clusters were computed by Wilcoxon rank sum test using the *FindMarkers* function. Cell clusters that contain immune cell markers and stellate cell markers based on their DEGs were manually removed. Cluster markers were used for cluster annotation based on the marker genes that are enriched for the distinct hepatic zones (Supplementary Fig. 6f)^17^. *Vlnplot* and *Dotplot* functions were employed for the visualisation of the candidate genes.

For the density plots, gene scores were computed using the *AddModuleScore* function. Density plots of the gene scores of individual cells were generated using *ggplot2* (v 3.3.4) and Violin plots were plotted using the *Vlnpot* function in Seurat. The gene lists for computing gene scores were assembled from previously published literature^16,17,63,70,91^.

The central hepatocyte gene score was computed based on the expression of *Cyp2c29, Mup11, Cldn2, Ang, Gstm1, Inmt, Lect2, Gulo, Cyp2e1, Cyp1a2, Aldh1a1* and *Gstm4*.

The midzonal hepatocyte gene score was computed based on the expression of *Scd1, Mt1, Gm26870, Gm17535, Gm10801, Neu1, Igfbp2* and *Cyp8b1*.

The portal hepatocyte gene score was computed based on the expression of *Hal, Ftcd, Hsd17b6, Pck1, Hsd17b13, Aqp8, Arl4d, Elavl4, Etnppl, Alb, Cyp2f2, Arg1* and *Sfxn1*.

The liver detoxification gene score was computed based on the expression of *Cyp2c37, Gstm2, Cyp2c29, Cyp2e1, Gstm6, Gstm3, Cyp4a12b, Ccyp2d9, Cyp2c37, Gstm2, Fmo1, Gstt1, Gsta3, Cyp2d40, Gclm, Ugt3a2, Cyp8b1, Cyp2a12* and *Cyp2f2*.

The central LSEC gene score was computed based on the expression of *Wnt2, Wnt9b, Rspo3, Kit, Thbd, Slco2a1, Gas6, Igals1, Cd81* and *Ptgs1*.

The portal LSEC gene score was computed based on the expression of *Dll4, Angpt2, Efnb2, Itbp4, Ly6a, Msr1, Efnb1, Ntn4, Galnt15* and *Glul*.

The shear gene score was computed based on the expression of *Itpr1, Sytl2, Cebpd, Eln, Fgf18, Mturn, Fnip2, Fbln2, Neo1, Dusp1, Itga1, Ramp2, Ntn1, Aqp1, Lyst, Srl, Slc22a4, Mmp24, Rapgef4, Plcb4, Kcnn3, Jph4, Hmga2, Fen1, Fjx1, Uhrf1, Hmga1, Map4k4, Dkk1, Slc1a1, Sae1, Apln, Arhgap18, Arhgap24, Junb, Arhgef9, Cxcl3, Hmcn2, Arhgap21, Arhgap23, Arhgef7, Klf2, Klf4, Jun* and *Fos*.

The gap junctional molecule gene score was computed based on the expression of *Gja1, Gja4, Gja5, Gjb1* and *Gjb2*.

DEGs between genotypes were identified using DESeq2^92^ with a two-tailed Wald test and Benjamini–Hochberg. For this, pseudo-bulks for both hepatocytes and LSEC were formed, reflecting the biological replicates and DEG analysis was performed. DEGs were considered significant for adjusted *p* < 0.05 and log2 fold change > 1.0. DEGs were also calculated in between the spatial bin of the datasets and the respective spatial bins between the genotypes. For this, cells were classified based on the zonation score (see detailed description above) and pseudobulks reflecting the central, midzonal and portal hepatic zones were formed.

Gene set enrichment analyses (GSEA)^93,94^ was performed on DEGs ranked by fold change using the *clusterProfiler* v4.0.1 package in *R*^95^. Gene sets were considered enriched for normalised enrichment scores > 1.5, *p*.val < 0.05 and *q*.val < 0.1 for hepatocyte and *q*.val < 0.40 for LSEC.

For validation, a publicly available dataset was utilised that was deposited on the gene expression omnibus under the accession code GSE192742^55^. Cell count matrix of CD45^−^ murine was processed accordingly (Supplementary Fig. 7k).

### Assignment of zonation position

To each sequenced cell c, we assigned a “zonation position”$\,{\eta }_{c}$, which is a quantity indicating the spatial position of the cell along the central-to-portal zonation axis, as inferred from the cell’s expression of zonation markers. The expression of the portal and central markers was summed for each cell, yielding sums ${S}_{c}^{C}$ and ${S}_{c}^{p}$, and transformed into a zonation position η, such that ${\eta }_{c}=0$ being the portal extreme, and ${\eta }_{c}=1$ the central end. Values in between indicate mixed expression and hence an intermediate position. Details on how the expression sums were calculated are given below.

For the hepatocytes, we used a modified marker list which is composed of previously established landmark gene sets^17^ with six additional genes marked by an asterisk:

Markers for central hepatocytes: ${L}_{C}$ = (Alad, Aldh1a1, C6, Nat8f2, Cpox, Csad, Cyb5a, Cyp1a2, Cyp2c37, Cyp2c50, Cyp2e1*, Cyp3a11, Glu*, Gstm1, Hpd, Lect2, Mgst1, Oat, Pon1, Prodh, Rgn, Slc16a10).

Markers for portal hepatocytes: ${L}_{P}$ = (Alb*, Apof, Arg1, Apom, Asgr2, Asl*, Ass1*, Atp5a1, Cps1, C1s1, C8b, Cpt2, Cyp2f2*, Tkfc, Eef1b2, Elovl2, Fads1, Fbp1, Gc, Gnmt, Hsd17b13, Ifitm3, Igf1, Igfals, Ndufb10, Pck1, Pigr, S100a1, Serpina1c, Serpina1e, Serpind1, Serpinf1, Trf, Uqcrh, Vtn}.

We set ${k}_{{gc}}$ for the UMI count of gene g in cell c, ${s}_{c}={\sum }_{g}{k}_{{gc}}$ for the total UMI count of cell c, and ${x}_{{gc}}$ =  ${{k}}_{{gc}}/{s}_{c}$ for the fraction of UMIs of cell c for gene g. For all marker genes, these fractions were normalised by dividing them by the maximum expression of the gene, ${y}_{{gc}}={x}_{{gc}}/{x}_{g}^{\max }$. The maximum ${x}_{g}^{\max }$ is here taken over all hepatocytes of the same genotype.

For each cell c the sum of the maximum-normalised expression of the portal markers, ${S}_{c}^{P}={\sum }_{g\epsilon {L}_{P}}{y}_{{gc}}$, and of the central markers, ${S}_{c}^{C}={\sum }_{g\epsilon {L}_{C}}{y}_{{gc}}$ was calculated and a fraction, ${\eta }_{c}={S}_{c}^{C}/({S}_{c}^{P}+{S}_{c}^{C})$ was computed. In contrast to Seurat’s *AddModuleScore* function, we used a non-lognormalised score.

For LSEC, the same method was utilised using the following gene marker lists:

Markers for portal vein LSEC: Ang, Cd81, Emcn, Gas6, Gja1, Gja4, Id4, Ier3, Lfng, Plvap, Ptgs1, Rspo3, Tcim, Wnt2, Wnt9b.

Markers for central vein LSEC: Angpt2, Cxcl9, Dll4, Efnb1, Efnb2, Esm1, Id2, Insr, Jak1, Lama4, Ltbp4, Ly6a, Msr1, Ntn4, Osmr.

### Ranked position score

Given *Evi* and *Rspo3* double knockout influences the hepatic zonation pattern, specifically, it reduced the contrast of expression differences between periportal and pericentral cells, some of the zonal marker expressions were lost in Double-iECKO mice. Yet, other marker genes were still informative, thereby allowing us to assign the zonation position. However, the lost markers diluted to signal by reducing the distribution of ${\eta }_{c}$ in the Double-iECKO sample.

Furthermore, we assumed that η is monotonously related to the actual position of a cell along the central-to-portal axis. However, there was no reason to assume this relation to be linear, or that it is unaffected by treatment or batch effects. Hence, it seemed prudent to use the η values only to order the cells but not to make use of their absolute values.

Therefore, each cell’s η value was replaced with the cell rank ${\eta }_{Q}$, scaled to the unit interval, using the *cume_dist* function in R. Given a list of n cells with their η values, we ordered the cells by increasing η, and then assigned to each cell a rank score, which is simply the rank (zero-based) in the list, divided by n−1, such that the first (most portal-like) cell is scored as 0 and the last (most central-like) cell is scored as 1, while the score increases by 1/n for each cell in between. We referred this score as etaq.

Importantly, the computation was done separately for each experimental batch. For instance, each cell’s etaq values were ranked within a list comprising cells of the same genotype, from the same mouse, and those processed on the same plate. In this manner, we could avoid technical batch effects influencing the result and even out the signal dilution due to the gene knockout described above.

### Smoothing gene expression along zonation

#### Spline regression

To present the expression of a gene along the zonation axis, we used spline regression utilising generalised linear models of the negative-binomial family with logarithmic link with a 4-dimensional B-spline basis. For spline regression, we used a basis of 4 cubic B splines, as produced in *R* by the *bs* (basis spline) function of the *R* core package *splines*. Yet, the actual regression was performed by *glm.nb*, the GLM fit function for negative-binomial GLMs from the MASS package^96^.

The regression yielded coefficients ${\beta }_{{rm}}$, one for each of the 4 spline bases (r=1,..,4) and each mouse m. A smoothing spline for the gene of interest was obtained for each mouse m as ${\mu }_{m}({\eta }_{Q})=\,\exp ({\sum }_{r}{\beta }_{{rm}}{B}_{r}({\eta }_{Q}))$. Here, ${\eta }_{Q}$ reflected the *x*-axis for the plot, running from 0 to 1 and indicating the rank-normalised zonation position, and the ${B}_{r}(x)$ were the four basis functions of the standard 4th degree B spline basis. The fitted value $\mu ({\eta }_{Q})$ was the expected expression fraction of the gene in a cell at the position rank score ${\eta }_{Q}$.

For visualisation, the *y*-axis depicting $\mu ({\eta }_{Q})$ was shown with a square-root scaling to improve homo-scedasticity as the square root is the variance-stabilising transformation for the Poisson distribution.

A spatial map of vascular Wnt can be accessed at https://papagei.bioquant.uni-heidelberg.de/kiapp/.

### Quantification and statistical analysis

Statistical analysis and graphics were performed with GraphPad Prism v8 and Rstudio v1.2.5042. Unpaired two-tailed Student’s *t*-tests were performed when comparing two groups. In cases where more than two groups were being compared, one-way ANOVA followed by Bonferroni test was used. Statistical details are described in each figure legend. For pseudobulk DEG and its downsteam GSEA analysis, the samples were processed with a two-tailed Wald test with Benjamini–Hochberg correction. For the proteomics dataset, the samples were tested using a two-tailed *t*-test, followed by a Benjamini–Hochberg correction. Data are either presented as mean ± standard deviation (s.d.) or mean ± standard error (s.e.m.).

### Reporting summary

Further information on research design is available in the Nature Portfolio Reporting Summary linked to this article.

## Supplementary information

Supplementary Information

Description of Additional Supplementary Files

Supplementary Data 1

Supplementary Data 2

Supplementary Data 3

Supplementary Data 4

Reporting Summary

Transparent Peer Review file

## Source data

Source Data

## Data Availability

All raw sequencing data and processed count matrices for scRNA-seq have been deposited in Gene Expression Omnibus under accession code GSE230663. The raw mass spectrometry data were deposited in MassIVE under accession MSV000102526/PXD081294. The previously published dataset GSE192742 was used for analyses. Source data are provided with this paper.

## Code availability

The customised code for processing the CEL-seq2 can be found under https://github.com/simon-anders/. All custom code generated in this study is available at https://github.com/anders-biostat/hepatocytes-zonation/.
