# Supplementary material for: Haemodynamic control of zonated liver function via instructive vascular Wnt signalling
Source: Nat Commun. 2026 Aug 22;17:8821. doi: 10.1038/s41467-026-76966-7 (PMC13499805; doi:10.1038/s41467-026-76966-7)
Supplement: Supplementary file 1 [file 41467_2026_76966_MOESM1_ESM.pdf]

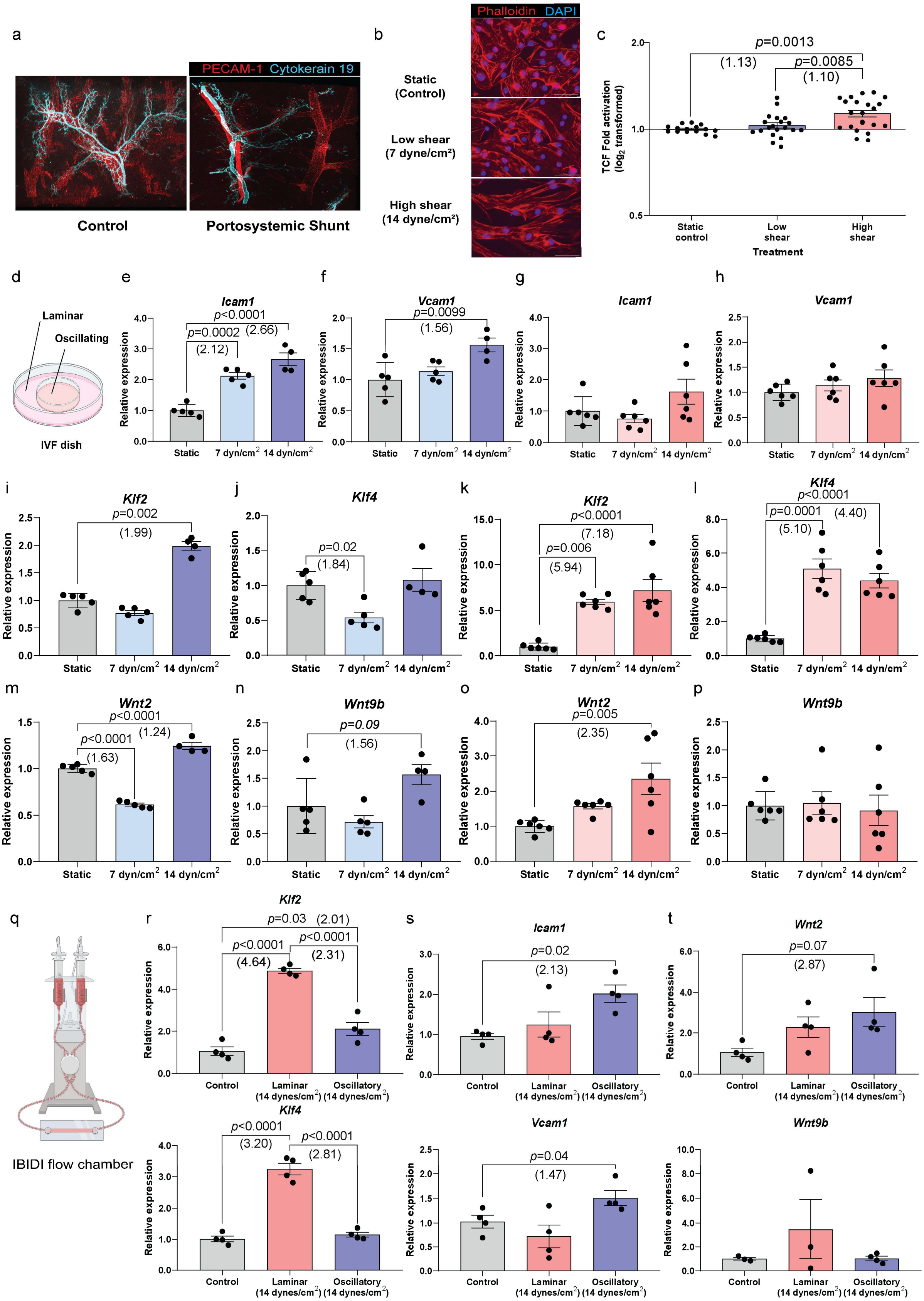
a
PECAM-1 Cytokerain 19
Control
Portosystemic Shunt
b
Phalloidin DAPI
Static (Control)
Low shear (7 dyne/cm²)
High shear (14 dyne/cm²)
c
p=0.0013
(1.13)
p=0.0085
(1.10)
TCF Fold activation (log2 transformed)
Static control
Low shear
High shear
Treatment
d
Laminar
Oscillating
IVF dish
e
Icam1
p<0.0001
p=0.0002 (2.66)
(2.12)
f
Vcam1
p=0.0099
(1.56)
g
Icam1
h
Vcam1
i
Klf2
p=0.002
(1.99)
j
Klf4
p=0.02
(1.84)
k
Klf2
p<0.0001
(7.18)
p=0.006
(5.94)
l
Klf4
p<0.0001
p=0.0001 (4.40)
(5.10)
m
Wnt2
p<0.0001
p<0.0001 (1.24)
(1.63)
n
Wnt9b
p=0.09
(1.56)
o
Wnt2
p=0.005
(2.35)
p
Wnt9b
Relative expression
Static
7 dyn/cm2
14 dyn/cm2
q
IBIDI flow chamber
r
Klf2
p=0.03 (2.01)
p<0.0001
p<0.0001
(4.64) (2.31)
Klf4
p<0.0001
(3.20) p<0.0001
(2.81)
s
Icam1
p=0.02
(2.13)
Vcam1
p=0.04
(1.47)
t
Wnt2
p=0.07
(2.87)
Wnt9b
Control
Laminar (14 dynes/cm2)
Oscillatory (14 dynes/cm2)

Supplementary Fig. 1: Fluid shear stress triggers vascular Wnt expression.

a, 3D-reconstructed wholemount staining of endothelium (PECAM1, red) in control and shunt animals. Portal vessels were identified by their close proximity to bile ducts (Cytokeratin 19, cyan). Diameter of portal tract / Artery at vessel root: control (left) 150 µm / 10 µm, Shunt (right) 200 µm / 70 µm. **b**, Each primary LSEC were cultured under either static (no shear), low shear (7 dynes/cm^2), or high shear (14 dynes/cm^2) conditions. LSEC morphology was assessed via phalloidin staining. **c**, Primary LSEC were cultured under either static (no shear), low shear (7 dynes/cm^2), or high shear (14 dynes/cm^2) conditions and the conditioned medium (CM) was collected. Fluorescence TCF reporter assay showing canonical Wnt activity in reporter cell line after treatment with CM (static, n=15; low shear, n=20; high shear, n=22). The geometric mean for fluorescent intensity was measured, and fold activation was normalised to the static control. **d**, Schematic overview of orbital shaker stimulated shear stress model. An in vitro fertilisation (IVF) dish was used to discriminate laminar shear (peripheral area of the culture dish) and oscillatory shear (central area of the culture dish)-regulated gene signatures. Created in BioRender. Lee, KH. (2026) https://BioRender.com/sy5gl4b. **e**, Relative gene expression of *Icam1*, **f**, *Vcam1* in each fresh isolated primary LSEC under different oscillatory shear conditions (static; n=5, 7 dynes/cm^2; n=5, 14 dynes/cm^2; n=4). **g**, Relative gene expression of *Icam1*, **h**, *Vcam1* in each fresh isolated primary LSEC under different laminar shear conditions (static; n=6, 7 dynes/cm^2; n=6, 14 dynes/cm^2; n=6). **i**, Relative gene expression of *Klf2*, **j**, *Klf4* in each fresh isolated primary LSEC under different oscillatory shear conditions. **k**, Relative gene expression of *Klf2*, **l**, *Klf4* in each fresh isolated primary LSEC under different laminar shear conditions. **m**, Relative gene expression of *Wnt2*, **n**, *Wnt9b* in each fresh isolated primary LSEC under different oscillatory shear conditions. **o**, Relative gene expression of *Wnt2*, **p**, *Wnt9b* in each fresh isolated primary LSEC under different laminar shear conditions. **q**, IBIDI flow chamber was employed to further corroborate the findings. High laminar flow (14 dynes/cm^2, n=4) and high oscillatory flow (14 dynes/cm^2, n=4) were applied. Created in BioRender. Lee, KH. (2026) https://BioRender.com/249ezru. **r**, Relative expression of laminar flow-sensitive *Klf2* and *Klf4.* **s**, Relative expression of oscillatory shear-regulated *Icam1* and *Vcam1*. **t**, Relative expression of vascular Wnt ligands, *Wnt2* and *Wnt9b*. () indicates fold difference. **c**,**e-p**,**r-t**, both female and male mice are incorporated in the study. **e**,**f**,**i**,**j**,**m**,**n**,**r**-**t**, Data are presented as mean±s.d. **c**,**g**,**h**,**k**,**l**,**o**,**p**, Data are presented as mean±s.e.m. **e**-**p**, **r**-**t**, Gene expression was normalised to *Pecam1* and fold change was assessed relative to their controls. **c**,**e-p**,**r-t**, One-way ANOVA followed by Bonferroni test was performed.

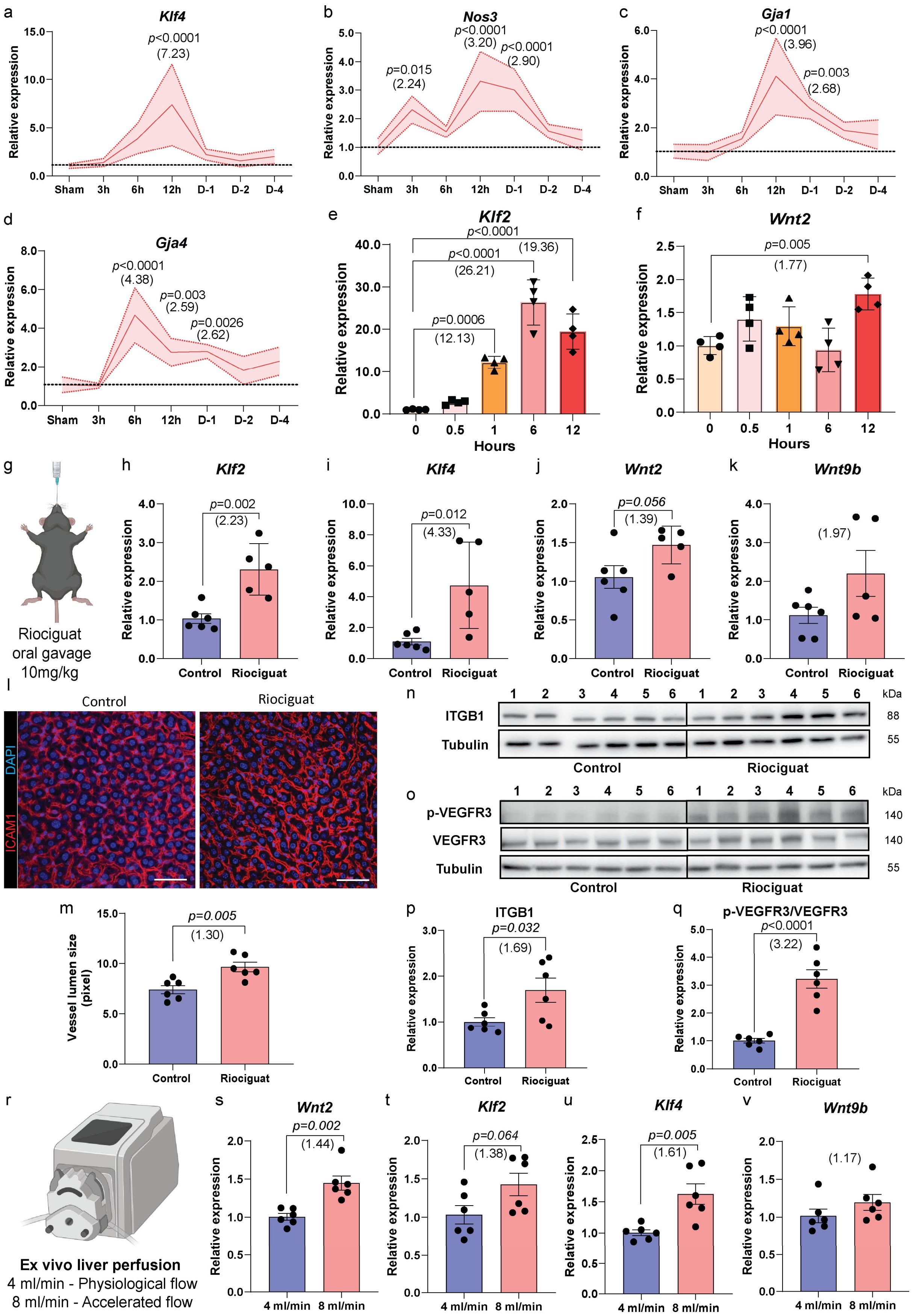

a
Klf4
p<0.0001
(7.23)
Relative expression
Sham 3h 6h 12h D-1 D-2 D-4
b
Nos3
p<0.0001
(3.20)
p<0.0001
(2.90)
p=0.015
(2.24)
c
Gja1
p<0.0001
(3.96)
p=0.003
(2.68)
d
Gja4
p<0.0001
(4.38)
p=0.003
(2.59)
p=0.0026
(2.62)
e
Klf2
p<0.0001
(19.36)
p<0.0001
(26.21)
p=0.0006
(12.13)
Hours
f
Wnt2
p=0.005
(1.77)
Hours
g
Riociguat
oral gavage
10mg/kg
h
Klf2
p=0.002
(2.23)
Control Riociguat
i
Klf4
p=0.012
(4.33)
j
Wnt2
p=0.056
(1.39)
k
Wnt9b
(1.97)
l
Control
Riociguat
DAPI
ICAM1
n
ITGB1
Tubulin
Control
Riociguat
kDa
88
55
o
p-VEGFR3
VEGFR3
Tubulin
140
140
55
m
Vessel lumen size (pixel)
p=0.005
(1.30)
p
ITGB1
p=0.032
(1.69)
q
p-VEGFR3/VEGFR3
p<0.0001
(3.22)
r
Ex vivo liver perfusion
4 ml/min - Physiological flow
8 ml/min - Accelerated flow
s
Wnt2
p=0.002
(1.44)
4 ml/min 8 ml/min
t
Klf2
p=0.064
(1.38)
u
Klf4
p=0.005
(1.61)
v
Wnt9b
(1.17)

Supplementary Fig. 2: Haemodynamic factors induce vascular Wnt expression.

a-d, Temporal kinetics of *Klf4*, *Nos3, Gja1* and *Gja4* expression in MACS-bead sorted LSEC during liver regeneration (sham: n= 5, 3h: n=6, 6h: n=4, 12h: n=6, D-1: n=6, D-2: n=6, D-4: n=6 mice). **e**,**f**, Temporal kinetics of *Klf2* and *Wnt2* expression in each primary LSEC under oscillating shear stress at different time points (0.5, 1, 6, and 12 h). **g**, Schematic overview of blood flow manipulation *in vivo* using the vasodilator Riociguat. Wildtype mice were treated for 48 h with 10 mg/kg Riociguat twice a day by oral gavage. Created in BioRender. Lee, KH. (2026) https://BioRender.com/6vkx373. **h**-**k**, Relative gene expression analysis of *Klf2*, *Klf4*, *Wnt2* and *Wnt9b* from MACS-bead sorted LSEC after Riociguat (n=6 mice) or vehicle control (n=6 mice) treatment. **l**, Representative ICAM1 immunostaining in the liver of vehicle control treated (right) and vasodilating agent Riociguat treated (left). Scale bar shows 100µm. **m**, Quantification of average lumen size. **n**, Immunoblotting of ITGB1 and corresponding Tubulin. **o**, Immunoblotting of VEGFR3, p-VEGFR3 and corresponding Tubulin from MACS-purified EC of mouse treated with either vehicle control (n=6 mice) or vasodilating agent Riociguat (n=6 mice). **p**,**q**, Relative protein expression of ITGB1, and p-VEGFR3 over VEGFR3. **r**, Schematic overview of blood flow manipulation *ex vivo* using the peristaltic pump. Two different flow rates were applied: 4 ml/min to mimic the physiological flow and 8 ml/min to mimic the accelerated flow. Created in BioRender. Lee, KH. (2026) https://BioRender.com/e5hmrw6. **s-v**, Relative gene expression of *Wnt2*, *Klf2*, *Wnt4* and *Wnt9b* in each MACS-purified LSEC either under physiological flow (n=4) or accelerated flow (n=4). () indicates fold difference. **a**-**f**, Relative RT-qPCR gene expression analysis was performed on MACS-bead sorted LSEC post-PHx. Data are presented as mean±s.d. Background Gene expression was normalised to *Pecam1,* and fold change was assessed relative to control (dotted line). **a-d**, Male mice were included in the study. **e**,**f**,**h-k**,**m-q**,**s-v**, Both female and male mice were incorporated in the study. **h-k**,**m**,**p**,**q**,**s-v**, Data are presented as mean±s.e.m. Gene expression was normalised to *Pecam1,* and fold change was assessed relative to controls. **h-k**,**m**,**p**,**q**,**s-v**, Two-tailed t-test was performed. **a**-**f**, One-way ANOVA followed by the Bonferroni test was performed.

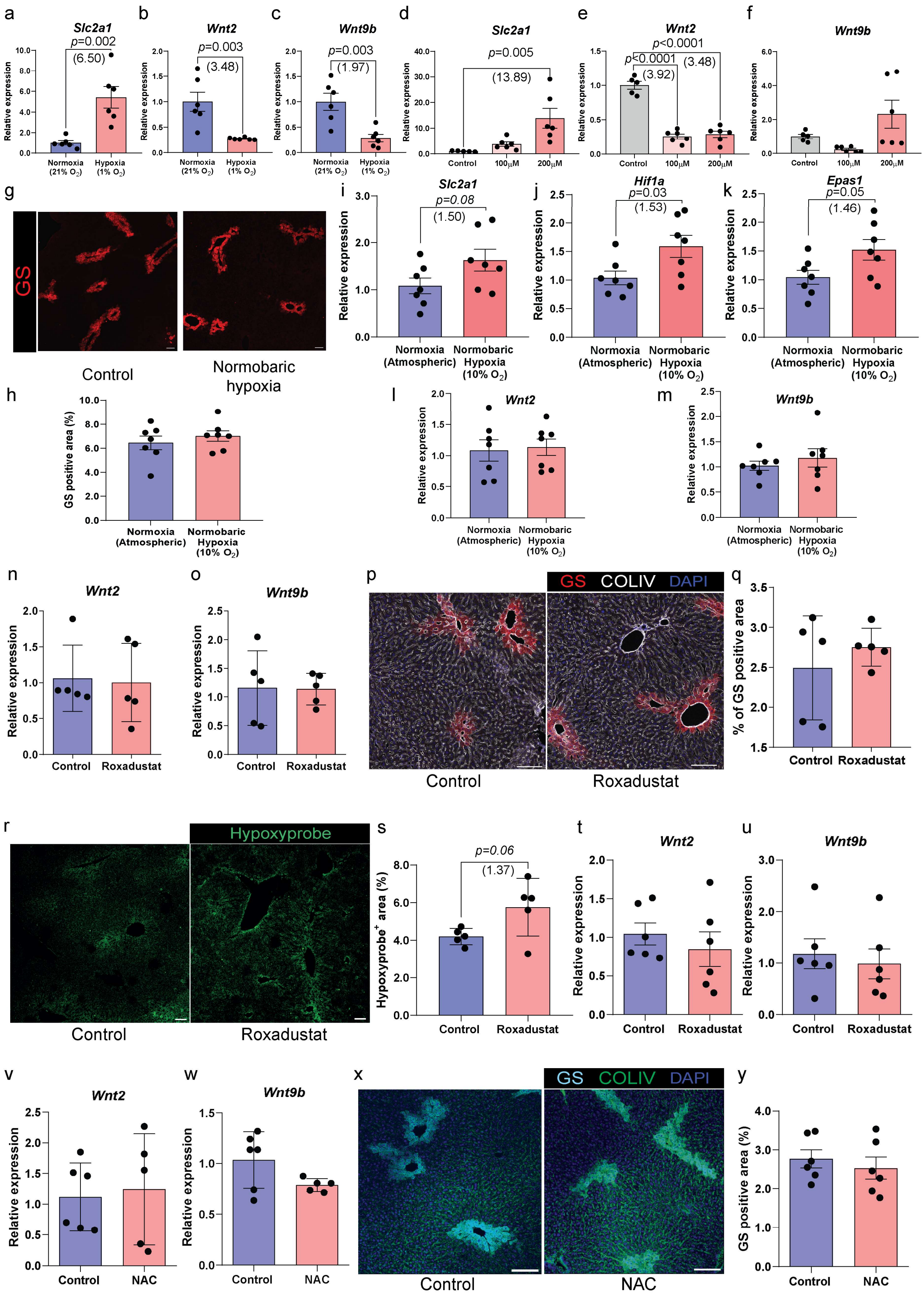
a
Slc2a1
p=0.002
(6.50)
Relative expression
Normoxia (21% O2)
Hypoxia (1% O2)
b
Wnt2
p=0.003
(3.48)
c
Wnt9b
p=0.003
(1.97)
d
Slc2a1
p=0.005
(13.89)
Control
100μM
200μM
e
Wnt2
p<0.0001
p<0.0001
(3.92)
(3.48)
f
Wnt9b
g
GS
Control
Normobaric hypoxia
h
GS positive area (%)
Normoxia (Atmospheric)
Normobaric Hypoxia (10% O2)
i
Slc2a1
p=0.08
(1.50)
j
Hif1a
p=0.03
(1.53)
k
Epas1
p=0.05
(1.46)
l
Wnt2
m
Wnt9b
n
Wnt2
Control
Roxadustat
o
Wnt9b
p
GS COLIV DAPI
q
% of GS positive area
r
Hypoxyprobe
s
p=0.06
(1.37)
Hypoxyprobe+ area (%)
t
Wnt2
u
Wnt9b
v
Wnt2
NAC
w
Wnt9b
x
GS COLIV DAPI
y
GS positive area (%)

Supplementary Fig. 3: Hypoxia and oxidative stress are dispensable for vascular Wnt induction.

a,**b**,**c**, Relative *Slc2a1*, *Wnt2* and *Wnt9b* gene expression of each primary LSEC from wildtype mice either under atmospheric oxygen (21%, n=6) or hypoxic (1%, n=6) conditions. **e**,**f**, Relative *Slc2a1*, *Wnt2* and *Wnt9b* expression in each primary LSEC under different cobalt chloride ($CoCl_2$) concentrations overnight (n=6). **g**, Cdh5-TomCreERT2 mice were kept either under atmospheric oxygen (21%) or normobaric hypoxic (10%) conditions. Murine liver samples were obtained 7 days after the induction of hypoxic challenge. Representative immunofluorescence image of GS (red) in the liver of mice exposed to 7 days of normobaric hypoxia (n=7 mice) or atmospheric oxygen (n=6 mice). Scale bars, 100µm. **h**, Corresponding quantification of GS positive area (bottom panel). **i**,**j**,**k**, Relative expression of hypoxia-regulated genes, *Slc2a1*, *Hif1a*, and *Epas1*. **l**,**m**, Relative *Wnt2* and *Wnt9b* gene expression in whole liver lysates of mice exposed to 7 days (bottom panel) of normobaric hypoxia or atmospheric oxygen, respectively. **n**,**o**, Relative *Wnt2* and *Wnt9b* expression in LSEC isolated from mice pulse-treated with HIF-stabilizer Roxadustat (n=5 mice) or vehicle control (n=5 mice) *in vivo*. **p**, Representative immunofluorescence image of GS (red) in the liver of mice treated with Roxadustat (n=5 mice) or vehicle control (n=5 mice). Scale bars, 100µm. **q**, Percentage of GS positive area in the whole liver section for Roxadustat or vehicle control treatment. **r**, Hypoxyprobe labelling of liver after Roxadustat administration. Liver sections from either vehicle control (n=5 mice) or HIF prolyl-hydroxylase, Roxadustat, treated (n=5 mice) mouse were subjected to hypoxyprobe labelling. Hypoxic area is marked by hypoxyprobe (green). Scale bar shows 100µm. **s**, Quantification of hypoxyprobe labelled area comparing Roxadustat treated and its vehicle control. **t**, Relative *Wnt2* and **u**, *Wnt9b* expression in LSEC isolated from mice treated long-term with Roxadustat or vehicle control (Roxadustat, n=6 mice; vehicle control, n=6 mice). **v**, Relative *Wnt2* and **w**, *Wnt9b* expression in LSEC isolated from mice treated with antioxidant agent N-acetyl Cysteine (NAC, n=6 mice) or vehicle control (n=6 mice) *in vivo*. **x**, Representative immunofluorescence image of GS (cyan) on liver sections of mice treated with NAC or vehicle control. Scale bars, 100µm. **y**, Corresponding quantification of GS positive area in the whole liver section. () indicates fold difference. **a-f**,**i-o**,**q**,**s-w**,**y**, Both female and male mice were included in the study. **n**,**o**,**q**,**s**,**v**,**w**, Data are presented as mean±s.d. **a**-**f**,**h**-**m**,**t**,**u**,**y**, Data are presented as mean±s.e.m. **a**-**f**,**i**-**o**,**t**-**w**, Gene expression was normalised to *Pecam1* and fold change was determined relative to controls. **i**-**m**, mRNA was collected from the whole liver lysate. **a**-**f**,**n**,**o**,**t**-**w**, mRNA was collected from MACS-bead sorted LSEC. **a**-**c**,**h**-**o**,**q**,**s**-**w**,**y**, Two-tailed unpaired *t*-test was performed. **d**-**f**, One-way ANOVA followed by Bonferroni test was performed.

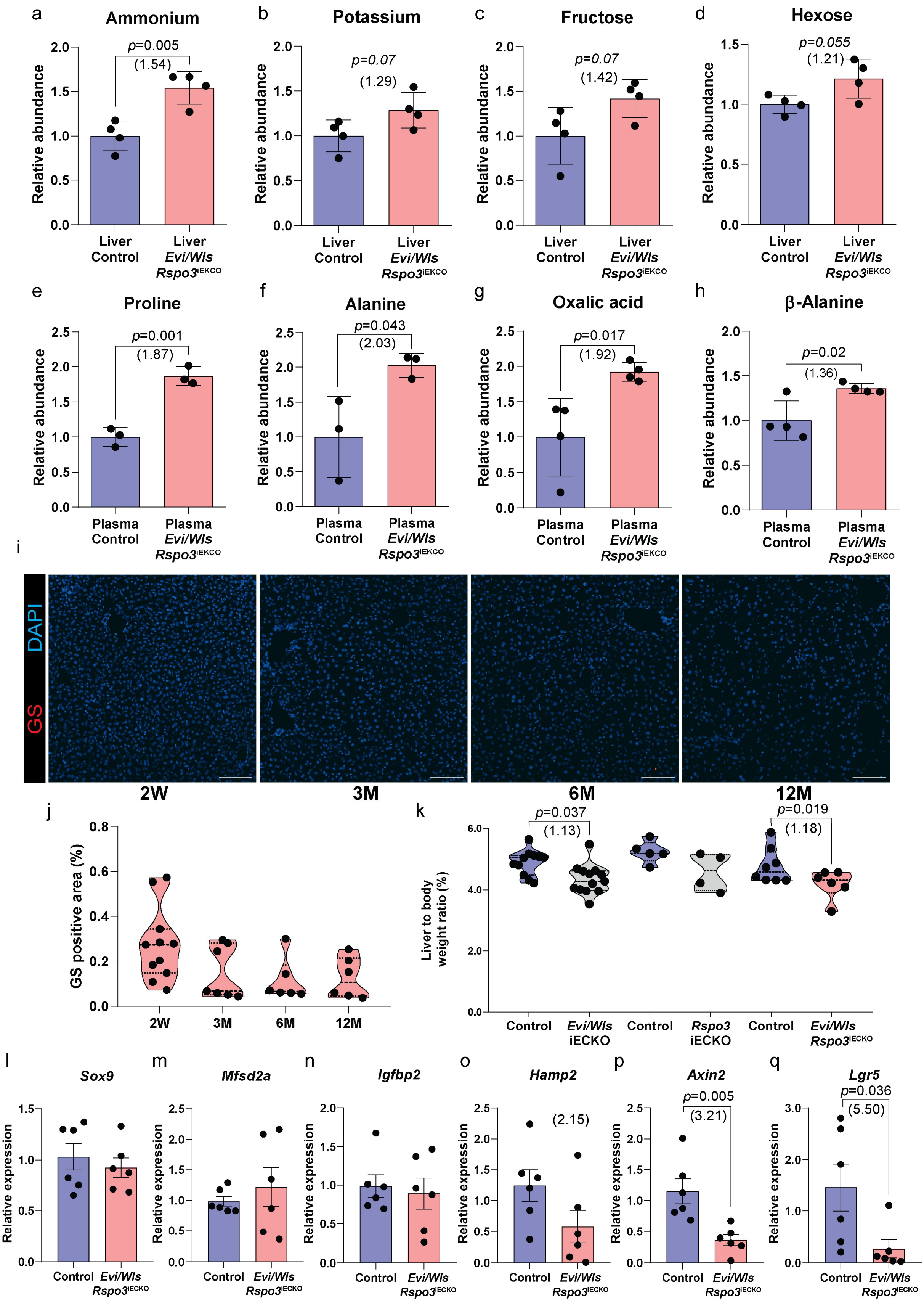
a
Ammonium
p=0.005
(1.54)
Relative abundance
Liver Control
Liver Evi/Wls Rspo3iEKCO
b
Potassium
p=0.07
(1.29)
c
Fructose
p=0.07
(1.42)
d
Hexose
p=0.055
(1.21)
e
Proline
p=0.001
(1.87)
Plasma Control
Plasma Evi/Wls Rspo3iEKCO
f
Alanine
p=0.043
(2.03)
g
Oxalic acid
p=0.017
(1.92)
h
β-Alanine
p=0.02
(1.36)
i
DAPI
GS
2W
3M
6M
12M
j
GS positive area (%)
k
Liver to body weight ratio (%)
p=0.037
(1.13)
p=0.019
(1.18)
Control
Evi/Wls iECKO
Rspo3 iECKO
Evi/Wls Rspo3iECKO
l
Sox9
m
Mfsd2a
n
Igfbp2
o
Hamp2
(2.15)
p
Axin2
p=0.005
(3.21)
q
Lgr5
p=0.036
(5.50)
Relative expression

Supplementary Fig. 4: Vascular Wnt factors control liver homeostasis.

a-**d**, Relative abundance of metabolites including ammonium, potassium, fructose and hexose in the whole liver lysate of control (n=4 mice) and Double-iECKO (n=4 mice). **e**-**h**, Relative abundance of metabolites including proline, alanine, oxalic acid, and β-alanine in the blood plasma of control (n=4 mice) and Double-iECKO (n=4 mice). **i**, Representative immunofluorescence image of GS (red) staining on liver sections from Double-iECKO from different time points after genetic recombination (2 weeks, 3 months, 6 months, and 12 months after the initial genetic recombination). Scale bars, 100µm. **j**, Corresponding quantification of GS positive area in whole liver section of Double-iECKO mice across the timeline (2w, n=11; 3m, n=8; 6m, n=6; 12m, n=6). **k**, Liver to body weight ratio of different vascular Wnt deficient mouse strains was calculated 12 months after the initial genetic recombination (control, n=11 ; Evi/Wls iECKO, n=13; control, n=5 ; Rspo3 iECKO n=4; control, n=6 ; Double-iECKO, n=6). **l-q**, Relative gene expression of periportal hepatocyte progenitor markers *Sox9* and *Mfsd2a*, midzonal hepatocyte progenitor markers *Igfbp2* and *Hamp2*, and pericentral hepatocyte progenitor markers *Lgr5* and *Axin2* in whole liver lysates in control (n=6 mice) and Double-iECKO mice (n=6 mice) 12 months post genetic recombination. () indicates fold difference. **a-h**,**j-q**, Both female and male mice were incorporated in the study. **a**-**h**, Data are presented as mean±s.d. **j**-**q**, Data are presented as mean±s.e.m. **l-q**, Gene expression was normalised to *Actb* and fold changes were determined relative to the control group. **j,** One-way ANOVA followed by Bonferroni test was performed. **a**-**h**,**k**-**q**, *p* value by two-tailed t-test is shown.

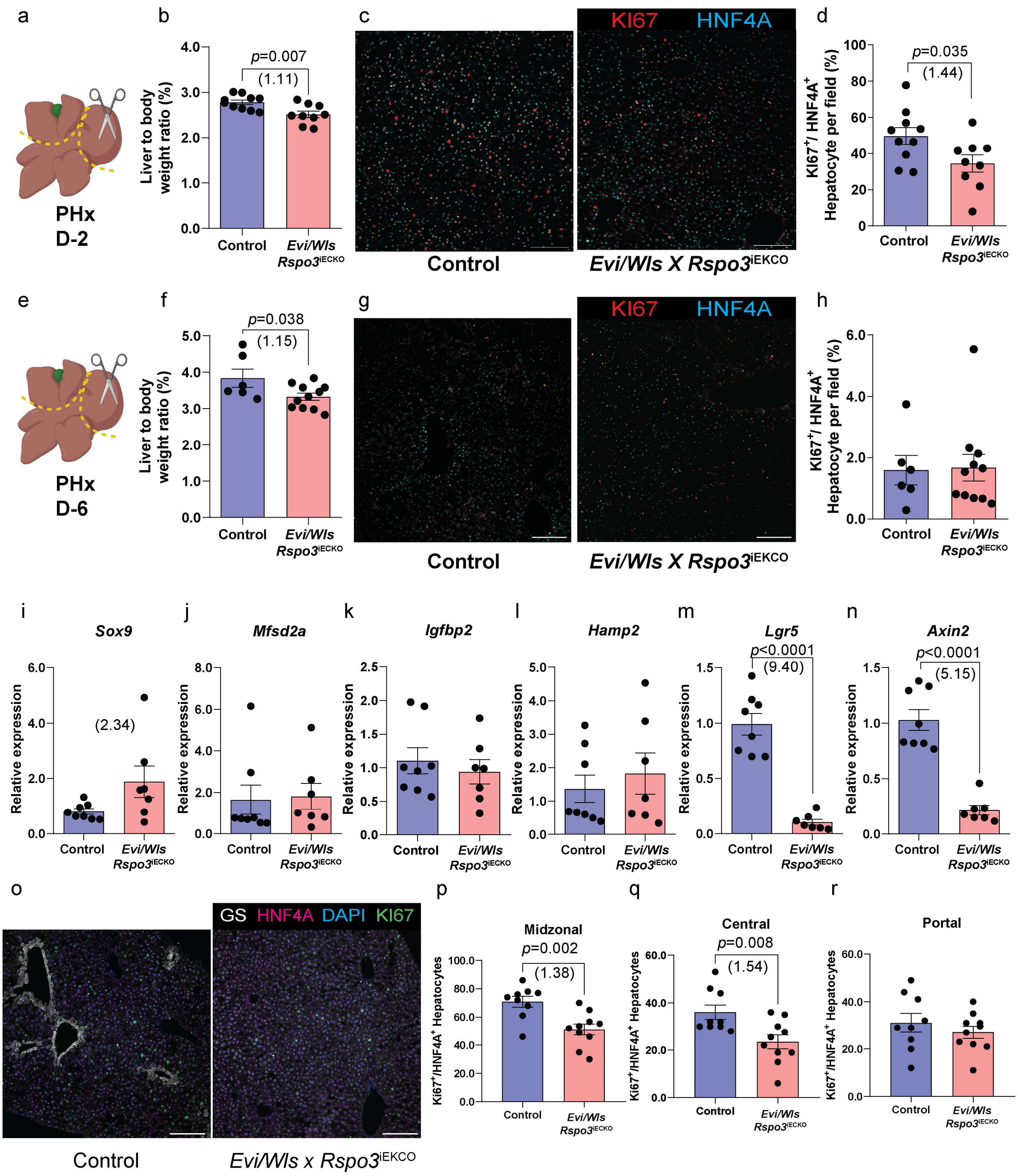

a
PHx
D-2
b
Liver to body weight ratio (%)
p=0.007
(1.11)
Control
Evi/Wls Rspo3iECKO
c
KI67 HNF4A
Control
Evi/Wls X Rspo3iECKO
d
KI67+/ HNF4A+ Hepatocyte per field (%)
p=0.035
(1.44)
e
PHx
D-6
f
p=0.038
(1.15)
g
h
i
Sox9
(2.34)
Relative expression
j
Mfsd2a
k
Igfbp2
l
Hamp2
m
Lgr5
p<0.0001
(9.40)
n
Axin2
p<0.0001
(5.15)
o
GS HNF4A DAPI KI67
Control
Evi/Wls x Rspo3iECKO
p
Midzonal
p=0.002
(1.38)
Ki67+/HNF4A+ Hepatocytes
q
Central
p=0.008
(1.54)
r
Portal

Supplementary Fig. 5: Vascular Wnt factors regulate liver regeneration.

a, $2/3^{rd}$ partial hepatectomy (PHx) was performed by resecting the lateral and median lobes of the liver. The inductive phase of liver regeneration (two days post-surgery) was monitored. Created in BioRender. Lee, KH. (2026) https://BioRender.com/c8einf4. **b**, Liver to body weight ratio was calculated two days after the PHx in Double-iECKO (n=9) and littermate control mice (n=10). **c**, Representative immunofluorescent image of cell proliferation marker KI67 (red) and hepatocyte nucleus marker HNF4A (blue) staining on the liver sections from control and Double-iECKO mice two days post-PHx. Scale bars, 100µm. **d**, Corresponding quantification of KI67 and HNF4A double positive area. **e**, Schematic overview of experimental strategy. The angiogenic phase of liver regeneration was assessed six days after $2/3^{rd}$ PHx. Created in BioRender. Lee, KH. (2026) https://https://BioRender.com/773zvmw. **f**, Liver to body weight ratio was calculated six days after the PHx in Double-iECKO (n=11) and littermate control mice (n=7). **g**, Representative immunofluorescent image of KI67 (red) and HNF4A (blue) on liver sections from Double-iECKO and control mice six days post-PHx. Scale bars, 100µm. **h**, Corresponding quantification of KI67 and HNF4A double positive area. **i-n**, Relative gene expression of periportal hepatocyte progenitor markers *Sox9* and *Mfsd2a*, midzonal hepatocyte progenitor markers *Igfbp2* and *Hamp2*, as well as pericentral hepatocyte progenitor markers *Lgr5* and *Axin2* in whole liver lysates of Double-iECKO (n=7) and control mice (n=8) two days post-PHx. **o**, Zone-specific quantification of proliferating hepatocytes. Representative immunofluorescent image of cell proliferation marker KI67 (green) and hepatocyte nucleus marker HNF4A (magenta) from control (n=9) and Double-iECKO mice (n=10) two days post-PHx. GS (grey) and DAPI (blue) served as a guide for the zonal quantification. Corresponding quantification of KI67 and HNF4A double-positive hepatocytes was plotted according to their zone: **p**, Midzonal, **q**, Central, **r**, Portal zone. Scale bars, 100µm. **b**,**d**,**f**,**h**,**i-n**,**p-r**, Both female and male mice were included in the study. **i**-**n**, Gene expression was normalised to *Actb* and the fold change was calculated relative to the control group. **b**,**d**,**f**,**h**,**i**-**n**, **p**-**r** Data are presented as mean±s.e.m. *p* value by two-tailed t-test is shown. () indicates fold difference.

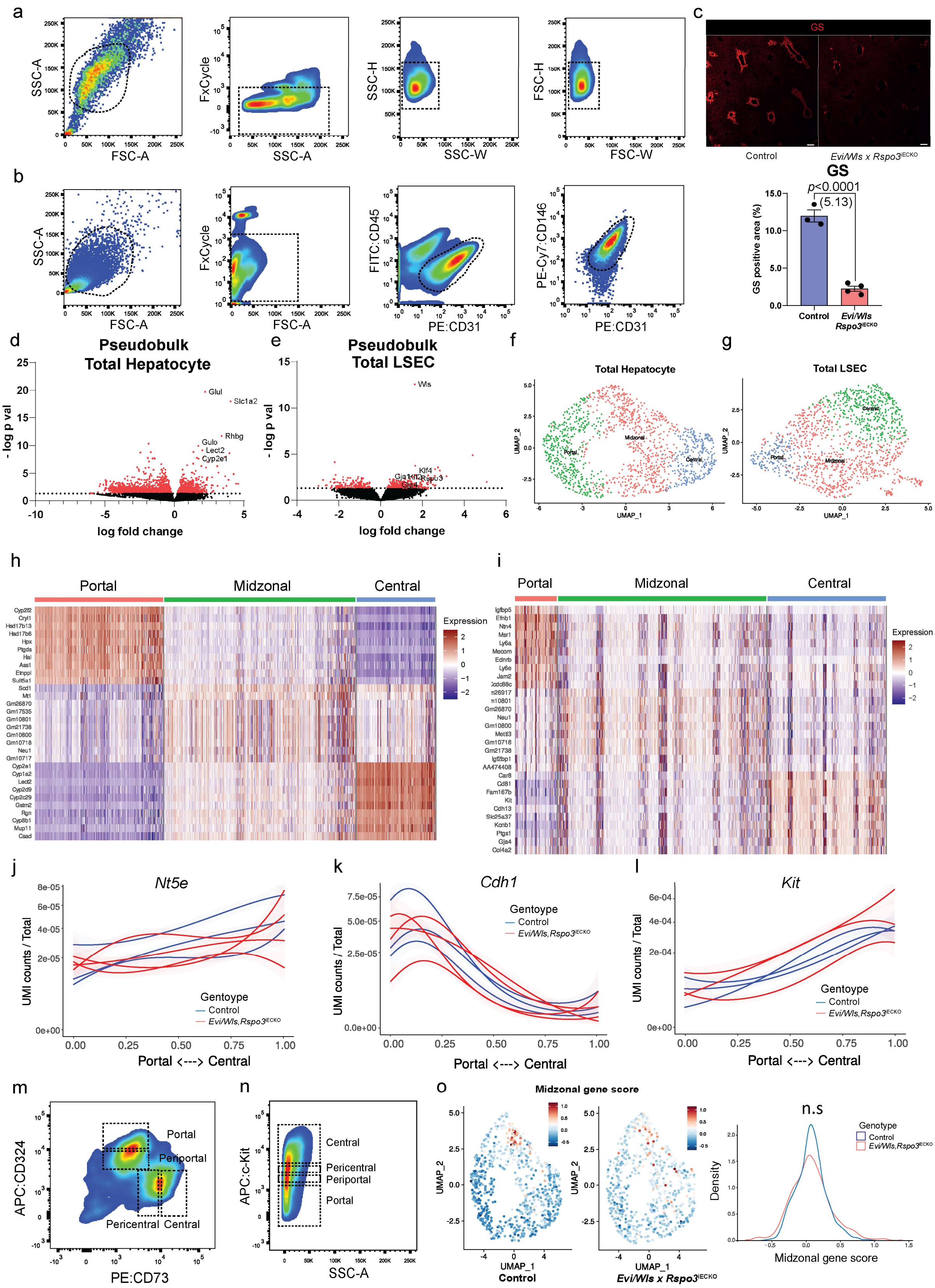
a
SSC-A
FSC-A
FxCycle
SSC-A
SSC-H
SSC-W
FSC-H
FSC-W
c
GS
Control
Evi/Wls x Rspo3iECKO
GS
p<0.0001
(5.13)
GS positive area (%)
Control
Evi/Wls Rspo3iECKO
b
SSC-A
FSC-A
FxCycle
FSC-A
FITC:CD45
PE:CD31
PE-Cy7:CD146
PE:CD31
d
Pseudobulk
Total Hepatocyte
- log p val
log fold change
Glul
Slc1a2
Rhbg
Gulo
Lect2
Cyp2e1
e
Pseudobulk
Total LSEC
- log p val
log fold change
Wls
Klf4
Rspo3
f
Total Hepatocyte
UMAP_1
UMAP_2
Portal
Midzonal
Central
g
Total LSEC
UMAP_1
UMAP_2
Portal
Midzonal
Central
h
Portal
Midzonal
Central
Expression
i
Portal
Midzonal
Central
Expression
j
Nt5e
UMI counts / Total
Gentoype
Control
Evi/Wls,Rspo3iECKO
Portal <---> Central
k
Cdh1
UMI counts / Total
Gentoype
Control
Evi/Wls,Rspo3iECKO
Portal <---> Central
l
Kit
UMI counts / Total
Gentoype
Control
Evi/Wls,Rspo3iECKO
Portal <---> Central
m
APC:CD324
PE:CD73
Portal
Periportal
Pericentral
Central
n
APC:c-Kit
SSC-A
Central
Pericentral
Periportal
Portal
o
Midzonal gene score
UMAP_1
UMAP_2
Control
Evi/Wls x Rspo3iECKO
n.s
Genotype
Control
Evi/Wls,Rspo3iECKO
Density
Midzonal gene score

Supplementary Fig. 6: Experimental design of multiomic atlas of hepatocyte and LSEC.

a, FACS sorting strategy of hepatocytes. Hepatocytes were gated based on size and viability. **b**, Gating strategy of LSEC. Viable $CD45^{-}CD31^{+}CD146^{+}$ liver ECs were collected for further analysis. **c**, Representative immunofluorescence image of GS (red) in the liver of Double iECKO (n=4) and control mouse (n=3) prior to the preprocessing. Quantification of corresponding GS expression is shown on the bottom panel. Scale bar shows 100 µm. **d**, Volcano plot of DESeq2-guided differentially expressed genes (DEGs) from pseudobulk hepatocyte dataset comparing control to the mutant hepatocyte. **e**, Volcano plot of DESeq2-guided differentially expressed genes (DEGs) from pseudobulk LSEC dataset comparing control to the mutant LSEC. **d,e,** Candidate genes are shown in the figure. Red dots represent those DEGs with p-value <0.05 (-log10>1.3). Fold change is indicated by the log2 scale. Threshold (dotted line) is set at -log10p-value>1.3. **f**, UMAP of total hepatocyte population. Clustering algorithm identified three subpopulations that corresponded to different hepatic zones. **g**, UMAP of total LSEC population. Clustering algorithm similarly identified three subpopulations that referred to the hepatic zones. **h**, Heatmap of differentially expressed hepatocyte zonation marker genes in the total hepatocyte dataset. **i**, Heatmap of differentially expressed hepatic vascular zonation marker genes. **j-l**, Spatial gene expression pattern of *Nt5e*, a known central hepatocyte marker, *Cdh1,* a known portal hepatocyte marker, and *Kit* a known central venous LSEC marker gene. Each line represents a biological replicate. Lines are coloured by the genotype. The shadowed area indicates s.e.m. **m**, FACS-based spatial-sort strategy of hepatocytes. Central vein hepatocytes were identified as $CD73^{+}$ and $CD324^{-}$. In contrast, portal vein hepatocytes were considered $CD73^{-}$ and $CD324^{+}$. Pericentral and periportal hepatocytes were characterised by intermediate expression of CD73 and CD324, respectively. **n**, Spatial-sorting strategy of LSEC. LSEC were binned into four equal portions along the c-Kit expression gradient, reflecting the different hepatic zones. **o**, Uniformed Manifold Approximation Projection (UMAP) of total hepatocyte population from control (left panel, n=3) and Double-iECKO mice (middle panel, n=4). Cells are coloured according to midzonal hepatocyte signature gene scores. Log-normalised density plot of the average midzonal hepatocyte gene score calculated for control hepatocytes (blue line) and Double-iECKO hepatocytes (red line; right panel). Average midzonal hepatocyte gene scores from individual mice were acquired and two-tailed unpaired *t*-test comparing control and Double-iECKO was performed. **c-l**,**o**, Both female and male mice were included in the study. **c**, () indicates fold difference. Data are presented as mean±s.d. p value by two-tailed t-test is shown. **d**,**e**, two-tailed Wald test and Benjamini-Hochberg were used.

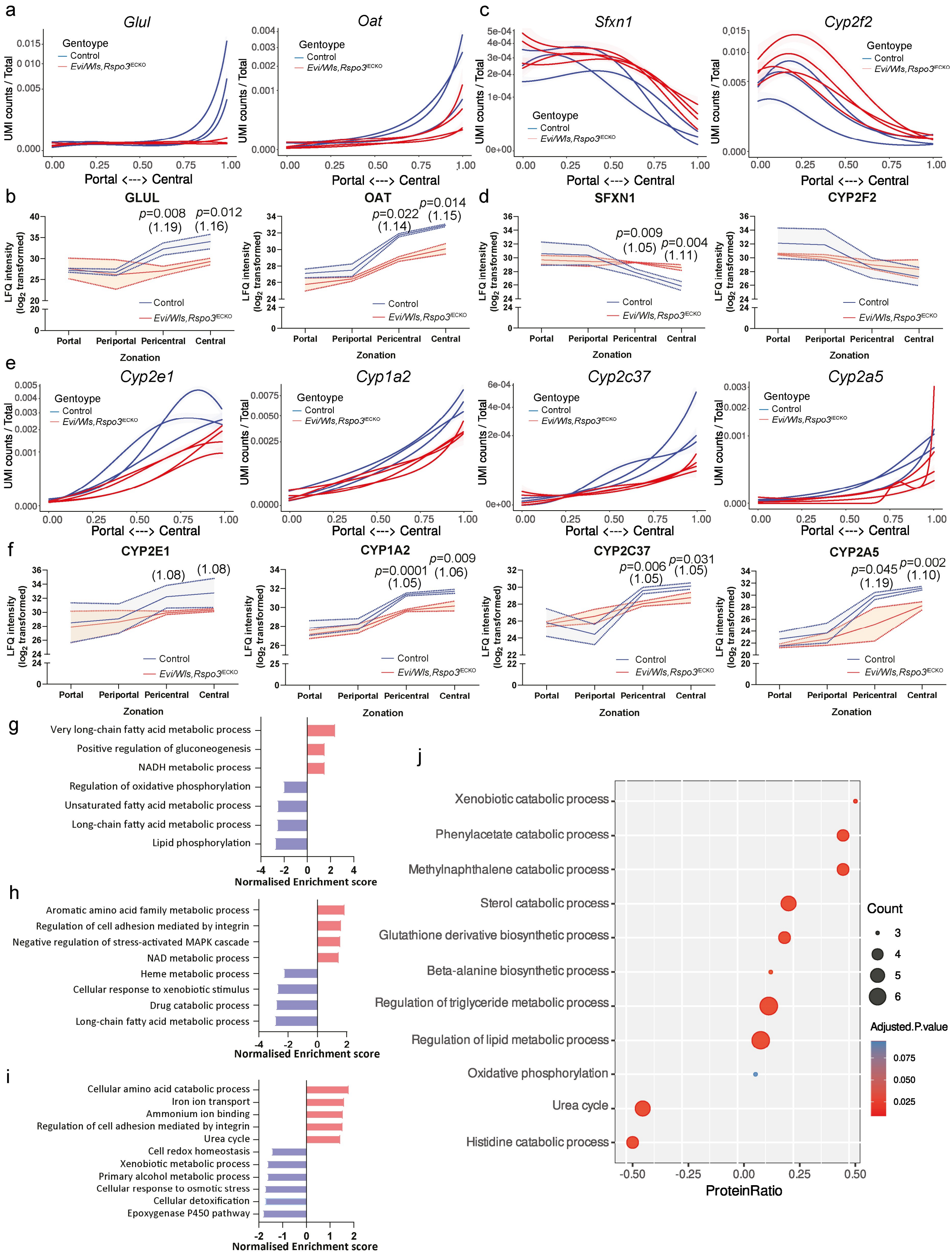
a
Glul
Oat
Gentoype
Control
Evi/Wls,Rspo3iECKO
UMI counts / Total
Portal <---> Central
b
GLUL
OAT
p=0.008 (1.19)
p=0.012 (1.16)
p=0.022 (1.14)
p=0.014 (1.15)
LFQ intensity (log2 transformed)
Portal
Periportal
Pericentral
Central
Zonation
c
Sfxn1
Cyp2f2
d
SFXN1
CYP2F2
p=0.009 (1.05)
p=0.004 (1.11)
e
Cyp2e1
Cyp1a2
Cyp2c37
Cyp2a5
f
CYP2E1
CYP1A2
CYP2C37
CYP2A5
(1.08)
(1.08)
p=0.0001 (1.05)
p=0.009 (1.06)
p=0.006 (1.05)
p=0.031 (1.05)
p=0.045 (1.19)
p=0.002 (1.10)
g
Very long-chain fatty acid metabolic process
Positive regulation of gluconeogenesis
NADH metabolic process
Regulation of oxidative phosphorylation
Unsaturated fatty acid metabolic process
Long-chain fatty acid metabolic process
Lipid phosphorylation
Normalised Enrichment score
h
Aromatic amino acid family metabolic process
Regulation of cell adhesion mediated by integrin
Negative regulation of stress-activated MAPK cascade
NAD metabolic process
Heme metabolic process
Cellular response to xenobiotic stimulus
Drug catabolic process
Long-chain fatty acid metabolic process
Normalised Enrichment score
i
Cellular amino acid catabolic process
Iron ion transport
Ammonium ion binding
Regulation of cell adhesion mediated by integrin
Urea cycle
Cell redox homeostasis
Xenobiotic metabolic process
Primary alcohol metabolic process
Cellular response to osmotic stress
Cellular detoxification
Epoxygenase P450 pathway
Normalised Enrichment score
j
Xenobiotic catabolic process
Phenylacetate catabolic process
Methylnaphthalene catabolic process
Sterol catabolic process
Glutathione derivative biosynthetic process
Beta-alanine biosynthetic process
Regulation of triglyceride metabolic process
Regulation of lipid metabolic process
Oxidative phosphorylation
Urea cycle
Histidine catabolic process
ProteinRatio
Count
Adjusted.P.value

Supplementary Fig. 7: Vascular Wnt factors are hepatocyte metabolic fate inducer.

a-**f**, Zonated mRNA and protein expression profiles of hepatocytes isolated from Double-iECKO and control mice. For mRNA data, the zonation score was calculated as described in **Fig. 2**. Shaded background indicates s.e.m. For proteomics data, proteins from spatially-sorted fractions of Double-iECKO (n=3) and littermate control (n=3) mice were quantified using log2 transformed LFQ intensity. Regression line indicates mean gene expression and the background represents s.d. Lines are coloured by the genotype. **a, b**, Spatial mRNA and protein expression profiles of pericentrally-expressed Glul and Oat. **c**,**d**, Spatial mRNA and protein expression profiles of periportal Sfxn1 and Cyp2f2. **e**,**f**, Spatial mRNA and protein expression profiles of xenobiotic metabolising enzymes, Cyp2e1, Cyp1a2, Cyp2c37, and Cyp2a5. **g**-**i**, GSEA on periportal, midzonal, and pericentral hepatocyte clusters comparing mutants and respective littermate controls. Differentially expressed genes were identified using DESeq2, comparing pseudobulks reflecting the biological replicates and spatial bin of the different genotypes. Threshold for significance was set to $p<0.05$ and $q<0.1$. Normalised enrichment score is shown for each GO term. The red bar shows upregulated, and the blue bar represents downregulated GO terms. **j**, Limma normalised GSEA enrichment analysis of proteomics data on spatially sorted central hepatocytes (n=3) and portal hepatocytes (n=3) isolated from control mice. () indicates fold difference. **a-j**, both female and male mice were considered in the study. **b**,**d**,**f**, *p* value by two-tailed t-test is shown. **g-i**, two-tailed Wald test and Benjamini-Hochberg were used. **j**, two-tailed t-test with Benjamini-Hochberg was employed.

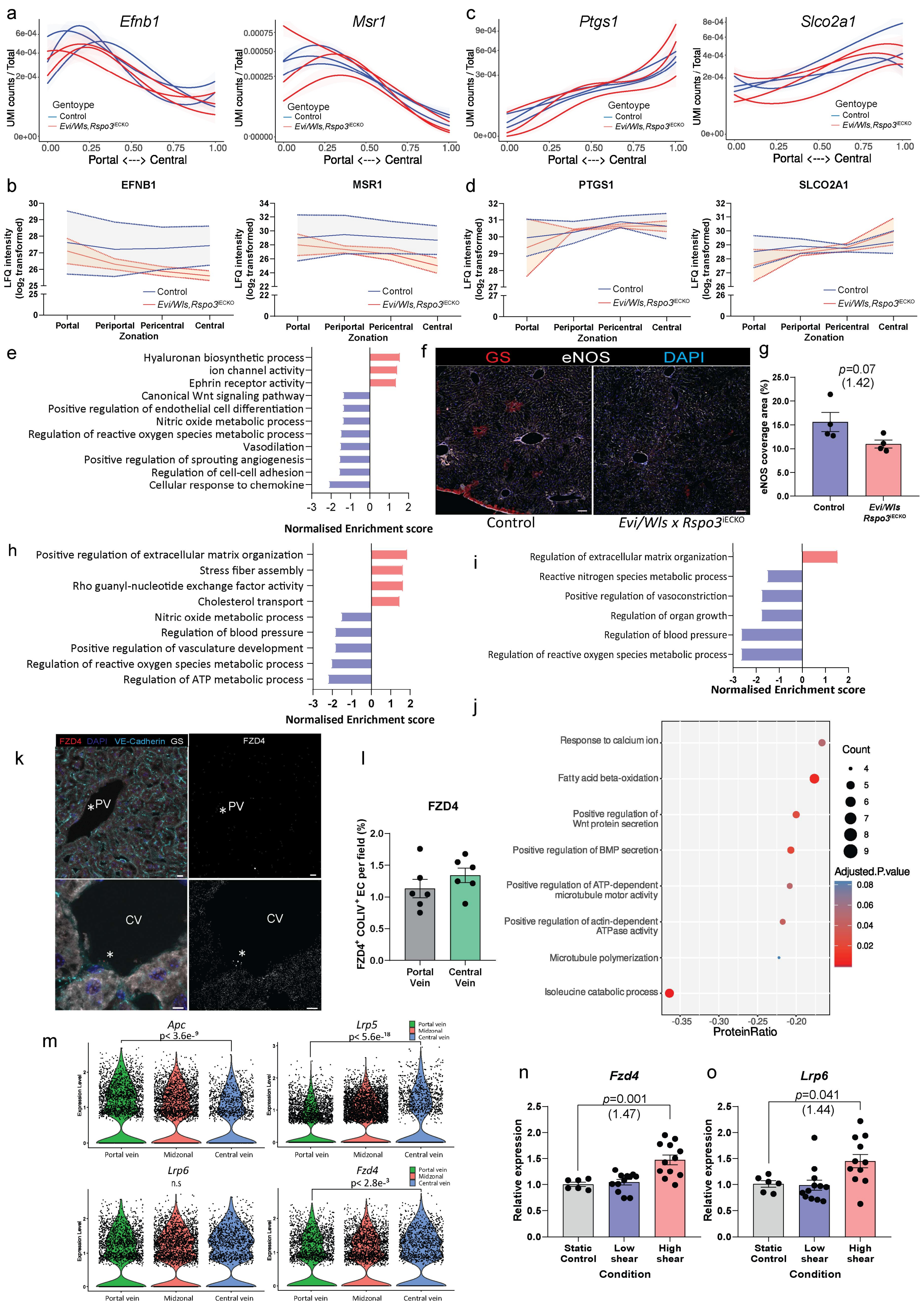

a
Efnb1
Msr1
UMI counts / Total
Portal <---> Central
Gentoype
Control
Evi/Wls,Rspo3iECKO
c
Ptgs1
Slco2a1
b
EFNB1
MSR1
LFQ intensity (log2 transformed)
Portal
Periportal
Pericentral
Central
Zonation
Control
Evi/Wls,Rspo3iECKO
d
PTGS1
SLCO2A1
e
Hyaluronan biosynthetic process
ion channel activity
Ephrin receptor activity
Canonical Wnt signaling pathway
Positive regulation of endothelial cell differentiation
Nitric oxide metabolic process
Regulation of reactive oxygen species metabolic process
Vasodilation
Positive regulation of sprouting angiogenesis
Regulation of cell-cell adhesion
Cellular response to chemokine
Normalised Enrichment score
f
GS
eNOS
DAPI
Control
Evi/Wls x Rspo3iECKO
g
p=0.07
(1.42)
eNOS coverage area (%)
Control
Evi/Wls Rspo3iECKO
h
Positive regulation of extracellular matrix organization
Stress fiber assembly
Rho guanyl-nucleotide exchange factor activity
Cholesterol transport
Nitric oxide metabolic process
Regulation of blood pressure
Positive regulation of vasculature development
Regulation of reactive oxygen species metabolic process
Regulation of ATP metabolic process
Normalised Enrichment score
i
Regulation of extracellular matrix organization
Reactive nitrogen species metabolic process
Positive regulation of vasoconstriction
Regulation of organ growth
Regulation of blood pressure
Regulation of reactive oxygen species metabolic process
Normalised Enrichment score
j
Response to calcium ion
Fatty acid beta-oxidation
Positive regulation of Wnt protein secretion
Positive regulation of BMP secretion
Positive regulation of ATP-dependent microtubule motor activity
Positive regulation of actin-dependent ATPase activity
Microtubule polymerization
Isoleucine catabolic process
ProteinRatio
Count
Adjusted.P.value
k
FZD4 DAPI VE-Cadherin GS
FZD4
PV
CV
l
FZD4
FZD4+ COLIV+ EC per field (%)
Portal Vein
Central Vein
m
Apc
p< 3.6e-9
Lrp5
p< 5.6e-18
Lrp6
n.s
Fzd4
p< 2.8e-3
Expression Level
Portal vein
Midzonal
Central vein
n
Fzd4
p=0.001
(1.47)
o
Lrp6
p=0.041
(1.44)
Relative expression
Static Control
Low shear
High shear
Condition

Supplementary Fig. 8: Spatial division of vascular Wnt signalling and its functional consequences.

a-d, Zonated mRNA and protein expression profiles of LSEC isolated from Double-iECKO and control mice as described in **Supplementary Fig. 6**. Lines are coloured by the genotype. For mRNA data, the shaded area indicates s.e.m. For proteomics data, the background represents s.d. **a, b** Spatial mRNA and protein expression profiles of periportally-expressed Efnb1 and Msr1. **c**,**d**, Spatial mRNA and protein expression profiles of pericentral Ptgs1 and Slco2a1. **e**, GSEA total LSEC comparing mutants and respective littermate controls. Differentially expressed genes were identified using DESeq2, comparing pseudobulks reflecting the biological replicates and spatial bin of the different genotypes. Threshold for significance was set to $p<0.05$ and $q<0.4$. Normalised enrichment score is shown for each GO term. The red bar shows upregulated, and the blue bar represents downregulated GO. **f**, Representative images of eNOS (grey). Central vein hepatocytes are shown with GS (red) and the cell nucleus marked by DAPI staining (blue). Scale bar shows 100µm. **g**, Quantification of eNOS positive area (control; n=4, mutant; n=4). **h**,**i**, Periportal and midzonal LSEC clusters comparing mutants and respective littermate controls. Differentially expressed genes were identified using DESeq2, comparing pseudobulks reflecting the biological replicates and spatial bin of the different genotypes. Threshold for significance was set to $p<0.05$ and $q<0.4$. Normalised enrichment score is shown for each GO term. The red bar shows upregulated, and the blue bar represents downregulated GO. **j**, Limma normalised GSEA of proteomics data on spatially sorted central vein LSEC isolated from Double-iECKO (n=3) and control (n=3) mice. **k**, Representative immunofluorescent image of FZD4 (red) co-stained with hepatic zonal marker GS (grey) and VE-Cadherin (cyan) for vasculature (left panel). FZD4 expression was further highlighted in grey (right panel). Scale bar shows 10µm. Asterisks indicate the VE-Cadherin and FZD4 double positive area. CV, central vein; PV, portal vein. **l**, Corresponding quantification of VE-Cadherin and FZD4 double positive area on liver sections of wildtype mice (n = 6). **m**, Gene expression level of *Apc*, *Lrp5*, *Lrp6* and *Fzd4* in individual LSEC of the respective spatial bins[55]. **n**, Relative gene expression of *Fzd4* and **o**, *Lrp6* in primary LSEC cultured under different shear conditions. **n**,**o**, Gene expression was normalised to *Pecam1* and fold change was calculated relative to the static control samples (static control, n=6 mice; low shear condition, n=12 mice; high shear condition, n=12 mice). () indicates fold difference. **a-e**,**g-j**,**l-o**, Both female and male mice were included in the study. **g**, Data are presented as mean±s.d. **l**,**n**,**o**, Data are presented as mean±s.e.m. **g**,**l**, p value was calculated using two-tailed t-test. **e**,**h**,**i**, two-tailed Wald test and Benjamini-Hochberg were used. **j**, two-tailed t-test with Benjamini-Hochberg was employed. **m**, Wilcoxon test was performed, **n**,**o**, One-way ANOVA followed by Bonferroni test was performed.

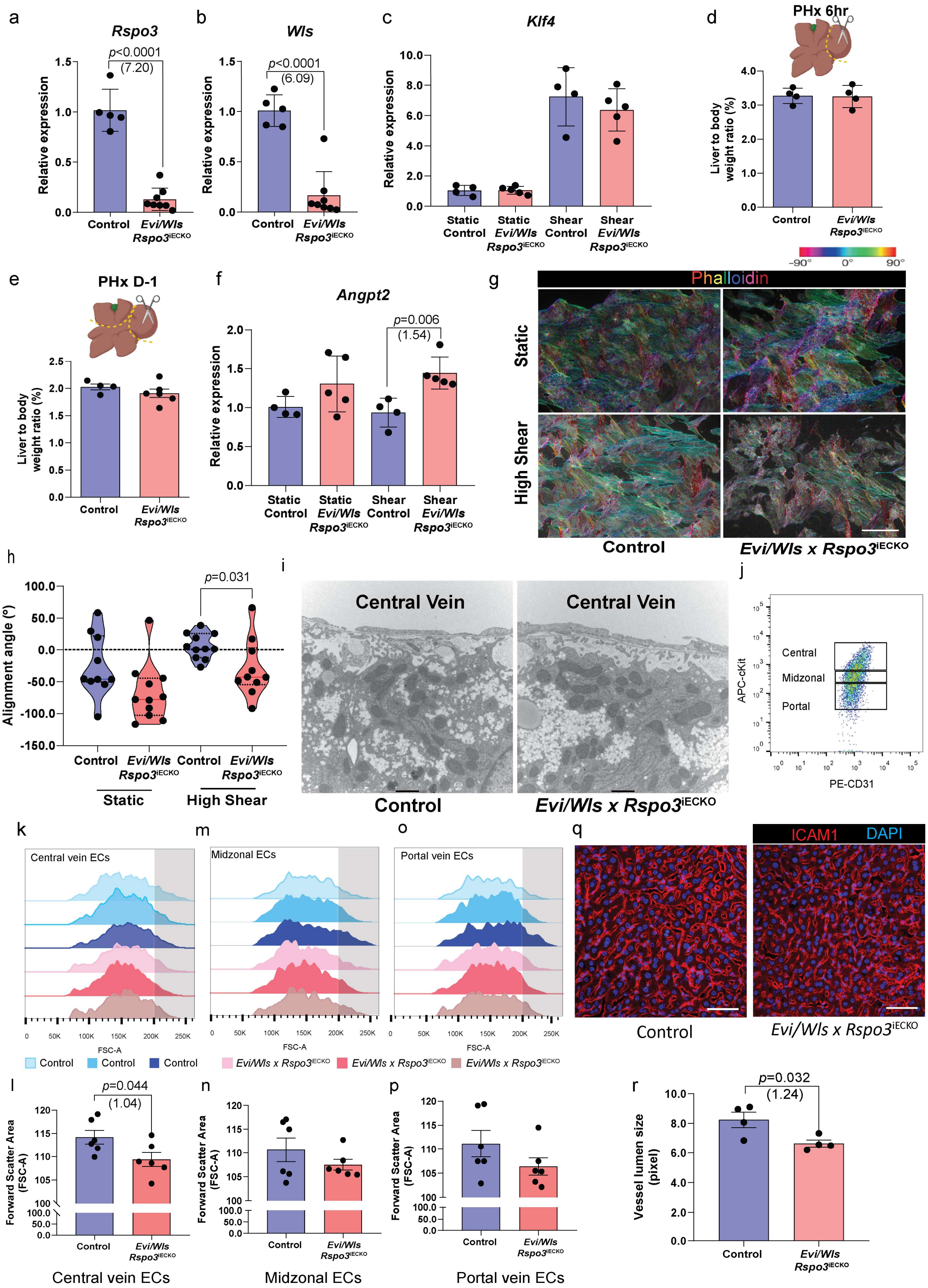

a
Rspo3
p<0.0001
(7.20)
Relative expression
Control
Evi/Wls Rspo3iECKO
b
Wls
p<0.0001
(6.09)
Relative expression
Control
Evi/Wls Rspo3iECKO
c
Klf4
Relative expression
Static Control
Static Evi/Wls Rspo3iECKO
Shear Control
Shear Evi/Wls Rspo3iECKO
d
PHx 6hr
Liver to body weight ratio (%)
Control
Evi/Wls Rspo3iECKO
e
PHx D-1
Liver to body weight ratio (%)
Control
Evi/Wls Rspo3iECKO
f
Angpt2
p=0.006
(1.54)
Relative expression
Static Control
Static Evi/Wls Rspo3iECKO
Shear Control
Shear Evi/Wls Rspo3iECKO
g
-90°
0°
90°
Phalloidin
Static
High Shear
Control
Evi/Wls x Rspo3iECKO
h
p=0.031
Alignment angle (°)
Control
Evi/Wls Rspo3iECKO
Control
Evi/Wls Rspo3iECKO
Static
High Shear
i
Central Vein
Central Vein
Control
Evi/Wls x Rspo3iECKO
j
Central
Midzonal
Portal
APC-cKit
PE-CD31
k
Central vein ECs
FSC-A
m
Midzonal ECs
FSC-A
o
Portal vein ECs
FSC-A
Control
Control
Control
Evi/Wls x Rspo3iECKO
Evi/Wls x Rspo3iECKO
Evi/Wls x Rspo3iECKO
q
ICAM1
DAPI
Control
Evi/Wls x Rspo3iECKO
l
p=0.044
(1.04)
Forward Scatter Area (FSC-A)
Control
Evi/Wls Rspo3iECKO
Central vein ECs
n
Forward Scatter Area (FSC-A)
Control
Evi/Wls Rspo3iECKO
Midzonal ECs
p
Forward Scatter Area (FSC-A)
Control
Evi/Wls Rspo3iECKO
Portal vein ECs
r
p=0.032
(1.24)
Vessel lumen size (pixel)
Control
Evi/Wls Rspo3iECKO

Supplementary Fig. 9: Aberrant vascular Wnt signalling disrupts LSEC morphology.

a, Relative gene expression of *Rspo3* and **b**, *Wls* in each primary LSEC isolated from Double-iECKO (n=8) and littermate control mice (n=5) following hydroxytamoxifen administration *in vitro*. **c**, Relative gene expression of *Klf4* in Double-iECKO and control LSEC under static or high shear conditions (static control, n=4; high shear control, n=4; static Double-iECKO, n=5; high shear Double-iECKO, n=5 mice). **d**, Liver to body weight ratio of control (n=4) and Double-iECKO mice (n=4) 6h post-PHx and **e**, 24h after PHx in control (n=4) and Double-iECKO (n=6) mice. Created in BioRender. Lee, KH. (2026) https://BioRender.com/mm0uxf9 and https://BioRender.com/zy9xw4e. **f**, Relative gene expression of *Angpt2* in LSEC isolated from Double-iECKO and control mice cultured under static or high shear conditions (static control, n=4; high shear control, n=4; static Double-iECKO, n=5; high shear Double-iECKO, n=5 mice). **g**, Phalloidin staining with subsequent *in silico* segmentation was performed to visualise shear stress fibres comparing static and peristaltic pump-induced high shear conditions of primary Double-iECKO and control LSEC using an IBIDI slide. The colour code represents fibre orientation relative to the shear axis (0°). Scale bars, 5µm. **h**, Quantification of the cell alignment angle relative to the direction of the shear (0°). Static control, n=10; shear control, n=11; static Double-iECKO, n=11, shear Double-iECKO, n=10). Each dot represents a single image. **h**, **i**, Representative transmission electron microscopy image of the lining of hepatic central veins from 3-month-old Double-iECKO (right panel) and littermate control(left panel). Scale bars, 1µm. **j**, Spatial-sorting strategy of LSEC. LSEC were binned into three equal portions along the c-Kit expression gradient, reflecting the different hepatic zones. **k**, Representative Forward scatter area (FCS-A) of central vein EC. The genotype of each mouse is indicated by the colour and below. The grey line was plotted to indicate large cell abundance (>200K$^+$ EC). **l** The geometric mean of FSC-A from the central vein subset comparing the control and Double-iECKO mouse. **m**, Representative FCS-A of midzonal EC. The genotype of each mouse is indicated by the colour and below. The grey line was plotted to indicate large cell abundance (>200K$^+$ EC). **n**, Geometric mean of FSC-A from midzonal subset comparing control and Double-iECKO mouse. **o**, Representative FCS-A of portal vein EC. The genotype of each mouse is indicated by the colour and below. The grey line was plotted to indicate large cell abundance (>200K$^+$ EC). **p**, Geometric mean of FSC-A from portal vein subset comparing control and Double-iECKO mouse. **q**, Representative images of liver sinusoids comparing Double-iECKO (n=4) and littermate control (n=4). The vessel lumen is marked by ICAM1 (red), and the nucleus is indicated by DAPI staining (blue). The scale bar shows 100µm. **r**, Quantification of average lumen size. () indicates fold difference. **a-f**,**h**,**l**,**n**,**o**,**p**,**r**, Both female and male mice were included in the study. **a**,**b**,**c**,**f**, Gene expression was normalised to *Pecam1*. Fold change was calculated relative to the control samples. **a**-**f**,**r**, Data are presented as mean±s.d. **h**,**l**,**n**,**p**, Data are presented as mean±s.e.m. **a**-**f**,**l**,**n**,**p**,**r**, *p* value by two-tailed t-test is shown.

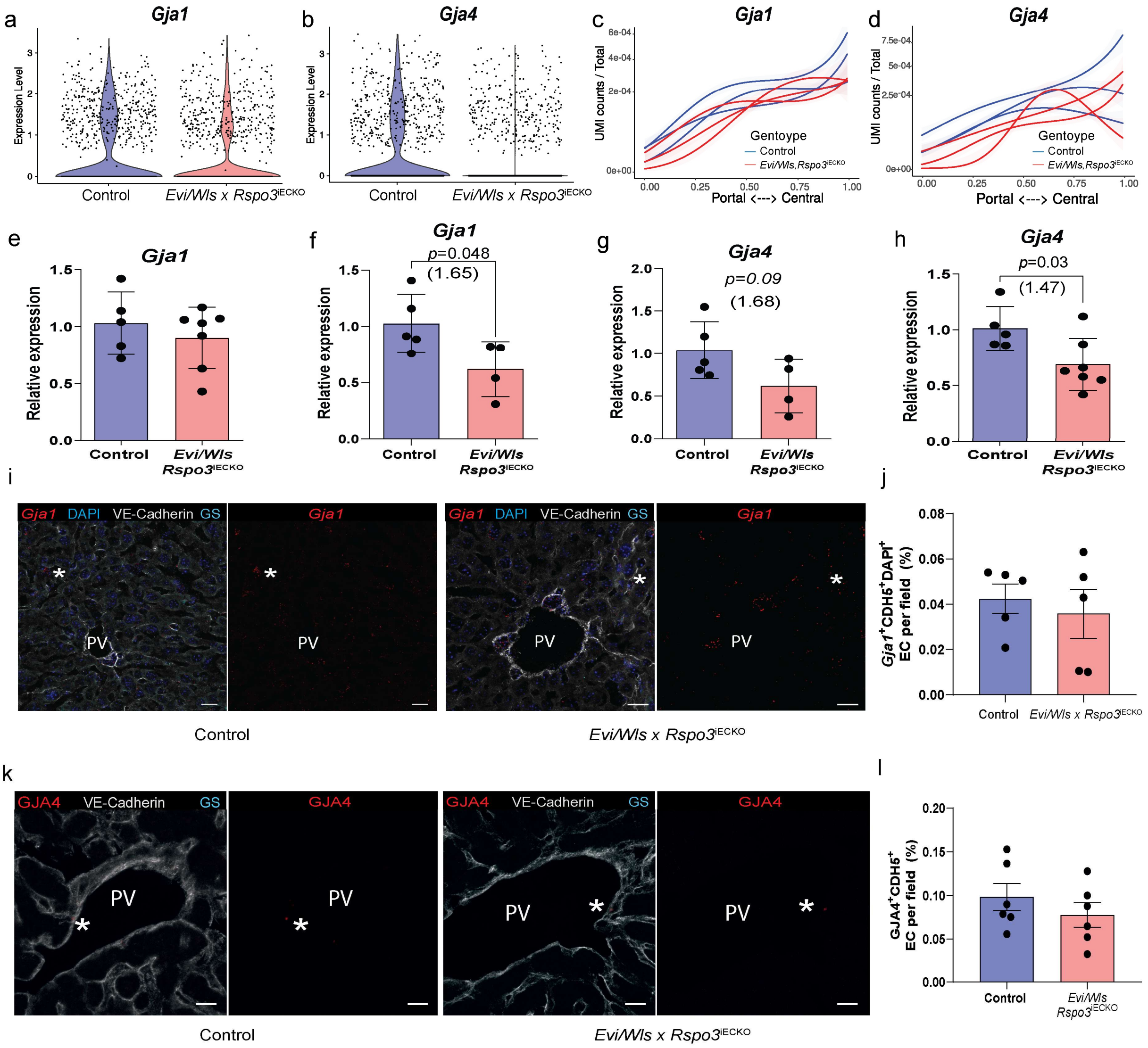

a
Gja1
Expression Level
Control
Evi/Wls x Rspo3iECKO
b
Gja4
c
Gja1
UMI counts / Total
Gentoype
Control
Evi/Wls,Rspo3iECKO
Portal <---> Central
d
Gja4
e
Gja1
Relative expression
f
p=0.048
(1.65)
g
p=0.09
(1.68)
h
p=0.03
(1.47)
i
Gja1 DAPI VE-Cadherin GS
PV
j
Gja1+CDH5+DAPI+ EC per field (%)
k
GJA4 VE-Cadherin GS
l
GJA4+CDH5+ EC per field (%)

Supplementary Fig. 10: Wnt-regulated gap junctional expression.

a, Violin plot of scRNA-seq LSEC data showing gene expression level of *Gja1* and **b**, *Gja4* in control and Double-iECKO mice. **c**, Zonation profile of *Gja1* and **d,** *Gja4* in the spatially-resolved scRNA-seq LSEC data. Zonation score was calculated based on the previously identified reference genes. Spline regression was performed to generate the spline fit. The expression level was calculated as the UMI counts for each gene per cell divided by the total UMI counts per cell. X-axis shows spatial coordination (0, portal and 1, central). Each line represents one biological replicate. Colour indicates the genotype. The shadowed area indicates s.e.m. **e**, Relative gene expression of gap junctional molecules *Gja1* and **f**, *Gja4* in MACS-bead sorted LSEC from control (n=5) and Double-iECKO mice (n=4). **g**, Relative gene expression of *Gja1* and **h**, *Gja4* in cultured primary LSEC isolated from control and Double-iECKO mice after induction of genetic recombination *in vitro* (control, n=5; Double-iECKO, n=7). **i**, Representative in situ hybridisation image of *Gja1* (red) on DAPI (blue) co-stained with hepatic zonal marker GS (cyan) and VE-Cadherin (grey) for vasculature at the portal vein. *Gja1* expression was further highlighted in red (right panel). *Gja1* expression between the mutant (n=5) and littermate control (n=5) was compared. Asterisks indicate the *Gja1*, VE-Cadherin, and DAPI triple positive area. Scale bar indicates 10µm. **j**, Corresponding quantification of *Gja1*, VE-Cadherin, and DAPI triple positive area on liver sections of control mice and mutant mice. **k**, Representative immunofluorescence image of GJA4 (red) co-stained with hepatic zonal marker GS (cyan) and VE-Cadherin (grey) for vasculature at the portal vein. GJA4 expression was further highlighted in red (right panel). GJA4 expression between the mutant (n=6) and littermate control (n=6) was compared. Asterisks indicate the GJA4 and VE-Cadherin double positive area. Scale bar indicates 10µm. **l**, Corresponding quantification of GJA4 and VE-Cadherin double positive area on liver sections of control mice and mutant mice. **a-h**,**j**,**l**, Both female and male mice were included in the study. **e**-**h**,**j**, Data are presented as mean±s.d. **l**, Data are presented as mean±s.e.m. **e**-**h**,**j**,**l**, *p* value by two-tailed t-test is shown.

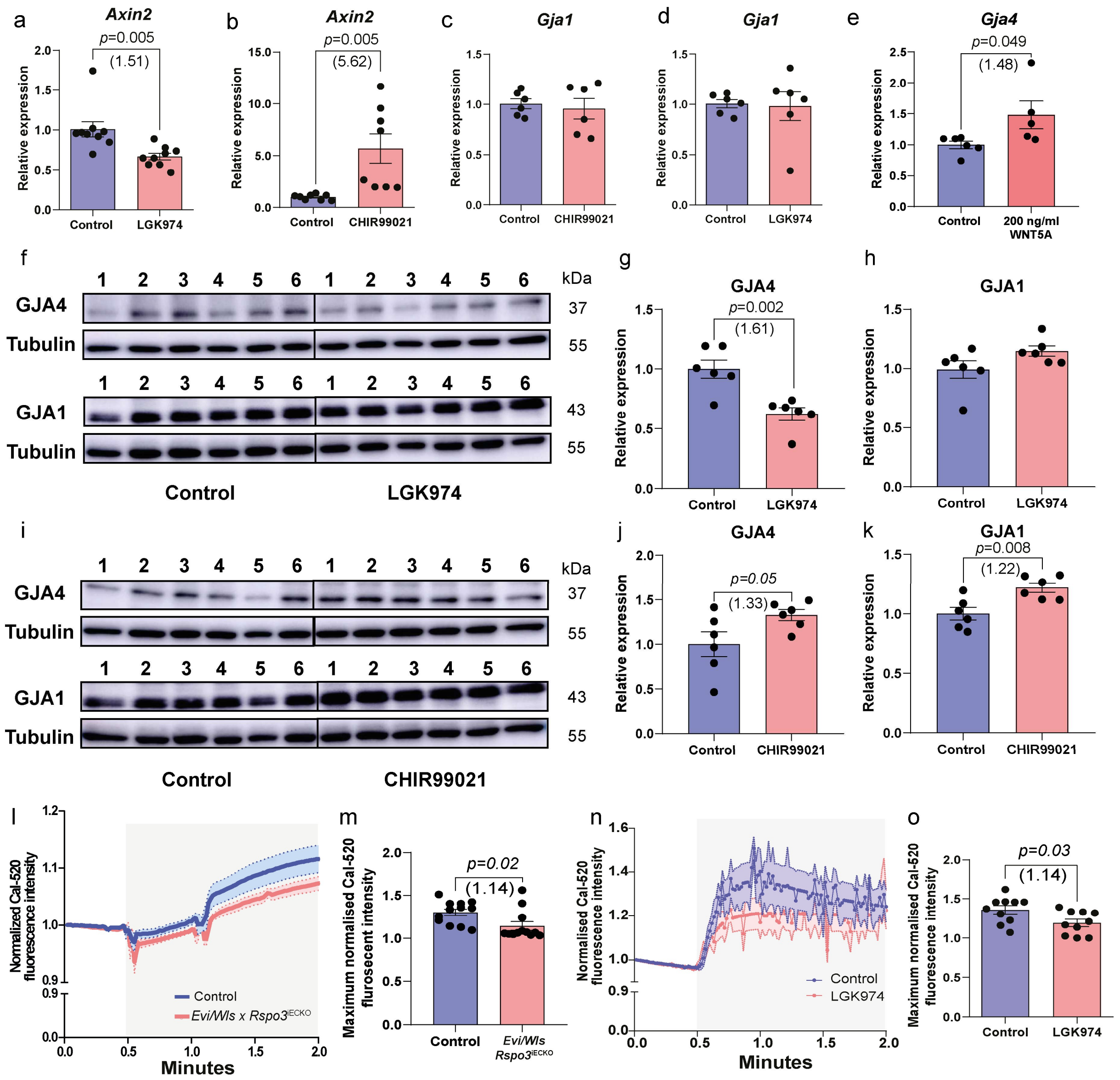

a
Axin2
p=0.005
(1.51)
Relative expression
Control
LGK974
b
Axin2
p=0.005
(5.62)
Control
CHIR99021
c
Gja1
Control
CHIR99021
d
Gja1
Control
LGK974
e
Gja4
p=0.049
(1.48)
Control
200 ng/ml WNT5A
f
GJA4
Tubulin
GJA1
kDa
37
55
43
Control
LGK974
g
GJA4
p=0.002
(1.61)
h
GJA1
i
Control
CHIR99021
j
GJA4
p=0.05
(1.33)
k
GJA1
p=0.008
(1.22)
l
Normalized Cal-520 fluorescence intensity
Control
Evi/Wls x Rspo3iECKO
Minutes
m
Maximum normalised Cal-520 flurosecent intensity
p=0.02
(1.14)
Control
Evi/Wls Rspo3iECKO
n
Normalised Cal-520 fluorescence intensity
Control
LGK974
Minutes
o
Maximum normalised Cal-520 fluorescence intensity
p=0.03
(1.14)
Control
LGK974

Supplementary Fig. 11: Wnt-regulated gap junctional expression and function *in vitro*.

a, Relative gene expression of Wnt-response gene *Axin2* after LGK974 (n=9), and **b**, CHIR99021 (n=8) treatment in primary LSEC isolated from wildtype mice. **c**, Relative gene expression of *Gja1* after LGK974 (n=6) and **d**, CHIR99021 treatment (n=6) in primary LSEC isolated from wildtype mice. **e**, *Gja4* mRNA expression in primary LSEC isolated from wildtype mice treated with non-canonical WNT5A (n=6). **f**, Immunoblotting analysis of gap junctional molecules on primary LSEC treated either with LGK974 (n=6) or vehicle control (n=6). GJA1, GJA4, and their corresponding tubulin expression were blotted. **g**, Relative protein abundance of GJA4 and **h**, GJA1 after the Wnt inhibition. **i**, Immunoblotting analysis of gap junctional molecules on primary LSEC treated either with CHIR99021 (n=6) or vehicle control (n=6). GJA1, GJA4, and their corresponding tubulin expression were blotted. **j**, Relative protein abundance of GJA4 and **k**, GJA1 after the Wnt activation. **l**, Live cell calcium tracing of Wnt-deficient primary LSEC (n=4, 3 replicates) and control LSEC (n=4 mice, 3 replicates) under orbital shaker-derived high laminar shear. **m**, Quantification of maximum normalised fluorescence intensity ratio. **n**, Live cell calcium imaging of primary LSEC isolated from wildtype mice and treated with LGK974 (n=10) or vehicle control (n=10). **o**, Corresponding quantification of maximum normalised fluorescence intensity ratio. () indicates fold difference. **a-o**, Both female and male mice were incorporated in the study. **a-e**, Gene expression was normalised to *Pecam1* expression. Fold change was calculated relative to the respective control. **m**,**o**, Primary LSEC were incubated with calcium indicator, Cal-520, for 30 minutes prior to image acquisition. Fluorescence intensity was normalised to the resting state. Grey background indicates the timeframe of calcium administration. **a-e**,**g**,**h**,**j**,**k**,**m**,**o**, Data are presented as mean±s.e.m. and *p* value by two-tailed t-test is shown.

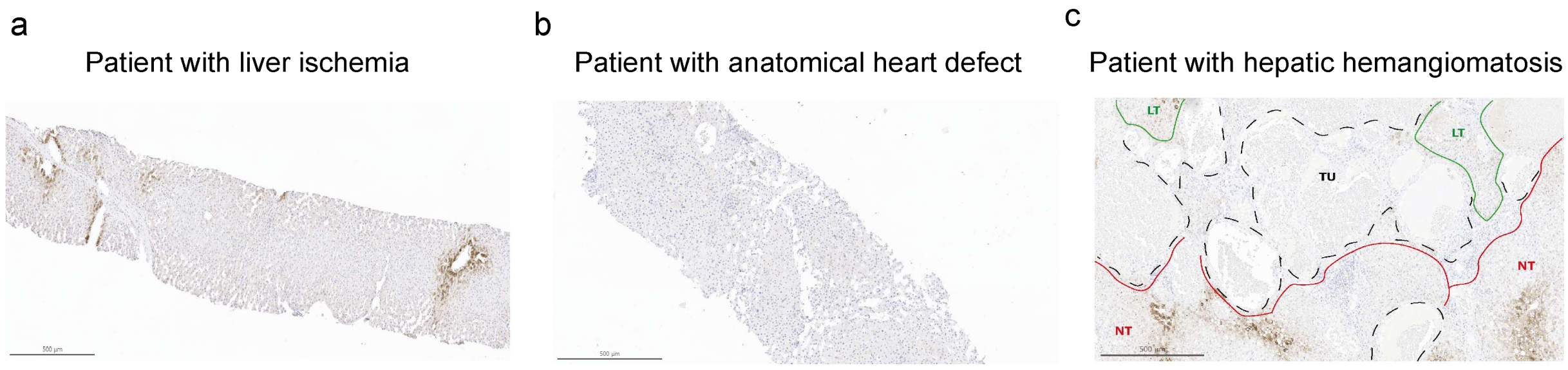

Supplementary Fig. 12: Vascular mechanotransduction concept of liver function in clinical settings.

a-c, Immunostaining was performed on clinical specimens using GS antibody. **a**, Sample from a 74-year-old male patient with a history of liver transplantation and repeated events of liver ischemia. **b,** Sample from a 17-year-old female with a history of complex anatomical heart defect and life-long chronic liver congestion. **c,** Sample from a 42-year-old female with a history of hepatic hemangiomatosis. The labels indicate tumour region (TU), loss of GS in the surrounding liver tissue (LT), and retained GS signal in the periphery (NT). Scale bars, 500µm.

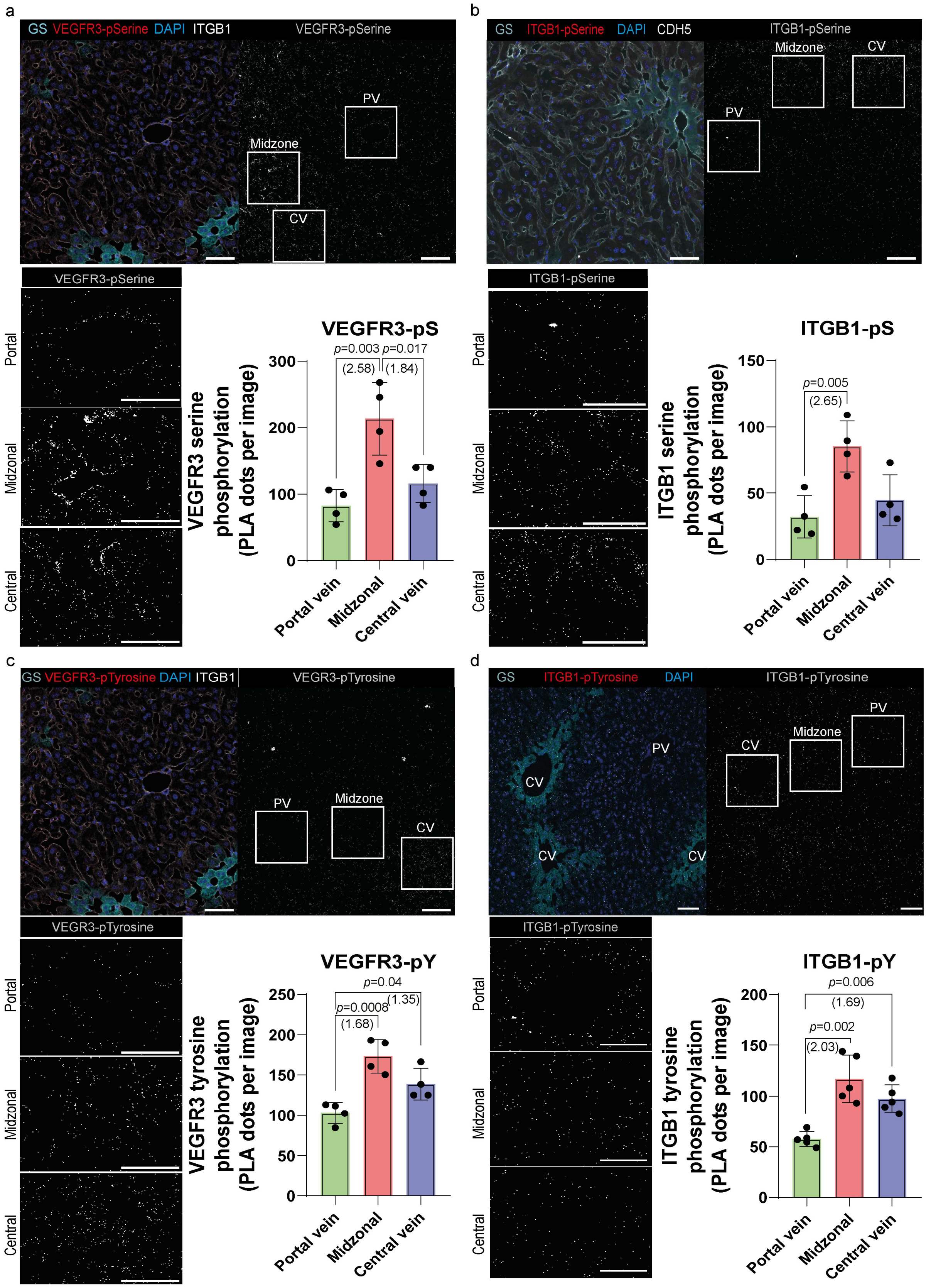
a
GS VEGFR3-pSerine DAPI ITGB1
VEGFR3-pSerine
PV
Midzone
CV
VEGFR3-pSerine
Portal
Midzonal
Central
VEGFR3-pS
VEGFR3 serine phosphorylation (PLA dots per image)
p=0.003 p=0.017
(2.58) (1.84)
300
200
100
0
Portal vein
Midzonal
Central vein
b
GS ITGB1-pSerine DAPI CDH5
ITGB1-pSerine
Midzone
CV
PV
ITGB1-pSerine
Portal
Midzonal
Central
ITGB1-pS
ITGB1 serine phosphorylation (PLA dots per image)
p=0.005
(2.65)
150
100
50
0
Portal vein
Midzonal
Central vein
c
GS VEGFR3-pTyrosine DAPI ITGB1
VEGR3-pTyrosine
PV
Midzone
CV
VEGR3-pTyrosine
Portal
Midzonal
Central
VEGFR3-pY
VEGFR3 tyrosine phosphorylation (PLA dots per image)
p=0.04
p=0.0008 (1.35)
(1.68)
250
200
150
100
50
0
Portal vein
Midzonal
Central vein
d
GS ITGB1-pTyrosine DAPI
ITGB1-pTyrosine
PV
CV
CV
CV
PV
Midzone
CV
ITGB1-pTyrosine
Portal
Midzonal
Central
ITGB1-pY
ITGB1 tyrosine phosphorylation (PLA dots per image)
p=0.006
(1.69)
p=0.002
(2.03)
200
150
100
50
0
Portal vein
Midzonal
Central vein

Supplementary Fig. 13: Zone-specific activation of hepatic vascular mechanosensors.

a, Proximity ligation assay (PLA) based measurement of VEGFR3 and phosphoserine interaction in wildtype mice (n = 4). Representative PLA image of VEGFR3-phosphoserine (red) co-stained with hepatic zonal marker GS (cyan) and ITGB1 (grey) for vasculature (upper left). The VEGFR3-phosphoserine interaction was further highlighted in grey (upper right). Representative image of zone-specific VEGFR3-phosphoserine interactions. The zoomed interaction was visualised (bottom left) and the corresponding quantification is shown (bottom right). The scale bar shows 10 µm. **b**, PLA assay on ITGB1 and phosphoserine interaction in wildtype mice (n = 4). Representative PLA image of ITGB1-phosphoserine (red) co-stained with hepatic zonal marker GS (cyan) and VE-cadherin (grey) for vasculature (upper left). The ITGB1-phosphoserine interaction was further highlighted in grey (upper right). Representative image of zone-specific ITGB1-phosphoserine interactions. The zoomed interaction was visualised (bottom left) and the corresponding quantification is shown (bottom right). The scale bar shows 10 µm. **c**, PLA assay on VEGFR3 and phosphotyrosine interaction in wildtype mice (n = 4). Representative PLA image of VEGFR3-phosphotyrosine (red) co-stained with hepatic zonal marker GS (cyan) and ITGB1 (grey) for vasculature (upper left). The VEGFR3-phosphotyrosine interaction was further highlighted in grey (upper right). Representative image of zone-specific VEGFR3-phosphotyrosine interactions. The zoomed interaction was visualised (bottom left) and the corresponding quantification is shown (bottom right). The scale bar shows 10 µm. **d**, PLA assay on ITGB1 and phosphotyrosine interaction in wildtype mice (n = 5). Representative PLA image of ITGB1-phosphotyrosine (red) co-stained with hepatic zonal marker GS (cyan) (upper left). ITGB1-phosphotyrosine interactions were further highlighted in grey (upper right). Representative image of zone-specific ITGB1-phosphotyrosine interactions. zoomed The interaction was visualised (bottom left) and the corresponding quantification is shown (bottom right). The scale bar shows 10 µm. () indicates fold difference. **a-d**, The data are presented as mean±s.d. *p* value by two-tailed t-test is shown. pS and pY indicate phosphoserine and phosphotyrosine. Both female and male mice were included in the study.

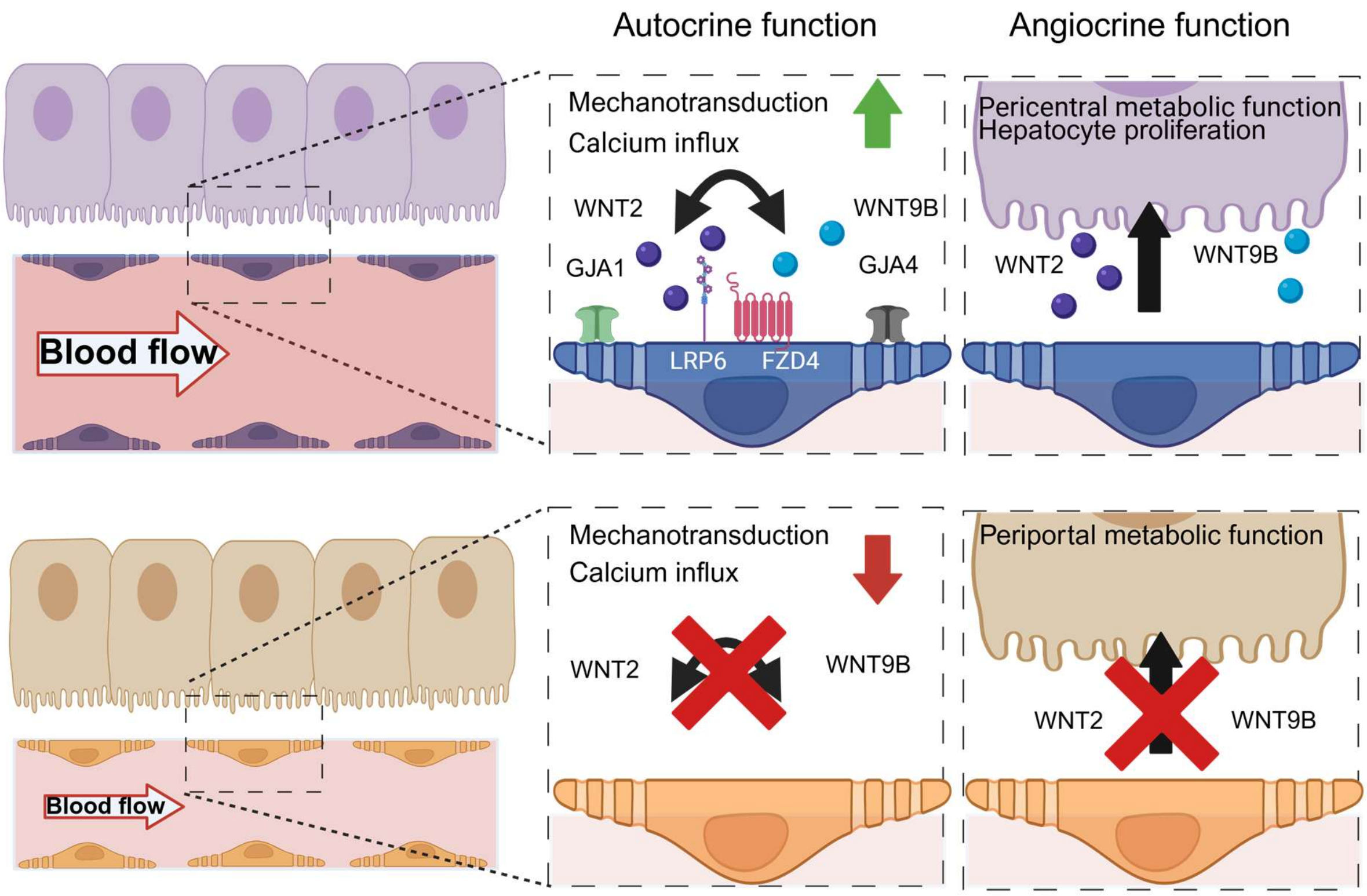

Supplementary Fig. 14: Biomechanically-induced vascular Wnt signalling regulates liver function.

Model of haemodynamically activated vascular Wnt signalling orchestrating liver homeostasis. Central vein LSEC are exposed to unique haemodynamic stimuli, which stimulate the expression of the Wnt-signalling complex consisting of Wnt2, Wnt9b, Fzd4, and Lrp6. Vascular Wnt factors enforce central vein LSEC function by regulating the expression of gap junctional molecules that mediate calcium homeostasis and coordinating LSEC morphology, including LSEC size and LSEC orientation. Concurrently, zonated vascular Wnt-signalling sustains the spatial division of labour in the liver by instructing and maintaining the metabolic fate of pericentral hepatocytes in an angiocrine manner. In contrast, perturbances in blood perfusion directly affect the expression of vascular Wnt and overall zonated liver function. Created in BioRender. Lee, KH. (2026) https://BioRender.com/6fy59x9.

.
