# Supplementary material for: Haemodynamic control of zonated liver function via instructive vascular Wnt signalling
Source: Nat Commun. 2026 Aug 22;17:8821. doi: 10.1038/s41467-026-76966-7 (PMC13499805; doi:10.1038/s41467-026-76966-7)
Supplement: Supplementary file 2 [file 41467_2026_76966_MOESM2_ESM.pdf]

Description of Additional Supplementary Files

Title: **Supplementary Data 1**
Description:

Sheet 1:
Differentially expressed genes comparing hepatocytes from the double mutant and control mice

Sheet 2:
Differentially expressed genes comparing LSEC from the double mutant and control mice

Pseudobulk samples were extracted from scRNA-seq data and DEGs between genotypes were calculated using DESeq2. Details can be found in "Single-cell RNA-seq analysis" of the method.

Title: **Supplementary Data 2**
Description:

Sheet 1:
Differentially expressed genes comparing hepatocytes from the double mutant and control mice across different zones
Sheet 2:
Differentially expressed genes comparing LSEC from the double mutant and control mice across different zones

Zonation coordinates are assigned as (0,2=central, 0,5=midzonal, 0,8=portal).
Wald testing was used for statistical analysis. Details can be found in "Assignment of zonation position", "Smoothing gene expression along zonation", and "Ranked position score" of the method.

Title: **Supplementary Data 3**
Description:

Sheet 1:
Log2-transformed LFQ intensity of hepatocytes from the double mutant and control mice
Sheet 2:
Log2-transformed LFQ intensity of LSEC from the double mutant and control mice

Details can be found in "Analysis of raw MS data" of the method.

Title: **Supplementary Data 4**
Description:

Sheet 1:
Metabolite Reporting Checklist

Sheet 2:

Mass Spectrometry-guided metabolite screening of blood plasma and whole liver lysates from the double mutant and control mice
