# Supplementary material for: Haemodynamic control of zonated liver function via instructive vascular Wnt signalling
Source: Nat Commun. 2026 Aug 22;17:8821. doi: 10.1038/s41467-026-76966-7 (PMC13499805; doi:10.1038/s41467-026-76966-7)
Supplement: Supplementary file 3 [file 41467_2026_76966_MOESM7_ESM.pdf]

Corresponding author(s): Ki Hong Lee
Hellmut G. Augustin

Last updated by author(s): Aug 11, 2026

Reporting Summary

Nature Portfolio wishes to improve the reproducibility of the work that we publish. This form provides structure for consistency and transparency in reporting. For further information on Nature Portfolio policies, see our Editorial Policies and the Editorial Policy Checklist.

Statistics

For all statistical analyses, confirm that the following items are present in the figure legend, table legend, main text, or Methods section.

n/a	Confirmed	
☐	☒	The exact sample size (*n*) for each experimental group/condition, given as a discrete number and unit of measurement
☐	☒	A statement on whether measurements were taken from distinct samples or whether the same sample was measured repeatedly
☐	☒	The statistical test(s) used AND whether they are one- or two-sided<br>*Only common tests should be described solely by name; describe more complex techniques in the Methods section.*
☒	☐	A description of all covariates tested
☐	☒	A description of any assumptions or corrections, such as tests of normality and adjustment for multiple comparisons
☐	☒	A full description of the statistical parameters including central tendency (e.g. means) or other basic estimates (e.g. regression coefficient) AND variation (e.g. standard deviation) or associated estimates of uncertainty (e.g. confidence intervals)
☐	☒	For null hypothesis testing, the test statistic (e.g. *F*, *t*, *r*) with confidence intervals, effect sizes, degrees of freedom and *P* value noted<br>*Give P values as exact values whenever suitable.*
☒	☐	For Bayesian analysis, information on the choice of priors and Markov chain Monte Carlo settings
☒	☐	For hierarchical and complex designs, identification of the appropriate level for tests and full reporting of outcomes
☒	☐	Estimates of effect sizes (e.g. Cohen's *d*, Pearson's *r*), indicating how they were calculated

Our web collection on statistics for biologists contains articles on many of the points above.

Software and code

Policy information about availability of computer code

Data collection	FACS data was generated using FACSAria and FACSFusion sorting platform (BD Bioscience) and recorded with BD FACSDIVA v8 (BD Bioscience) software. Imaging was performed using Zeiss LSM980, Zeiss Widefield Axio Cellobserver (Zeiss), Leica TCS SPS II (Leica), and Leica TCS SP8 (Leica) confocal microscope and imaging data were collected with ZEN v2.6 (Zeiss), ZEN Blue (Zeiss), Leica LAS AF v2.7.3 (Leica), and Leica LAS X v3.5.5 (Leica) software, respectively.<br>Electron microscope was performed using Zeiss EM 910 (Zeiss).<br>qRT-PCRwas performed using StepOnePlus (Applied Biosystems) and data were recorded with Stepone software v2.I (Applied Biosystems).<br>Metabolite screening was performed using GC/MS-QP2010 Plus (Shimadzu) and recorded with GCMS solution software (Shimadzu).<br>Unbiased proteomic screening was performed and recorded using EASY nanoLC 1200 System (Thermo Scientific) and QE- HF mass spectrometer (Thermo Scientific). scRNA-seq libraries were sequenced on NextSeq5SO (IIlumina) using Paired-End 7Sbp High Output kit.<br>Immunoblots were acquired using Amersham Imager 600 (GE Healthcare).
Data analysis	FACS samples were analysed with FlowJo v10 (BD Bioscience). Data analysis, statistical testing and visualisation were performed in Graphpad Prism v8 (GraphPad), R v. 3.4.3 (R Foundation for Statistical Computing) and Rstudio vI.2.5042 (Posit).<br>Confocal images were analysed using Fiji/ImageJ vI.53t and v1.54g (NIH). Metabolomic data were analysed using GCMS solution software (Shimadzu). Proteomic data were processed using Maxquant vl.6.10.43 (Max Planck Institute of Biochemistry) and pathway analysis was performed using LFQ-Anaylst v 1.2.4 (Monash University). For scRNA-seq experiments, data were aligned and processed with Subread aligner vI.6.5 and Samtools vI.0.9. The code for processing CEL-seq2 data can be found under https://github.com/simon-anders/. The data analysis was performed using Seurat v3 packages and plotted with ggplot2 v 3.3.4. Differential gene expression was assessed using DESeq2 and gene set enrichment analysis was performed using clusterProfiler v4.0.I. For data visualizatoin, volcano plot, dot plots, and violin plots were mployed from Seurat v3 an ggplot2 v 3.3.4. For modelling of liver zonation in silico, spline regression was applied for smoothing gene

expression using MASS package. Custom code and scripts for scRNA-seq analyses are available at https://github.com/anders-biostat/epatocytes-zonation/.

For manuscripts utilizing custom algorithms or software that are central to the research but not yet described in published literature, software must be made available to editors and reviewers. We strongly encourage code deposition in a community repository (e.g. GitHub). See the Nature Portfolio guidelines for submitting code & software for further information.

Data

Policy information about availability of data

All manuscripts must include a data availability statement. This statement should provide the following information, where applicable:

- Accession codes, unique identifiers, or web links for publicly available datasets
- A description of any restrictions on data availability
- For clinical datasets or third party data, please ensure that the statement adheres to our policy

Raw sequencing FASTQ files and scRNA-seq count matrices can be found in GSE230663. Raw mass spectrometry proteomic source files are available on MSV000102526 (PXD081294). The LFQ-intensity counts are available in the manuscript (Supplementary Table 3).
All non-standard code can be found under the following link:
Custom code and scripts for pre-processing of CEL-seq2 are available at https://github.com/simon-anders/
Custom code and scripts for scRNA-seq analyses are available at https://github.com/anders-biostat/hepatocytes-zonation/
An interactive webtool visualising the spatially-resolved scRNA-seq data can be accessed under: https://papagei.bioquant.uni-heidelberg.de/kiapp/

Research involving human participants, their data, or biological material

Policy information about studies with human participants or human data. See also policy information about sex, gender (identity/presentation), and sexual orientation and race, ethnicity and racism.

Reporting on sex and gender	To follow 3R, both male and female mice were incorporated in the study.
Reporting on race, ethnicity, or other socially relevant groupings	*Please specify the socially constructed or socially relevant categorization variable(s) used in your manuscript and explain why they were used. Please note that such variables should not be used as proxies for other socially constructed/relevant variables (for example, race or ethnicity should not be used as a proxy for socioeconomic status).*<br>*Provide clear definitions of the relevant terms used, how they were provided (by the participants/respondents, the researchers, or third parties), and the method(s) used to classify people into the different categories (e.g. self-report, census or administrative data, social media data, etc.)*<br>*Please provide details about how you controlled for confounding variables in your analyses.*
Population characteristics	Patient demographic data are highlighted in Supplementary Figure 12 figure legend.<br>Liver sections from patients with a history of liver transplantation and repeated events of liver ischemia, with history of complex anatomical heart defect and life-long chronic liver congestion, and with a history of hepatic hemangiomatosis were analysed.
Recruitment	Tissue Biobank
Ethics oversight	Patient samples were obtained under licence 2022-140_1_S_KH approved by the local ethics committee of TUM/MRI under the legal guidelines of the Government of Upper Bavaria (BayKrG Art. 27 Abs. 4), with a waiver of informed consent due to retrospective and anonymised secondary use of clinical specimens.

Note that full information on the approval of the study protocol must also be provided in the manuscript.

Field-specific reporting

Please select the one below that is the best fit for your research. If you are not sure, read the appropriate sections before making your selection.

☒ Life sciences ☐ Behavioural & social sciences ☐ Ecological, evolutionary & environmental sciences

For a reference copy of the document with all sections, see nature.com/documents/nr-reporting-summary-flat.pdf

Life sciences study design

All studies must disclose on these points even when the disclosure is negative.

Sample size	No statistical method was used to estimate sample size. Experiments were generally conducted with at least n = 4 biological samples.
Data exclusions	For in vivo experiments, animals were excluded that displayed abnormal behaviour or reached defined termination criteria after interventions. For scRNA-seq analysis, cells were filtered based on the number of detected genes (hepatocytes > 1,000 genes and< 7,000 genes; LSEC > S00 genes and< 4,000 Genes). Contaminating cell types, such as immune cells or vascular mural cells, were manually removed based on congruent marker expression.<br>For FACS analysis, cells were gated based on size (exclusion of cell debris and cell doublets) and viability (FxCycle Violet). Hepatocytes were sorted based on their size (forward and side scatter) and LSEC based on CD31 and CD146 expression. CD45+ immunes cells were excluded.<br>For gene expression analysis, samples with poor mRNA quality (RIN<7 for sequencing, Ct of Actb > 25 for qPCR) were excluded.

Replication	To ensure the replication of the findings, data were replicated over different breeding pairs of animals.<br>Biological replicates are clearly indicated for each figure panel and in the respective figure legends.
Randomization	For in vitro experiments, cells were randomly assigned to either control or treatment group. For in vivo studies, mice were randomly assigned to either control or treatment group.
Blinding	Sample processing after euthanasia was performed in a blinded manner. Notably, for metabolomic screening, proteomic screening, and sequencing, data processing, and the downstream analyses were performed blinded.

Reporting for specific materials, systems and methods

We require information from authors about some types of materials, experimental systems and methods used in many studies. Here, indicate whether each material, system or method listed is relevant to your study. If you are not sure if a list item applies to your research, read the appropriate section before selecting a response.

Materials & experimental systems

n/a	Involved in the study	
☐	☒	Antibodies
☐	☒	Eukaryotic cell lines
☒	☐	Palaeontology and archaeology
☐	☒	Animals and other organisms
☒	☐	Clinical data
☒	☐	Dual use research of concern
☒	☐	Plants

Methods

n/a	Involved in the study	
☒	☐	ChIP-seq
☐	☒	Flow cytometry
☒	☐	MRI-based neuroimaging

Antibodies

Antibodies used	For immunostaining, the following primary antibodies were used: rabbit anti-mouse Glutamine Synthetase (1:1,000, Abeam, ab49873), goat anti-mouse Collagen IV (1:200, Novus, NBPI-26549); rabbit anti-mouse APC (1:100, Novus, NBI00-91662), goat anti-mouse FZD4 (1:100, Novus, AF1A94), goat anti-mouse LRP6 (1:100, Novus, NBI00-1475), rat anti-mouse KI67 (1:200, Invitrogen, 14-5698-37), rabbit anti-mouse HNF4A (1:200, Abeam, ab92378), rabbit anti-mouse Cytochrome P4S0 2E1 (CYP2E1, 1:500, Abeam, ab28146), goat anti-mouse VE-Cadherin (1:200, R&D, AF1002), goat anti-mouse ICAM1 (1:200, R&D, AF796), mouse anti-mouse eNOS (1:200, Cell Signaling, 5880), rabbit anti-mouse GJA4 (1:1,000, Invitrogen, 40-4300), rat anti-PECAM1 (clone 1G5 and SD2, provided by Kiefer lab), rabbit anti-Cytokeratin 19 (14965-1-AP, proteintech), and Phalloidin (1:200, Invitrogen, A22283).<br>For Proximity ligation assay, the following primary antibodies were used: rat ant-mouse ITGB1 (1:100, BD Bioscience, SS3715), goat anti-mouse VEGFR3 (1:100, R&D, AF743), rabbit anti-mouse Phosphoserine (1:100, Abnova, PAB10351), and rabbit anti-mouse Phosphotyrosine (1:100, Abnova, MAB12826). The counterstaining was performed using: rabbit anti-mouse Glutamine Synthetase (1:1,000, Abeam, ab49873), goat anti-mouse VE-Cadherin (1:200, R&D, AF1002), and rat ant-mouse ITGB1 (1:100, BD Bioscience, 553715).<br>For in situ hybridization assay, Gja1 probe (VB6-17027-VT, Thermo Fisher Scientific) were hybridized followed by goat anti-mouse VE-Cadherin (1:200, R&D, AF1002) and rabbit anti-mouse Glutamine Synthetase staining (1:1,000, Abeam, ab49873).<br>For FACS, the following primary antibodies were used: CD73-FITC (1:200, Biolegend, 127219), CD324-PE (1:200, Biolegend, 147304), CD45-FITC (1:200. BD Bioscience, SS3775), CD31-APC (1:200, BD Bioscience, SS1262), CD31-PE (1:200, BD Bioscience, 553373), CD146-PE-Cy7 (1:200, Biolegend, 134713), and CD117-APC (1:200, Biolegend, 10S812).<br>For immunoblotting, the following primary antibodies were used: rabbit anti-mouse GJA1 (1:2,000, Abeam, ab11370), rabbit anti-mouse GJA4 (1:1,000, Invitrogen, 40-4300), rat ant-mouse ITGB1(CD29) (1:100, BD Bioscience, 5S3715), goat anti-mouse VEGFR3 (1:1,000, R&D, AF743), rabbit anti-mouse Phospho-VEGFR3 Tyr1230 (1:1,000, Affinity Biosciences, AF3676), and rabbit anti-mouse Tubulin (1:1,000, Cell Signaling, 2148S). Consequently, the following secondary antibodies were used: goat anti-rabbit HRP (1:5,000, DAKO, P016002-2) and rabbit anti-goat HRP (1:5,000, DAKO, P044801-2). All antibodies were validated for the indicated species and applications by the manufacturer.
Validation	All antibodies were validated for the indicated species and applications by the manufacturer.

Eukaryotic cell lines

Policy information about cell lines and Sex and Gender in Research

Cell line source(s)	Primary LSEC were directly and freshly isolated from mice for each experiment.<br>HEK293T TCF4/Wnt-mCherry Cas9-GFP line was generated in the laboratory of Michael Boutros.
Authentication	Primary LSEC were isolated using CD146 microbeads. Cell type were authenticate by morphology, i.e. visualising gaps and fenestrations. Furthermore, LSEC identity and purity was verified by gene expression analysis of cell marker genes.
Mycoplasma contamination	Cell lines were routinely tested for mycoplasma contamination.

Commonly misidentified lines
(See ICLAC register)

No commonly misidentified cell lines were used in the study.

Animals and other research organisms

Policy information about studies involving animals; ARRIVE guidelines recommended for reporting animal research, and Sex and Gender in Research

Laboratory animals

Wildtype C57BL/6J, B6 Evi/Wls fl/fl x Cdh5-CreERT2, B6 Rspo3 fl/fl x Cdh5-CreERT2, and the double mutant line Evi/Wls fl/fl x Rspo3 fl/fl x Cdh5-CreERT2 were used for the study. Cre-positive mice used in this study were homozygous for the floxed alleles and heterozygous for Cre. Cre-negative littermates that were homozygous for the floxed alleles served as controls. All animals were housed and maintained in pathogen-free animal facilities of the German Cancer Research Centre. In brief, all mice were housed on a temperature controlled 12-hour light/12-hour dark cycle and had free access to water and food ad libitum.

Wild animals

No wild animals are used in the study.

Reporting on sex

Mixed sex of wildtype C57BL/6J, B6 Evi/Wls fl/fl x Cdh5-CreERT2, B6 Rspo3 fl/fl x Cdh5-CreERT2, and the double mutant line Evi/ sfl/fl x Rspo3fl/fl x Cdh5-CreERT2 were used for the study. Sex of mice are specified on each of the figure legends.

Field-collected samples

No field-collected samples are used in the study.

Ethics oversight

All in vivo studies were performed in accordance with German national guideline on animal welfare and the regulations of the gional council Karlsruhe. The individual permit numbers are listed in the methods section.

Note that full information on the approval of the study protocol must also be provided in the manuscript.

Plants

Seed stocks

Report on the source of all seed stocks or other plant material used. If applicable, state the seed stock centre and catalogue number. If plant specimens were collected from the field, describe the collection location, date and sampling procedures.

Novel plant genotypes

Describe the methods by which all novel plant genotypes were produced. This includes those generated by transgenic approaches, gene editing, chemical/radiation-based mutagenesis and hybridization. For transgenic lines, describe the transformation method, the number of independent lines analyzed and the generation upon which experiments were performed. For gene-edited lines, describe the editor used, the endogenous sequence targeted for editing, the targeting guide RNA sequence (if applicable) and how the editor was applied.

Authentication

Describe any authentication procedures for each seed stock used or novel genotype generated. Describe any experiments used to assess the effect of a mutation and, where applicable, how potential secondary effects (e.g. second site T-DNA insertions, mosiacism, off-target gene editing) were examined.

Flow Cytometry

Plots

Confirm that:

☒ The axis labels state the marker and fluorochrome used (e.g. CD4-FITC).

☒ The axis scales are clearly visible. Include numbers along axes only for bottom left plot of group (a 'group' is an analysis of identical markers).

☒ All plots are contour plots with outliers or pseudocolor plots.

☐ A numerical value for number of cells or percentage (with statistics) is provided.

Methodology

Sample preparation

Mice were sacrificed by cervical dislocation and livers were exposed for in situ perfusion. For this, the vena cava was fixed using a 27G winged infusion set (B.Braun) connected to the tubing of a peristaltic pump (Ismatec). The liver was perfused with 37°C pre-warmed liver perfusion medium (Gibco) at 4ml/min for 2min, followed by 37°C pre-warmed liver digestion medium (Gibco) supplemented with 20µg/ml Liberase TM (Roche) at 3ml/min for Smin. Perfused livers were explanted and located into a Petri dish with a pre-warmed RPMI medium (Gibco). After removing the liver capsule membrane, the tissue was dissociated by gentle shaking in a final volume of 40ml of RPMI.
For hepatocyte collection, the single cell suspension was filtered through a 100µ m cell strainer (BD Bioscience) and centrifuged once at 50g and 4°C for Smin. The resulting cell pellet contained hepatocytes were resuspended in FACS buffer and sorted using BD FACS Aria I and II (BD Bioscience).
For non-parenchymal hepatic cells (NPC) isolation, the supernatant was collected and centrifuged for another Smin at 50g and 4°C to remove excessive parenchymal cells. The supernatant was collected, and the final NPC population was obtained by centrifugation at 300g and 4°C for IOmin. Red blood cells were lysed by incubating the isolated cells in ACK buffer for Smin. To purify LSEC, total NPC were resuspended in 200µl of FACS buffer containing 5% FCS and incubated with 20µl of mouse CD146 MicroBeads (Miltenyi Biotec) for 20min on ice. The cells were washed once, resuspended in 1ml of FACS buffer and loaded onto a LS column (Miltenyi Biotec) and cells were purified according to the manufacturer's instruction. The

solated LSEC were then processed for flow cytometry.

Instrument

BD FACS Aria cell sorter (BD Bioscience) and BD FACS Fusion cell sorter (BD Bioscience)

Software

BD FACSDiva 8.0 (BD Bioscience), FlowJo VI0 (BD Bioscience)

Cell population abundance

The abundance of the relevant cell populations within post-sort fractions was 90-98% in experiment

Gating strategy

Hepatocytes were gated based on (1) forward scatter area (FSC-A) and side scatter area (SSC-A) to exclude NPC and cell debris, (2) forward scatter width (FSC-W) and forward scatter height (FSC-H), as well as side scatter width (SSC-W) and side scatter height (SSC-H) to exclude cell doublets, and (3) FxCycle Violet staining at a dilution of 1:1,000 (Invitrogen) to exclude dead or damaged cells. For spatial sorting, hepatocytes were stained with CD73-FITC (Biolegend) and CD324-PE (Biolegend). Central vein hepatocytes were identified as CD73+ and CD324-, whereas portal vein hepatocytes were considered CD73- and CD324+. Pericentral and periportal hepatocytes were characterized by intermediate expression of CD73 and CD324, respectively.
For FACS sorting of LSEC, LSEC were isolated based on (1) FSC-A and SSC-A to exclude cell debris and cell clumps, (2) FSC-A and FSC-H to exclude cell doublets, (3) FxCycle Violet stain at a dilution of
1:1,000 to exclude dead or damaged cells, and (4) CD45 negative, and CD31 and CD146 double positive
staining to exclude contaminating cell types. For the spatial sorting of LSEC, cells were stained with CD117 (c-Kit)-APC (Biolegend). LSEC were sorted into four equal portions along the c-Kit expression gradient, with CD117high representing LSEC round the central vein and CD117low representing LSEC from the portal vein.

☒ Tick this box to confirm that a figure exemplifying the gating strategy is provided in the Supplementary Information.
