# Supplementary material for: Haemodynamic control of zonated liver function via instructive vascular Wnt signalling
Source: Nat Commun. 2026 Aug 22;17:8821. doi: 10.1038/s41467-026-76966-7 (PMC13499805; doi:10.1038/s41467-026-76966-7)
Supplement: Supplementary file 4 [file 41467_2026_76966_MOESM8_ESM.pdf]

nature portfolio

Peer Review File

Haemodynamic control of zonated liver function via instructive vascular Wnt signalling

Corresponding Author: Professor Hellmut Augustin

This manuscript has been previously reviewed at another journal. This document only contains information relating to versions considered at Nature Communications.

This file contains all reviewer reports in order by version, followed by all author rebuttals in order by version.

Version 0:

Reviewer comments:

Reviewer #4

(Remarks to the Author)
I commend the authors for investigating and identifying a mechanism by which biophysical forces regulate Wnt angiocrine signalling to instruct liver zonation. I have reviewed the revised manuscript in light of Reviewer #3's comments, and in my opinion the additional data, clarifications and revised wording have satisfactorily addressed the major concerns.
The only issue that, in my view, still warrants attention is the limited sample size in some experiments. Although most statistical software can calculate a p value from as few as three data points, this does not represent good scientific practice. I therefore recommend that all experiments be supported by a minimum of four independent biological replicates, with six being preferable where feasible.

(Remarks on code availability)

Open Access This Peer Review File is licensed under a Creative Commons Attribution 4.0 International License, which permits use, sharing, adaptation, distribution and reproduction in any medium or format, as long as you give appropriate credit to the original author(s) and the source, provide a link to the Creative Commons license, and indicate if changes were made.
In cases where reviewers are anonymous, credit should be given to 'Anonymous Referee' and the source.
The images or other third party material in this Peer Review File are included in the article's Creative Commons license, unless indicated otherwise in a credit line to the material. If material is not included in the article's Creative Commons license and your intended use is not permitted by statutory regulation or exceeds the permitted use, you will need to obtain permission directly from the copyright holder.
To view a copy of this license, visit https://creativecommons.org/licenses/by/4.0/

Reviewer #4

GENERAL COMMENT 1: I commend the authors for investigating and identifying a mechanism by which biophysical forces regulate Wnt angiocrine signalling to instruct liver zonation. I have reviewed the revised manuscript in light of Reviewer #3's comments, and in my opinion the additional data, clarifications and revised wording have satisfactorily addressed the major concerns.

RESPONSE TO GENERAL COMMENT 1: We sincerely thank the Reviewer for positively evaluating our manuscript. A concerted effort was made to address experimentally and editorially all remaining comments of this Reviewer.

COMMENT 1: The only issue that, in my view, still warrants attention is the limited sample size in some experiments. Although most statistical software can calculate a p value from as few as three data points, this does not represent good scientific practice. I therefore recommend that all experiments be supported by a minimum of four independent biological replicates, with six being preferable where feasible.

RESPONSE TO COMMENT 1: We sincerely appreciate the Reviewer's critical assessment. To address this issue, we have thoroughly reviewed our manuscript and identified the experiments that require larger sample sizes. Consequently, the below spelled out experiments, including assays showing the consequences of oscillating shear, laminar shear, hypoxia, $CoCl_2$, WNT5A, LGK974, and CHIR99021 on primary LSEC were performed. Furthermore, additional immunoblotting, immunofluorescence, and proximal ligation assays were performed to strengthen the scientific rigor of the manuscript.

To precisely dissect the distinct shear responses of primary LSEC, we have repeated key experiments which demonstrate the biophysical regulation of vascular Wnt expression. We have successfully validated that vascular *Wnt2* expression was regulated by both oscillatory and laminar shear stress (**Rebuttal Figure 1 and 2**). Consequently, we have additionally performed gene expression analyses of oscillating shear markers, including *Icam1* and *Vcam1*, to corroborate the validity of our IVF dish-guided shear stress model. As expected, *Icam1* and *Vcam1* were solely induced by the oscillating shear (**Rebuttal Figure 1**). These results not only validated that hemodynamic stimuli induce vascular Wnt expression but also confirmed the validity of the IVF dish-based assay, which clearly distinguished the contributions of oscillating and laminar shear stress. Therefore, these results have been incorporated in the further revised manuscript (**now Extended Data Figure 1e-p**), replacing the previous figure (**Extended Data Figure 1e-l**). The corresponding texts can be found in Lines 76-79 and Line 91-93.

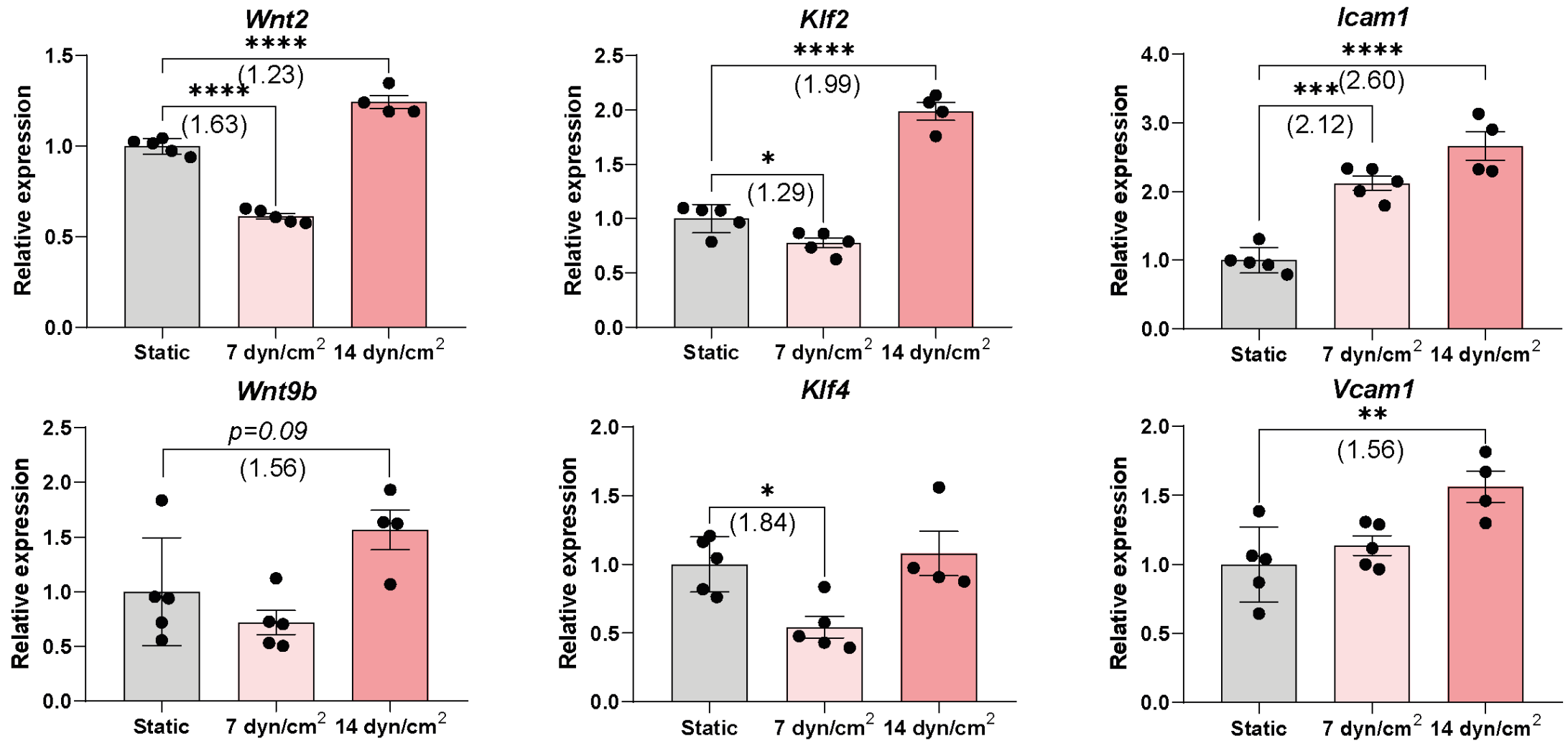

Rebuttal Figure 1: Transcriptional dynamics of shear-sensitive and vascular Wnt genes under different oscillatory shear conditions. 500,000 LSEC were seeded on the central area of the IVF dish and oscillating shear stress was applied using an orbital shaker (0, 7, 14 dyn/cm^2) for 12 hours. Relative gene expression of *Wnt2*, *Wnt9b*, *Klf2*, *Klf4*, *Icam1*, and *Vcam1* in LSECs under different oscillatory shear conditions (Static; n=5, 7 dyn/cm^2; n=5, 14 dyn/cm^2; n=4) were analysed using quantitative PCR. Gene expression was normalised to Pecam1. () Fold change was calculated relative to the static samples. Data are presented as mean±s.d. p value by one-way ANOVA is shown. *P < 0.05, **P < 0.01, ***P < 0.001, **** P < 0.0001.

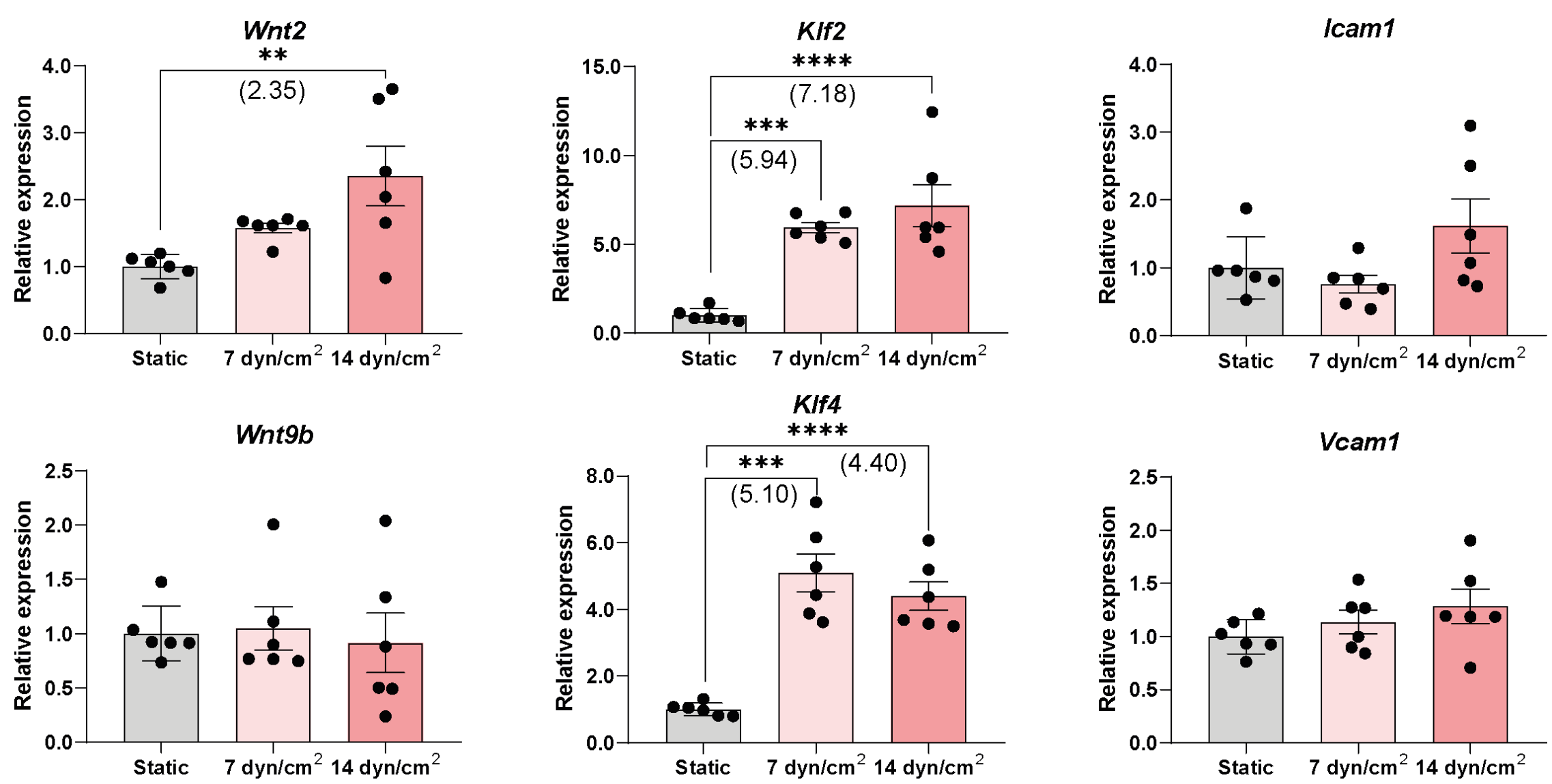

Rebuttal Figure 2: Transcriptional dynamics of shear-sensitive and vascular Wnt genes under different laminar shear conditions. Two million LSEC were seeded on the peripheral area of the IVF dish and laminar shear stress was applied using an orbital shaker (0, 7, 14 dyn/cm^2) for 12 hours. Relative gene expression of Wnt2, Wnt9b, Klf2, Klf4, Icam1, and Vcam1 in LSECs under different oscillatory shear conditions (Static; n=6, 7 dyn/cm^2; n=6, 14 dyn/cm^2; n=6) was assessed via quantitative PCR. Gene expression was normalised to Pecam1. () Fold change was calculated relative to the static samples. Data are presented as mean±s.e.m. p value by one-way ANOVA is shown. **P < 0.01, ***P < 0.001, **** P < 0.0001.

Due to limited resources, we were unable to repeat the experiment using a pharmacological approach *in vitro* with a larger cohort. Thus, we decided to exclude the results involving Yoda1 (**Extended Data Figure 1q-u; Line 88-92; Line 568-569; Line 1165-1168**). However, this does not weaken the quality of the manuscript, as we pharmacologically demonstrate the concept of hemodynamic regulation of vascular Wnt expression *in vivo* using the vasodilating agent Riociguat (**Extended Figure 2g-k**). Yet, we noted that a small sample size was considered when validating the vasodilatory functions. To address this issue, we further demonstrated the validity of the vasodilator using a larger cohort. In line with the previous result, upregulation of activated ITGB1 and VEGFR3 phosphorylation was observed (**Rebuttal Figure 3**). Furthermore, similar to the previous result, we did not observe a significant difference in total KLF2 expression. We assume that total KLF2 expression does not reflect its function, as it does not represent nuclear KLF2 levels. Therefore, the statement involving KLF2 at line 108 is now excluded. These results are incorporated in the revised manuscript, replacing the previous figure (**Extended Data Figure 2n-q**).

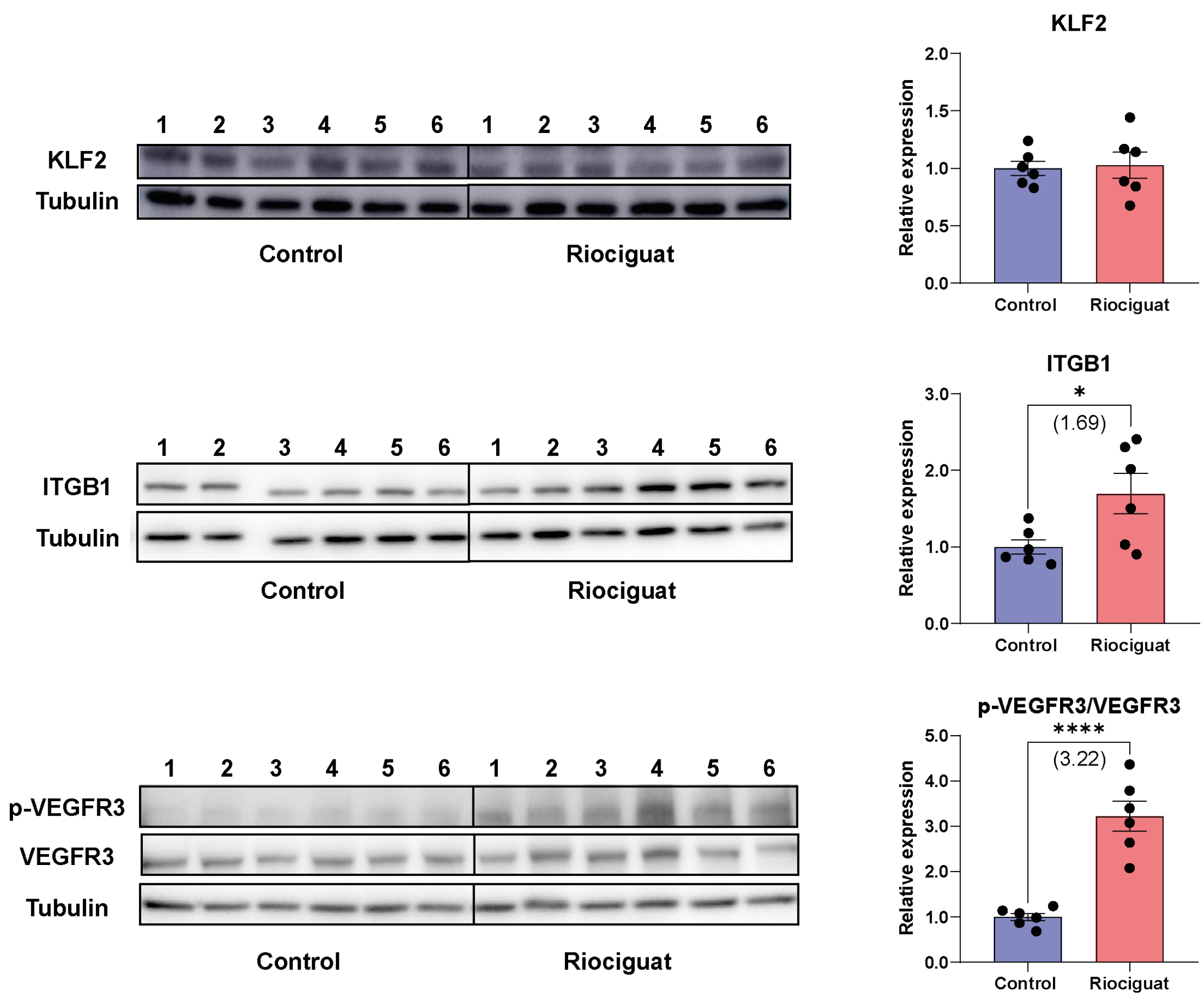

Rebuttal Figure 3: Immunoblotting of shear-sensitive proteins. KLF2, ITGB1, p-VEGFR3, VEGFR3, and corresponding Tubulin expression were analyzed from mouse liver lysates treated with either a vehicle control (n=6) or the vasodilating agent Riociguat (n=6). () Fold change was calculated relative to the vehicle control. Data are presented as mean±s.e.m. p value by two-tailed t-test is shown. *P < 0.05, ****P < 0.0001.

One of our major findings indicates that hypoxia is not the major milieu factor regulating vascular Wnt expression. Yet, we noted that some *in vitro* and immunostaining experiments were previously performed with a limited sample size. To address this concern, we repeated those key experiments. First, primary LSEC were cultured under either hypoxic (1%) or normoxic (21%) conditions. Upregulation of *Slc2a1* was observed, reflecting the consequences of hypoxia in primary LSEC. In line with the previous findings, we confirmed that vascular Wnt expression is not induced by hypoxia (**Rebuttal Figure 4**). To further validate this result, we repeated the *in vitro* assays involving the hypoxia-inducible factor (HIF) stabilizing agent, cobalt chloride ($CoCl_2$). As expected, *Slc2a1* expression was induced upon $CoCl_2$ application. Consistent with the result, *Wnt2* expression was repressed under $CoCl_2$ treatment (**Rebuttal Figure 5**). In parallel, 400 µM of $CoCl_2$ treatment resulted in a significant loss of cells and was therefore excluded from the analysis. Next, we performed hypoxyprobe staining to further strengthen the *in vivo* data. Consequently, we confirmed an increase in the hypoxyprobe-labelled region after the HIF prolyl-hydroxylase treatment (**Rebuttal Figure 6**). Overall, these data further support that hypoxia is dispensable for vascular Wnt induction. These results are incorporated in the revised manuscript (**Extended Data Figure 3a-f,r,s**), replacing the previous figures (**Extended Data Figure 3a-d,m,n**). The corresponding text can be found in Line 124-125 and 128-130.

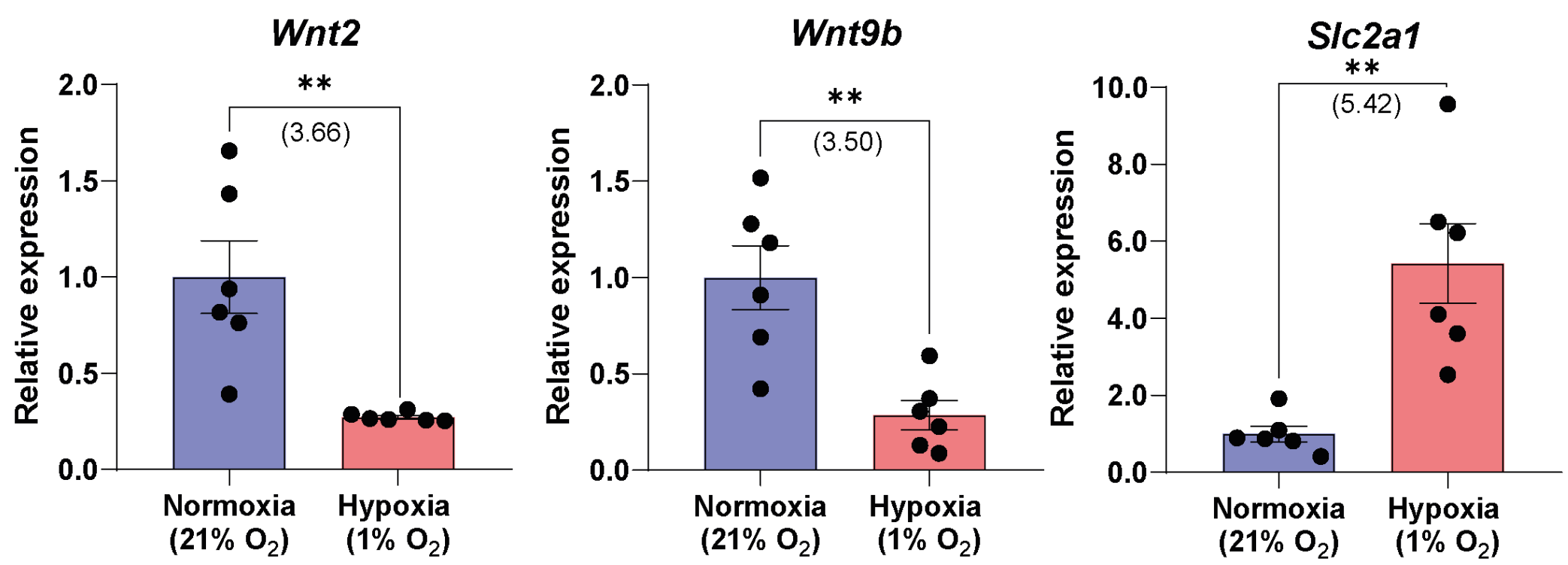

Rebuttal Figure 4: Vascular Wnt expressions under normoxic and hypoxic conditions. Relative gene expression of *Wnt2*, *Wnt9b*, and *Slc2a1* in primary LSEC cultured under either atmospheric oxygen (21%, n=6) or hypoxic (1%, n=6) conditions for 24 hours was evaluated by quantitative PCR. Fold change was calculated relative to the normoxic condition. Data are presented as mean±s.e.m. p value by two-tailed t-test is shown. **$P < 0.01$,

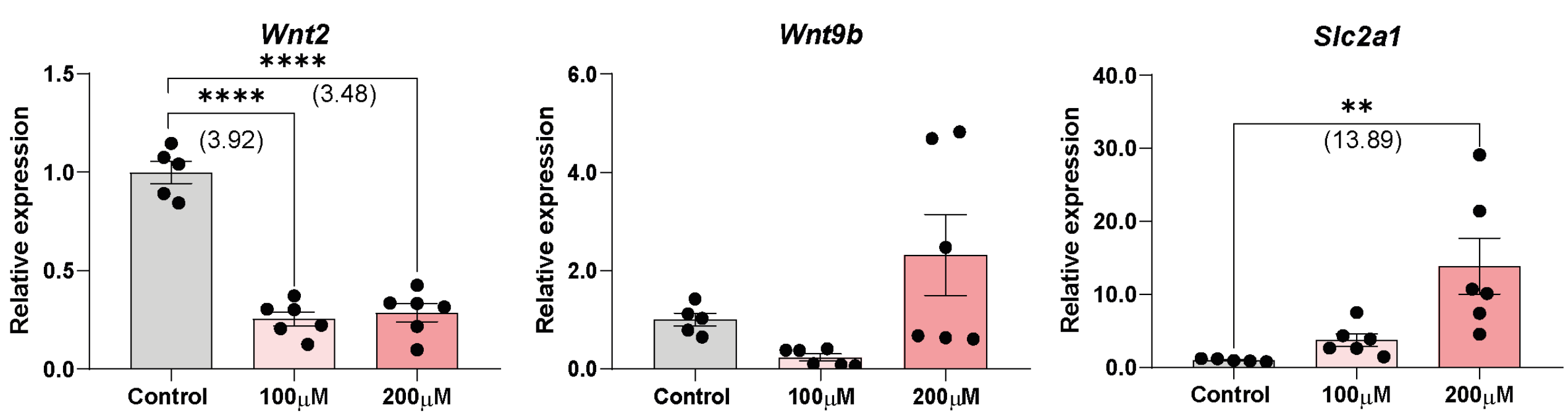

Rebuttal Figure 5: Vascular Wnt expressions upon cobalt chloride treatment. Relative gene expression of *Wnt2*, *Wnt9b*, and *Slc2a1* in primary LSEC cultured under different concentrations of $CoCl_2$ was evaluated. The cells were cultured either in vehicle control (n=5), 100µM (n=6), or 200µM of $CoCl_2$ (n=6) for 24 hours. Gene expression was assessed by quantitative PCR. Fold change was calculated relative to the vehicle control. Data are presented as mean±s.e.m. p value by one-way ANOVA is shown. **$P < 0.01$, ****$P < 0.0001$

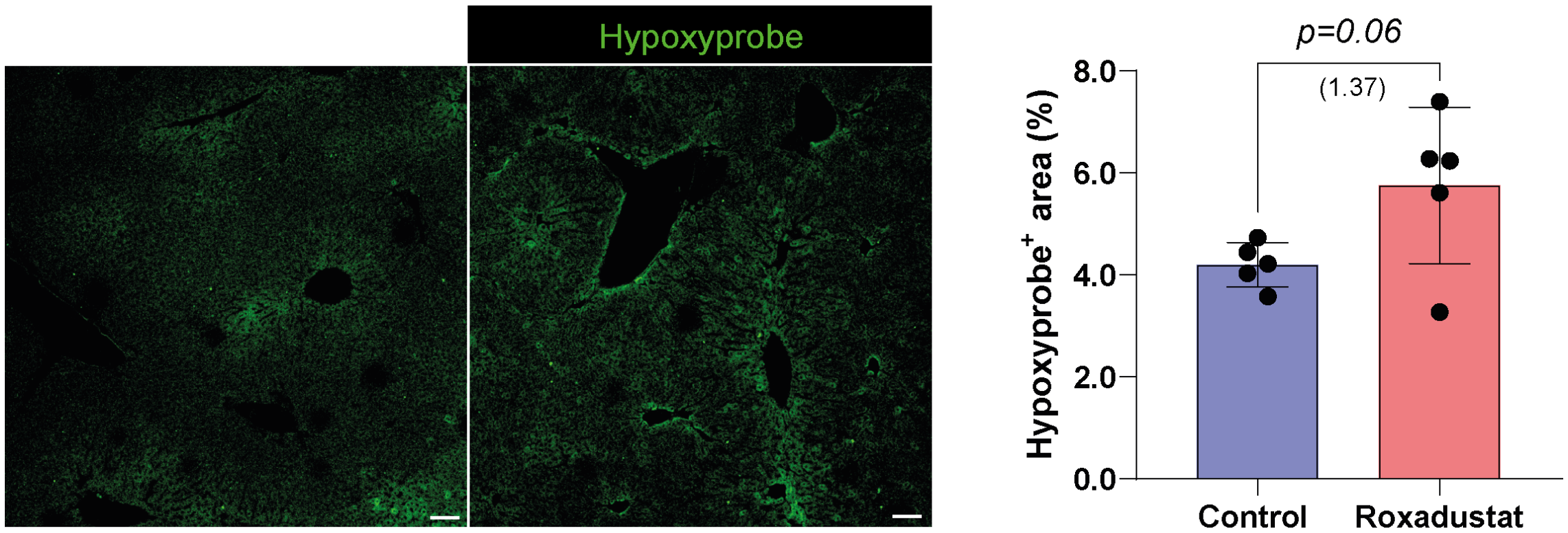

Rebuttal Figure 6: Hypoxyprobe labelling of liver after HIF prolyl-hydroxylase inhibitor administration. Liver sections from either vehicle control (n=5) or HIF prolyl-hydroxylase, Roxadustat, treated (n=5) mouse were subjected to hypoxyprobe labeling. Hypoxic area is marked by hypoxyprobe (green). Scale bar shows 100µm. Fold change was calculated relative to the vehicle control. Data are presented as mean±s.d., and the p-value by a two-tailed t-test is shown.

Notably, we have previously demonstrated the autocrine functions of vascular Wnt factors in regulating gap junction molecules. Yet, we realized that some of the experiments were performed with a limited sample size. To address this issue, we repeated some of the key experiments. We first dissected the transcriptional dynamics following non-canonical WNT5A treatment on primary LSEC *in vitro*. Intriguingly, 200ng/ml of recombinant WNT5A treatment induced both *Gja1* and *Gja4* expression, highlighting that the non-canonical Wnt signaling pathway is crucial for gap junctional molecules (**Rebuttal Figure 7**). The following figure is now incorporated in he manuscript in **Figure 5h and Extended Data Fig. 11e**, respectively. For orthogonal validation, we performed immunoblotting assays to assess the observed phenotype at the protein level. We have previously shown that recombinant WNT2 and WNT9B induced gap junctional molecule expression (Figure 5i-l). Yet, due to limited resources, we could not repeat the experiment with a larger cohort. To ameliorate this issue, we now performed immunoblotting assays on primary LSEC treated with either a Wnt agonist, CHIR99021, or an antagonist, LGK974. In line with the previous data (**Figure 5f,**g), GJA4 expression was precisely regulated by Wnt signaling (**Rebuttal Figure 8**). Interestingly, the data further show that GJA1 expression is induced by Wnt agonist treatment, mimicking the functions of recombinant WNT2 and WNT9B (**Rebuttal Figure 8**). The data are now incorporated in the revised manuscript (**Extended Data Fig. 11f-k**) with the following text (Line 359-362). For *in vivo* validation, we previously performed immunoblotting and immunostaining of gap junctional molecules. We planned to repeat both experiments. Yet, due to limited resources, we could not repeat the immunoblotting assays on the mutant samples (**Extended Data Figure 10m-o**). We, therefore, decided to replace the figure with **Rebuttal Figure 8** as shown above. In parallel, we have performed immunostaining of GJA4 with a larger cohort to validate the original findings. Consequently, we confirmed the loss of GJA4 expression in the central vein region of the mutant. In contrast, GJA4 expression was retained in the portal vein region, indicating that GJA4 expression is regulated locally by the autocrine Wnt code (**Rebuttal Figure 9**). The following results replace **Figure 5c,d and Extended Data Figure 10k,l**. Overall, these data further highlight that both non-canonical and canonical Wnt signaling are indispensable for gap junctional molecules.

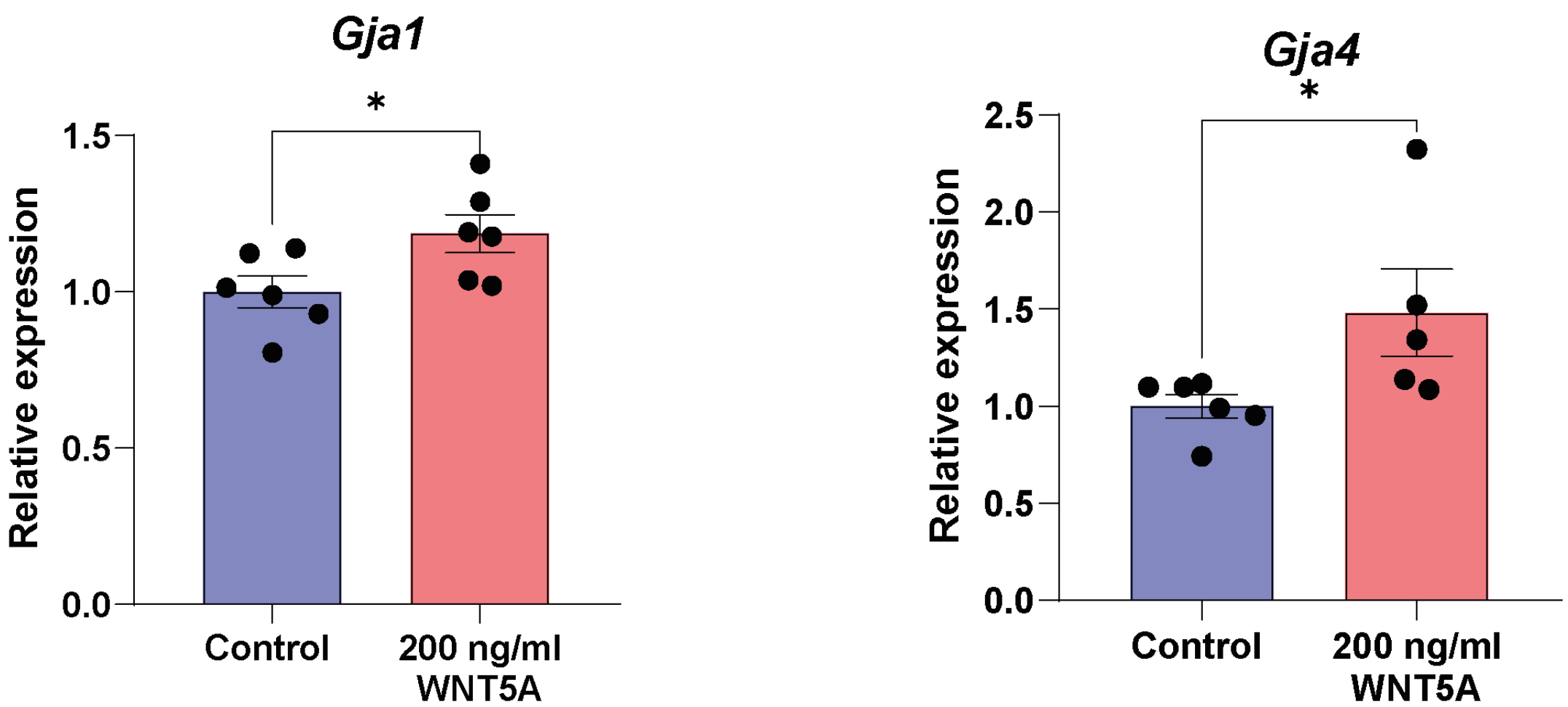

Rebuttal Figure 7: Gene expression of gap junctional molecules after non-canonical WNT5A treatment. Relative gene expression of *Gja1* and *Gja4* in primary LSEC cultured under either control or 200 ng/ml of recombinant WNT5A proteins was evaluated. The cells were cultured either in control (n=6) or 200 ng/ml (n=6) of WNT5A for 24 hours. Gene expression was assessed using quantitative PCR. Fold change was calculated relative to the control treatment. Data are presented as mean±s.e.m and the p-value by a two-tailed t-test is shown. *P < 0.05

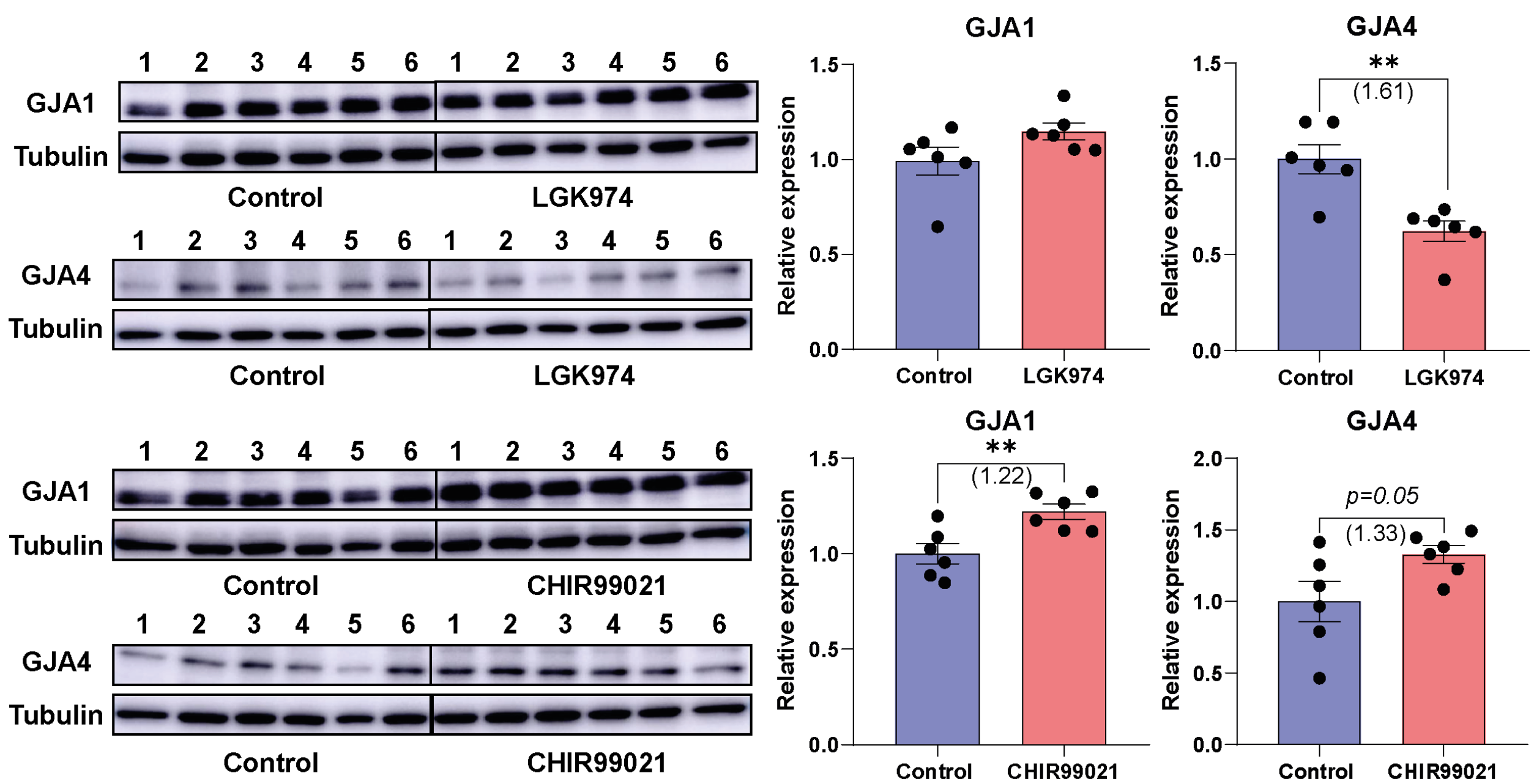

Rebuttal Figure 8: Immunoblotting of gap junctional molecules after Wnt inhibition and Wnt activation. Relative protein expression of GJA1 and GJA4 in primary LSEC cultured either under Wnt-inhibiting, LGK974, or Wnt-activating, CHIR99021, or their vehicle control was evaluated. The cells were cultured either in vehicle control for LGK974 (n=6), LGK974 (n=6), vehicle control for CHIR99021 (n=6) or CHIR99021 (n=6) for 24 hours. GJA1, GJA4, and their corresponding Tubulin expression were analysed by immunoblotting. Fold change was calculated relative to each control treatment. Data are presented as mean±s.e.m and the p-value by a two-tailed t-test is shown. **P < 0.01

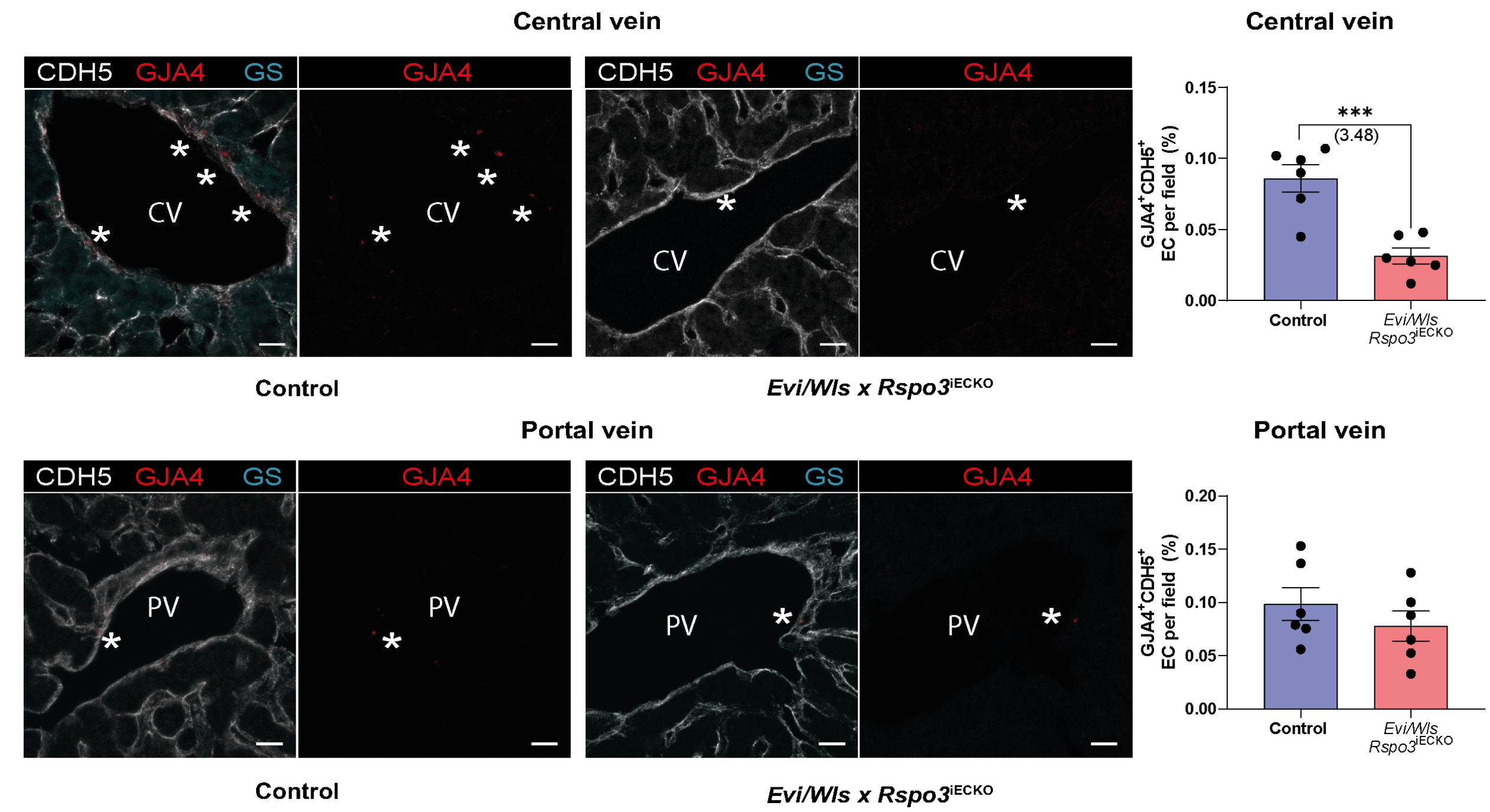

Rebuttal Figure 9: Immunofluorescence imaging of GJA4 from the liver of Wnt-deficient mice and their littermate control. Representative image of GJA4 from the portal vein and central vein region. Liver sections from the mutant mice (n=6) and littermate control (n=6) were stained with GS (cyan) to mark the central venous area, CDH5 (white) to indicate the vessel, and GJA4 (red), respectively. The portal vein and central vein are indicated as PV and CV. GJA4+ endothelial cells are highlighted by an asterisk. Scale bar indicates 10 µm. Quantification of the GJA4+CDH5+ area is presented. Fold change was calculated relative to the littermate control. Data are presented as mean±s.e.m and p value by two-tailed t-test is shown. ***P < 0.001

To further improve the quality of the manuscript, we have strengthened the findings of zonated shear response in the hepatic vasculature. By performing additional proximal ligation assays with ITGB1 and phospho-tyrosine antibody, zonal bias of ITGB1 tyrosine-phosphorylation towards the central vein was observed (**Rebuttal Figure 10**). This finding is now incorporated in Extended Data Figure 13d.

In summary, we have successfully performed validation experiments with a larger cohort to confirm our key findings. The omics data presented here were analyzed using three to four independent samples per group, and we believe this represents the state of the art, as many omics studies are still being processed, even with only one or two samples. Furthermore, we took this opportunity to expand the discussion by incorporating the most recent publications in the field. The updated discussions can be found in Line 396-399 and Line 439-442. Lastly, we would like to thank the reviewer for the comment and for the opportunity to further improve the manuscript.

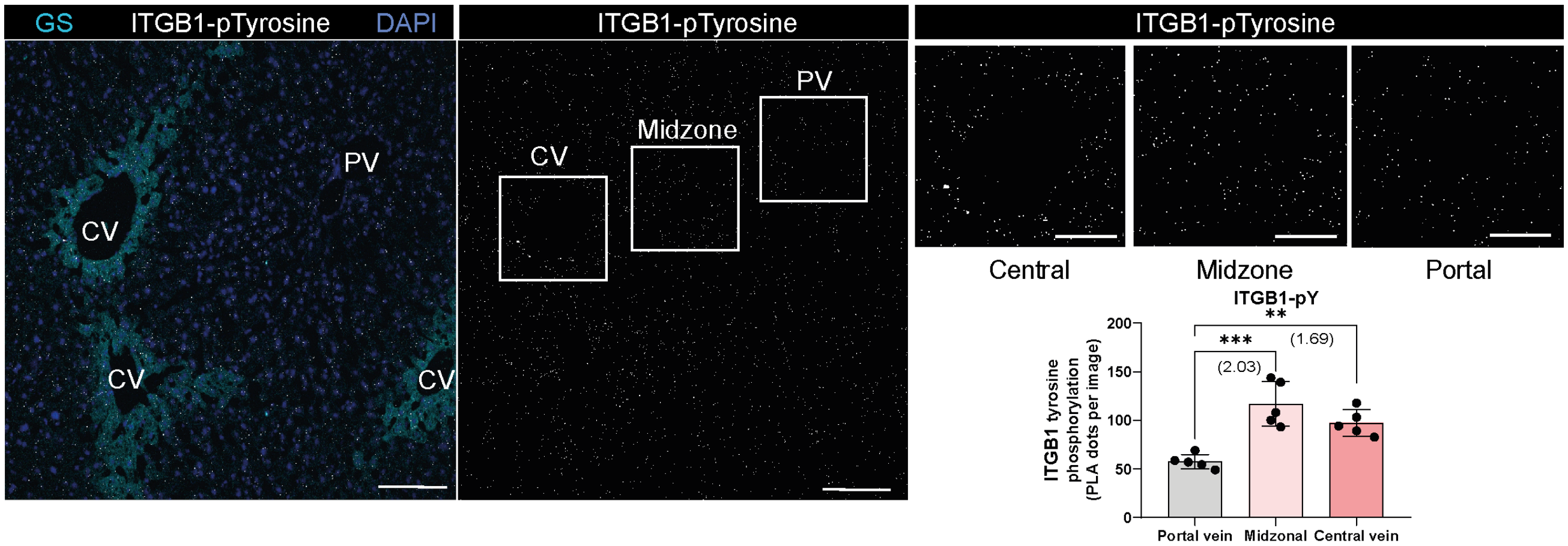

Rebuttal Figure 10: Proximal ligation assay of ITGB1 and phospho-tyrosine in the liver. Liver sections from the wildtype mice (n=5) were stained with GS (cyan) to mark the central venous area, DAPI (blue) to indicate the nucleus, and ITGB1-pTyrosine (white), respectively. Proximal ligation assay of ITGB1-pTyrosine was performed according to the manufacturer's instructions. The portal vein, midzonal, and central vein area are indicated as PV, Midzone, and CV. Scale bar indicates 100 µm and 10 µm for the zoomed region. Quantification of the ITGB1-tyrosine phosphorylation (PLA dots per region) is presented. Fold change was calculated relative to the portal vein. Data are presented as mean±s.d. and p value by two-tailed t-test is shown. **P<0.01, ***P < 0.001
