# Supplementary material for: Haemodynamic control of zonated liver function via instructive vascular Wnt signalling
Source: Nat Commun. 2026 Aug 22;17:8821. doi: 10.1038/s41467-026-76966-7 (PMC13499805; doi:10.1038/s41467-026-76966-7)

Figure 5i

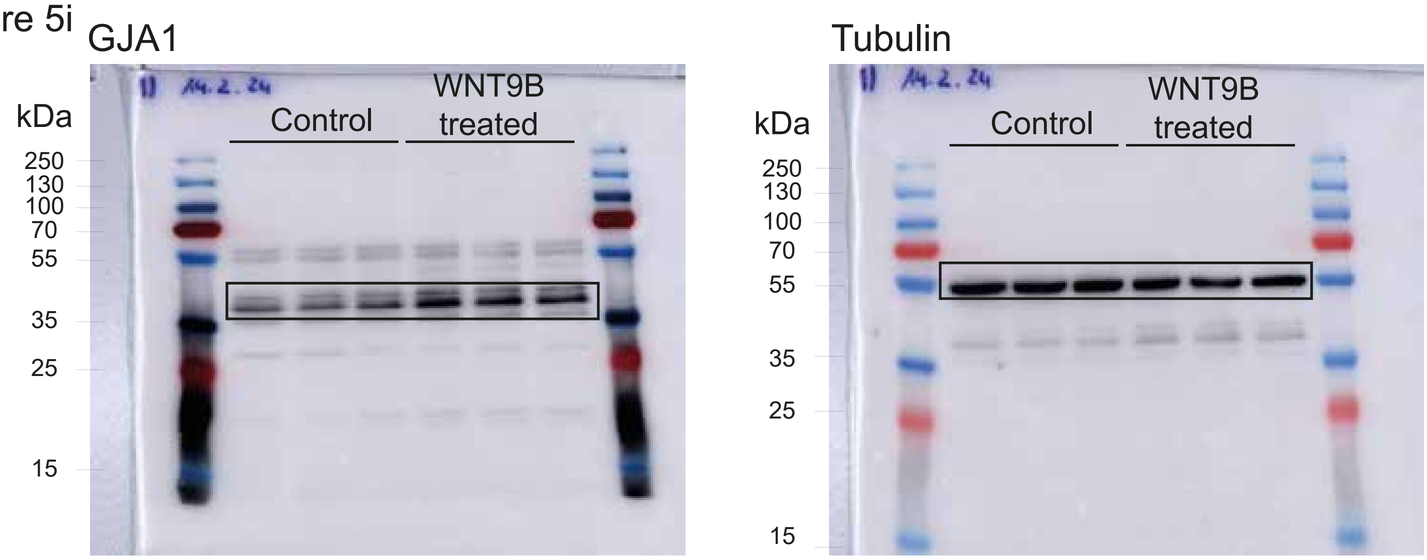

Figure 5j

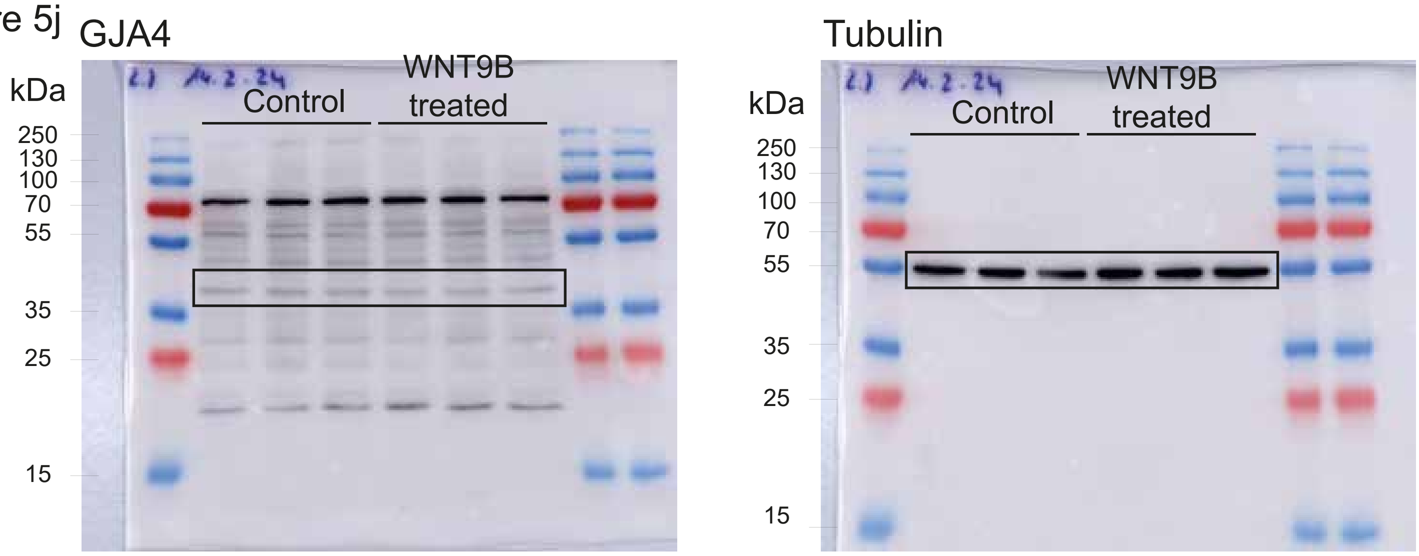

Figure 5k

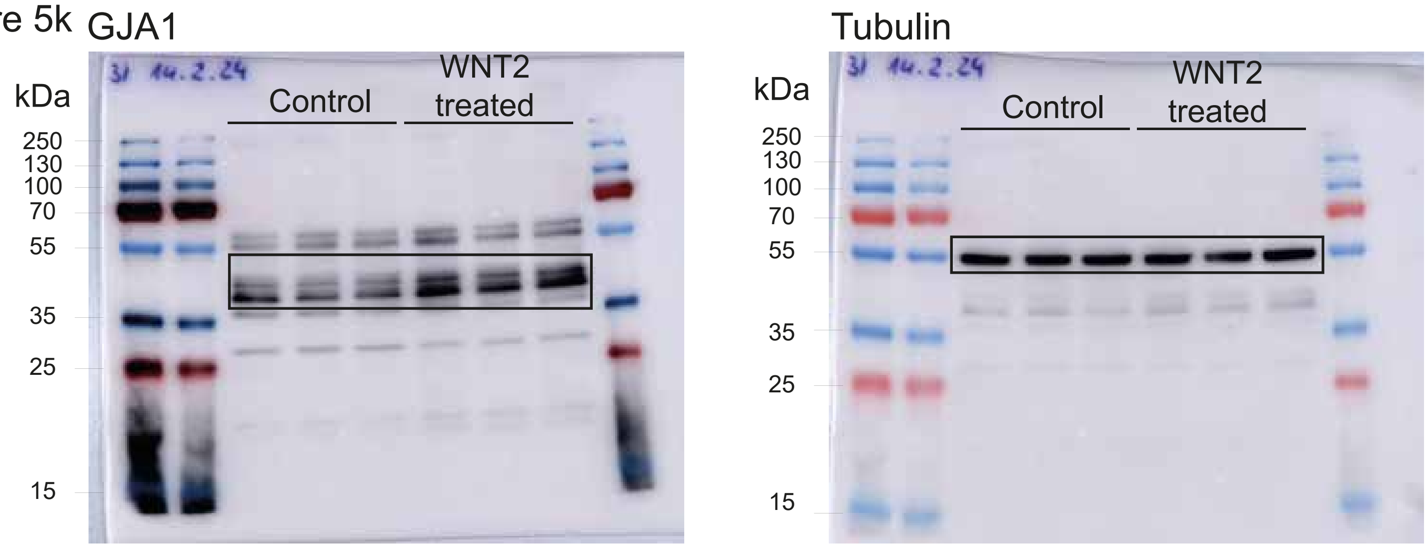

Figure 5l

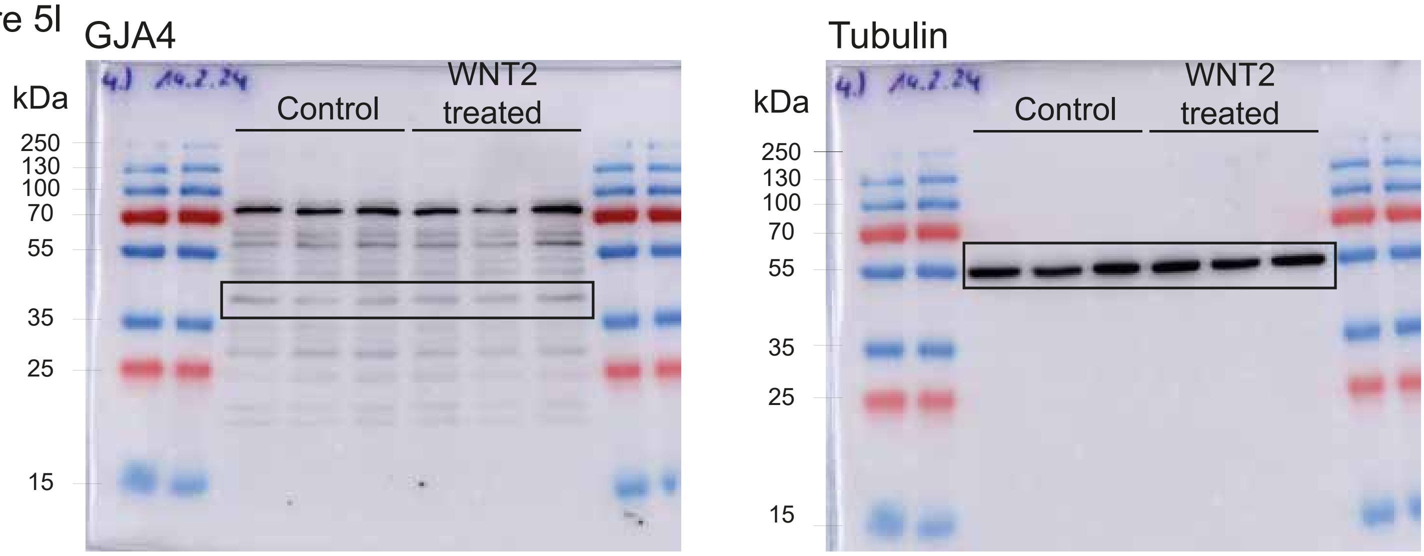

Supplementary Figure 2n

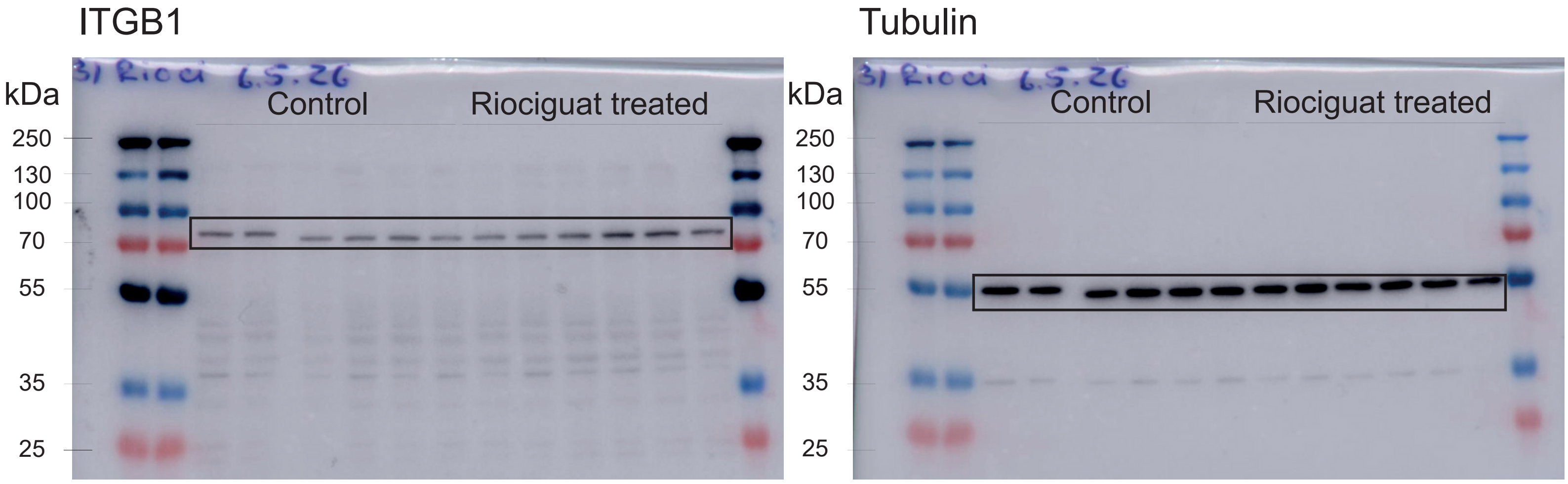

Supplementary Figure 2o

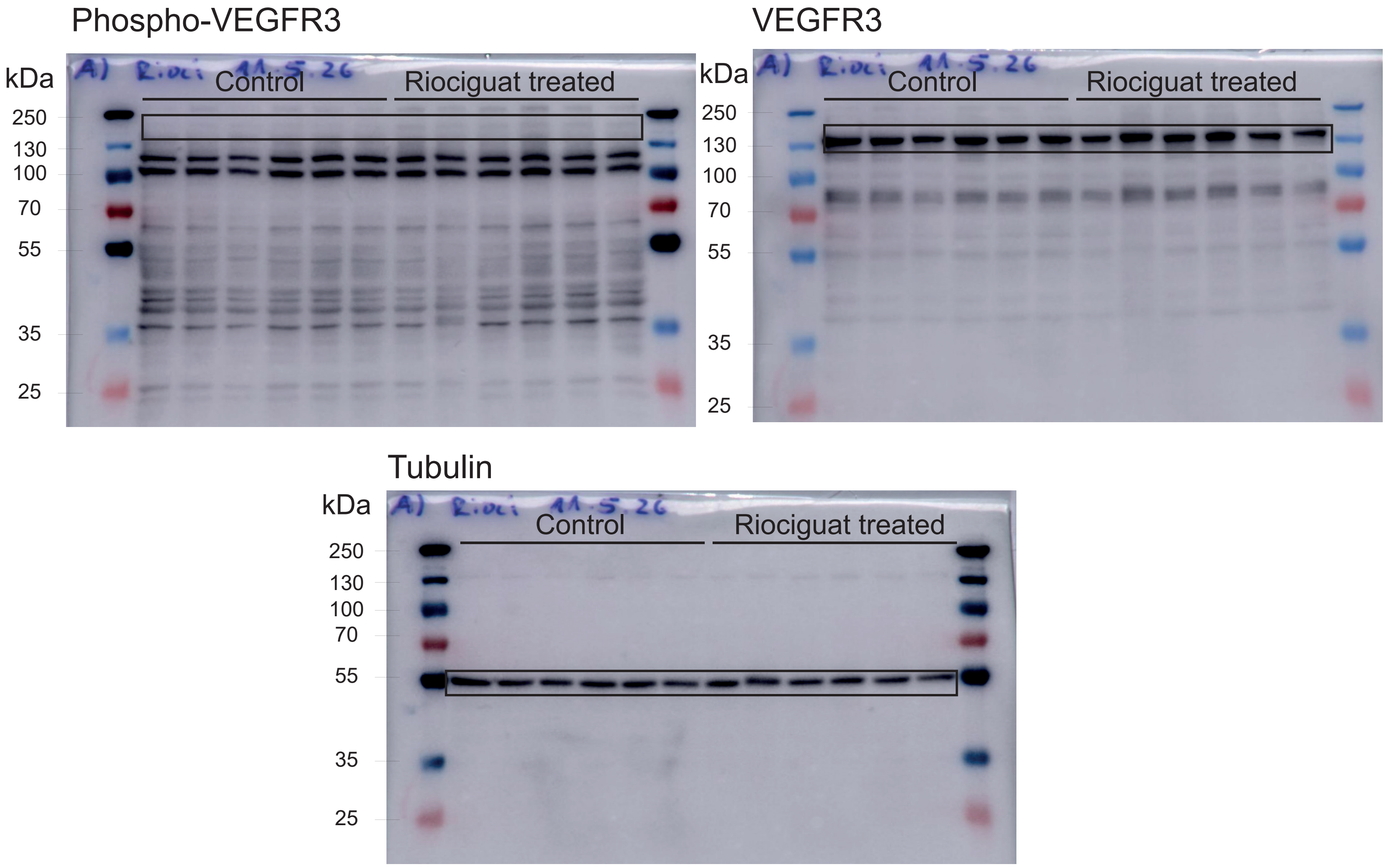

Supplementary Figure 11f,i

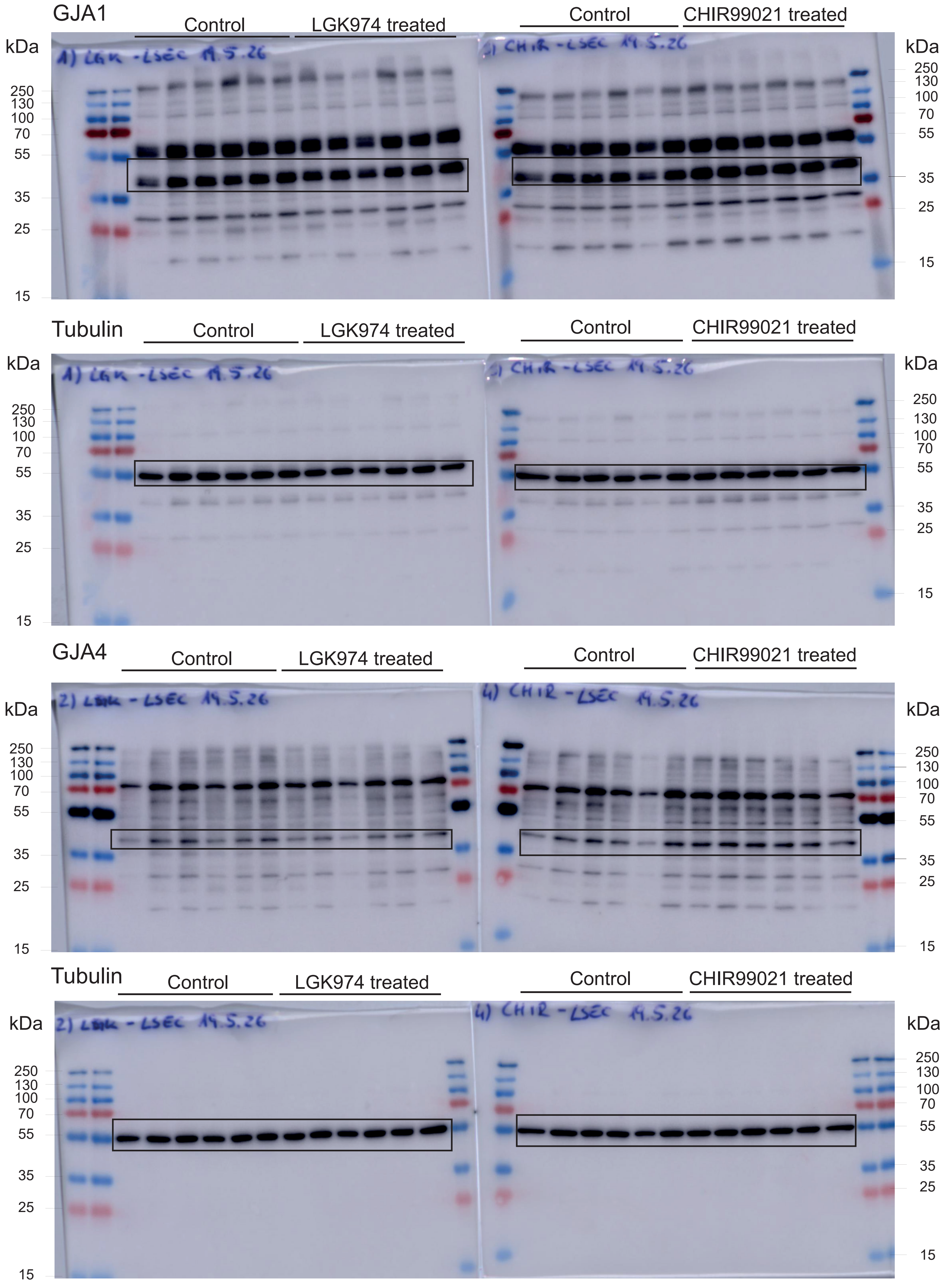

Supplement: Supplementary file 5 [file 41467_2026_76966_MOESM9_ESM.zip › Unprocessed Western blots.pdf]
